# Force-modulated reductive elimination from platinum(ii) diaryl complexes†

**DOI:** 10.1039/d1sc03182a

**Published:** 2021-07-26

**Authors:** Yichen Yu, Chenxu Wang, Liqi Wang, Cai-Li Sun, Roman Boulatov, Ross A. Widenhoefer, Stephen L. Craig

**Affiliations:** Department of Chemistry, Duke University Durham North Carolina 27708 USA ross.widenhoefer@duke.edu stephen.craig@duke.edu; Department of Chemistry, University of Liverpool Crown Street Liverpool L69 7ZD UK boulatov@liverpool.ac.uk

## Abstract

Coupled mechanical forces are known to drive a range of covalent chemical reactions, but the effect of mechanical force applied to a spectator ligand on transition metal reactivity is relatively unexplored. Here we quantify the rate of C(sp^2^)–C(sp^2^) reductive elimination from platinum(ii) diaryl complexes containing macrocyclic bis(phosphine) ligands as a function of mechanical force applied to these ligands. DFT computations reveal complex dependence of mechanochemical kinetics on the structure of the force-transducing ligand. We validated experimentally the computational finding for the most sensitive of the ligand designs, based on MeOBiphep, by coupling it to a macrocyclic force probe ligand. Consistent with the computations, compressive forces decreased the rate of reductive elimination whereas extension forces increased the rate relative to the strain-free MeOBiphep complex with a 3.4-fold change in rate over a ∼290 pN range of restoring forces. The calculated natural bite angle of the free macrocyclic ligand changes with force, but ^31^P NMR analysis and calculations strongly suggest no significant force-induced perturbation of ground state geometry within the first coordination sphere of the (P–P)PtAr_2_ complexes. Rather, the force/rate behavior observed across this range of forces is attributed to the coupling of force to the elongation of the O⋯O distance in the transition state for reductive elimination. The results suggest opportunities to experimentally map geometry changes associated with reactions in transition metal complexes and potential strategies for force-modulated catalysis.

## Abstract 2

The influence of mechanical force on the rates of model reductive elimination reactions depends on the structure of the force-transducing ligand and provides a measure of geometry changes upon reaching the transition state.

## Graphical abstract

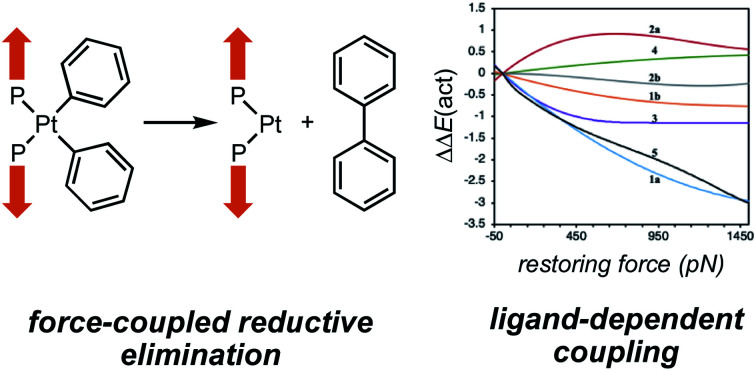

## Introduction

Over the last decade or so, coupled mechanical forces have been used to drive a range of targeted covalent responses in isolated polymers and in bulk polymeric materials (covalent polymer mechanochemistry).^1–3^ Mechanochemical strategies continue to evolve, including their recent use in biasing and probing reaction pathways,^4,5^ the release of small molecules and protons,^6–8^ stress reporting,^9–13^ stress strengthening,^14–16^ degradable polymers,^17,18^ and fundamental studies of polymer behaviour under load.^19^ In organic reactions, mechanochemical coupling has been investigated in simple bond dissociation reactions^20–24^ and in a wide variety of reaction classes with respect to regiochemistry,^25–28^ orbital symmetry,^25,29–31^ stereochemistry,^32,33^ supramolecular architecture,^34,35^ dynamic effects,^36,37^ and the alignment and/or loading of scissile bonds with applied tension.^38,39^ Unlike their organic counterparts, however, reported mechanochemical reactions in organometallic complexes involve almost entirely the direct, forced dissociation of a ligand. Examples include some of the earliest examples of polymer mechanochemistry,^40–43^ the release of latent catalysts,^44–46^ and as a means of generating colorimetric responses.^47–50^ In an emerging complementary strategy, a force applied to an intact ligand scaffold tunes reactivity at the coordinated metal center. In particular, force applied to a chiral ligand was shown to influence the enantioselectivity of enantioselective Heck arylations and Trost allylic alkylations.^51^

**Fig. 1:**
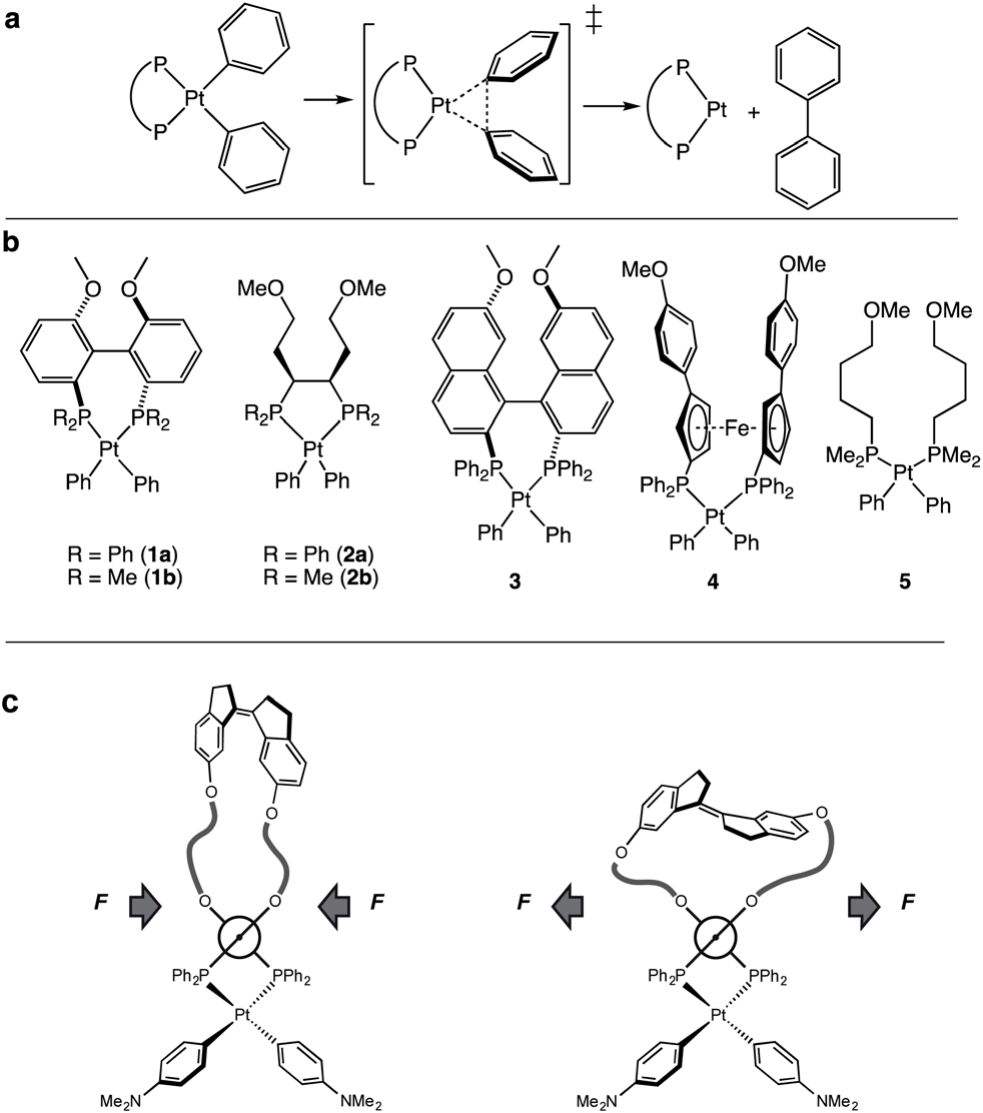
(a) Concerted reductive elimination of biphenyl from platinum diphenyl bisphosphine complexes. (b) Pt-bisphosphine complexes for computational study. (c) Schematic representation of the method of applying a compression or extension force on diaryl platinum bisphosphine complexes with a *Z* and *E* isomer of a force probe.

Because ligand structure and geometry directly impact the reactivity of organometallic complexes, mechanically coupled ligands offer the potential to externally regulate organometallic reactivity, if fundamental structure–reactivity relationships can be established. The use of force would complement other strategies for externally triggered reactivity, including those based on light, pH, metal-ion coordination, and redox changes.^52–56^ We therefore sought to extend the study of force-coupled ligands to their use in elementary transformations that occur within a structurally well-defined transition metal complex whose force-free reaction mechanisms and reactivity are well characterized. Toward these objectives, we have previously performed chemomechanical analysis^57^ of the oxidative addition of bromobenzene to low-valent Pd(0) complexes^58^ and sought to extend these analyses to the C(sp^2^)–C(sp^2^) reductive elimination from bisphosphine platinum complexes (Fig. 1a). Reductive elimination is one of the most important carbon–carbon bond forming processes in cross-coupling reactions,^59^ often closing catalytic cycles initiated by oxidative addition.

To enumerate the principles of force-reactivity coupling in reductive elimination, we computed force-dependent activation energies, Δ*E*_act_(*f*), of the reductive elimination of biphenyl from 7 platinum diaryl bisphosphine complexes containing mono- or bidentate phosphine ligands (Fig. 1b). We then validated these results experimentally for the complex with the greatest predicted force sensitivity, namely that based on a MeO-Biphep ligand (**1a**; Fig. 1c) employing molecular force-probe ligands. We chose platinum(ii) diaryl complexes for this study because their stability^60^ allows kinetic analysis at convenient temperatures from isolable reactants and because their concerted, unimolecular mechanism of reductive elimination^60–66^ increases the reliability of computed Δ*E*(*f*) and molecular interpretation of the measured kinetics.

**Fig. 2:**
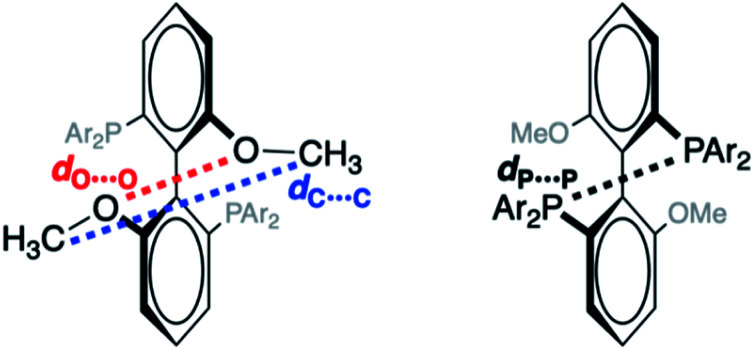
Interatomic distances used to characterize force-dependent changes in geometry of MeO-Biphep ligand, shown in front and rear view for clarity. Constraining force applied to the terminal C atoms of the methoxy groups (corresponding to *d*_C_⋯_C_, left).

**Scheme 1:**
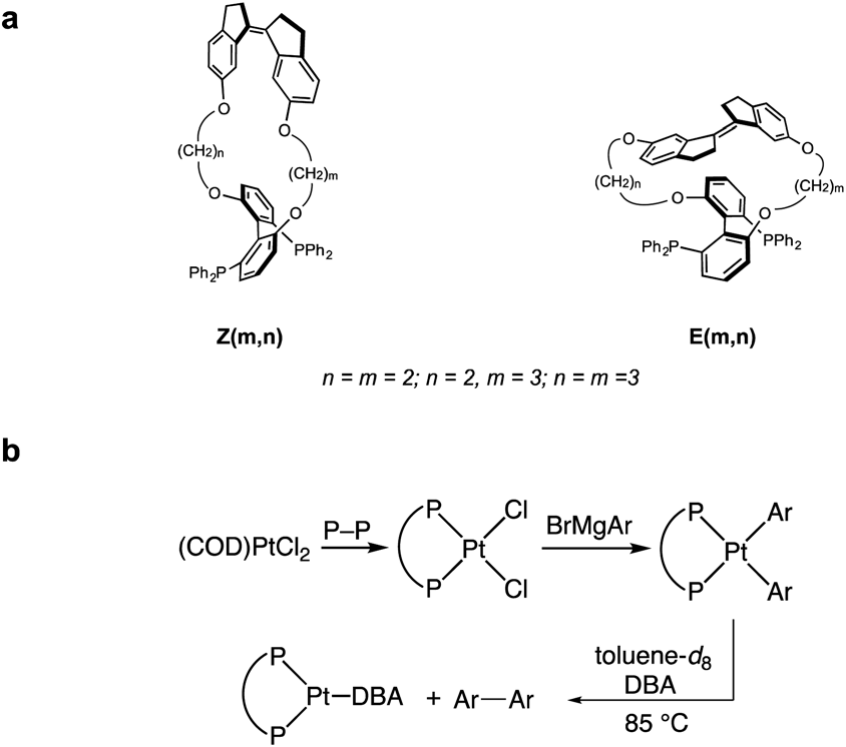
(a) Structure of force probe ligands tested experimentally. (b) Synthesis and reductive elimination of diaryl platinum bisphosphine complexes containing force probe ligands. COD = cyclooctadiene; DBA = dibenzylideneacetone; Ar = 4-C_6_H_4_NMe_2_.

Our calculations revealed a significant dependence of Δ*E*(*f*) on the ligand structure despite the same reaction mechanism and similar geometrical parameters within the first coordination sphere of Pt. We then employed a previously described strategy to experimentally validate the computed Δ*E*_act_(*f*) for (MeO-Biphep)PtPh_2_ (**1a**) at forces between −65 pN and 220 pN (negative force corresponds to compression along the C⋯C vector, Fig. 2). We employed macrocyclic force probe ligands *E*(*m*,*n*) and *Z*(*m*,*n*) comprising stiff stilbene (1,1′-biindane)^3^ tethered to the MeO-Biphep moiety (Scheme 1). In these macrocycles the *Z* and *E* isomers of stiff stilbene subject the oxygen atoms of the bisphosphine moiety to a compressive or stretching force, respectively, whose magnitude is controlled by the tether length. Previous studies on a mechanochemical electrocyclic ring opening have shown that the effect of force applied intramolecularly by stiff stilbene is effectively equivalent to that of the same force applied externally, for example by the tension in a stretched polymer strand.^67–70^ The force probe ligands therefore provide a convenient method to apply well-defined force to metal complexes under conditions that allow their reactivity to be studied using conventional spectroscopic methods, while yielding insights into how reactivity would be influenced by forces experienced, *e.g.*, in a deformable solid support.

## Results

### DFT computations of Δ*E*_act_(*f*)

**Fig. 3:**
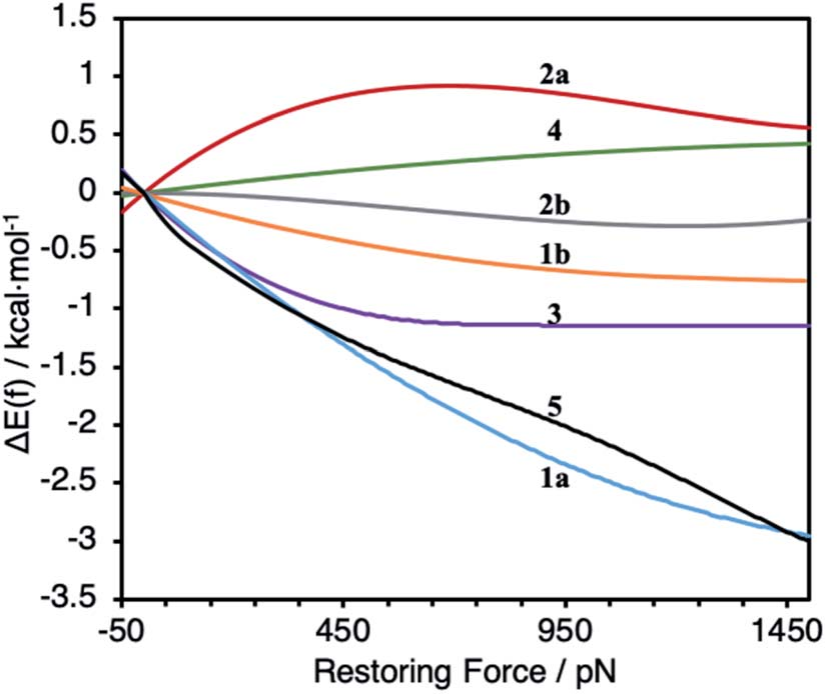
Calculated force-dependent changes in the activation energy, ΔΔ*E*(*f*), for the reductive elimination of biphenyl from platinum bisphosphine complexes **1–5**.

To probe the effect of ligand structure on the efficiency of force transduction, we calculated Δ*E*_act_(*f*) for the reductive elimination of biphenyl from platinum bisphosphine complexes **1–5** using a previously reported and validated method^69–71^ with force applied to the C atoms of the methoxy groups (Fig. 2) from −50 pN (compression) to 1.5 nN (extension).^19,70^ All computations were at the B3LYP/def2SVP level of DFT in the gas phase. Computed force-dependent kinetics (Fig. 3) ranged from modest acceleration by tensile force, most pronounced in **1a** and **5**, through rare^39,58^ albeit weak deceleration in **2a** and **4**. Importantly, biphenyl elimination in all complexes and at all forces studied traversed a single transition state. In the absence of force, moving from ground state to transition state occurred with concomitant opening of the P–Pt–P angle and elongation of the P⋯P separation by between 0.15 and 0.02 Å, depending on the ligand (Table S21†). Elongation of the P⋯P separation was uncorrelated with changes in the _OMe_C⋯C_MeO_ distance, which defines the pulling axis, due to each state comprising multiple thermally accessible conformers in every complex. Such a lack of correlation in strain-free ensembles is common in organic reactants.^39,72^

Applying tensile force at C_MeO_ elongated C⋯C and O⋯O distances in both reactant and transition states. Across all forces and complexes studied, force-induced changes in the C⋯C and O⋯O distances correlated strongly (coefficient 0.941), suggesting that O⋯O coordinate is a useful proxy for estimating the magnitude of kinetically-significant strain imposed on the complex. The effect of force on the geometry of the 1^st^ coordination sphere of Pt was more complex. Stretching force increases the P–Pt–P angle in complexes **1**, **3** and **5** by 4–6°/nN, does not affect it in **2**, and decreases it by 1°/nN in **4**. Unlike several organic reactions,^73^ the compliances of any of the 4 coordinates analyzed above are nearly identical in the reactant and the transition states of any of the 7 complexes. This means that the pulling axis is nearly orthogonal to the reactive mode in the vicinity of the transition state,^74^ even for complexes such as **1a** and **5** whose reaction kinetics depends strongly on force.

### DFT calculations of macrocyclic ligands *E*(*m*,*n*) and *Z*(*m*,*n*)

We sought to experimentally validate the calculated Δ*G*_act_(*f*)/force response of platinum MeOBiphep complex **1a**, which displayed the greatest sensitivity to force of the complexes investigated, by employing the macrocyclic force probe ligands *E*(*m*,*n*) and *Z*(*m*,*n*) (Scheme 1). Toward this objective, we calculated the force applied by the stiff stilbene to the oxygen atoms of the MeOBiphep moiety in these macrocycles. The size of the macrocyclic platinum diphenyl complexes precluded DFT calculations of their conformational ensembles. However, we previously demonstrated that the force imposed by stiff stilbene on the biphep moiety in free macrocyclic ligands similar to those employed here was within ±20 pN of the force estimated in palladium dichloride complexes of the same ligands.^58^ Consequently, we optimized full conformational ensembles of free macrocyclic ligands at B3LYP/6-311+G(d) in the gas phase and compared their ensemble-average _biphen_O⋯O_biphen_ distances to the _OMe_O⋯O_MeO_ distance in MeOBiphep under the force of −0.15–1.5 nN calculated at the same level of theory (Table S4†). We assumed that the force acting on the OBiphep moiety of each macrocycle equals the force needed to be applied externally to the C_MeO_ atoms of free MeOBiphep to stretch or compress its O⋯O distance to the same value as in each macrocycle. In other words, we used the computed relationship between applied force and O⋯O distance of MeOBiphep as a “calibration curve”^39,70^ to estimate the force applied to the same moiety by stiff stilbene in the macrocycle, which ranged from −65 (*Z*(2,2)) to 228 (*E*(2,3)) pN (Table 1). We previously demonstrated^39^ that this “calibration curve” approach yielded force estimates that were within 10 pN of the force derived from detailed vibrational analysis for any internuclear distance whose force-dependent variation correlated strongly with that of the constrained distance. The O⋯O distance of our macrocycles meets this criterion.

**Table 1:** First-order rate constants for the reductive elimination of (P–P)PtAr_2_ complexes in toluene-*d*_8_ at 85 °C

Entry	(P–P)	Est. applied force (pN)	(10^5^)*k* (s^−1^)
1	*Z*(2,2)	−65	5.8 ± 0.1^a^
2	*Z*(3,3)	−3	5.89 ± 0.05^b^
3	MeOBiphep	0	6.92 ± 0.05
4	*E*(3,3)	130	14.8 ± 0.2^b^
5	*E*(2,3)	228	19.5 ± 0.6^b^

a Average of three independent experiments.

b Average of two independent experiments.

### Experimental validation of Δ*E*_act_(*f*) for **1a**

The requisite platinum diaryl bis(phosphine) complexes (P–P)PtAr_2_ (Ar = 4-C_6_H_4_NMe_2_; P–P = force probe ligand) were synthesized in two steps from the reaction of force probe ligand with (COD)PtCl_2_ (COD = 1,5-cyclooctandiene) to form dichloride complexes (P–P)PtCl_2_ followed by transmetallation with 4-dimethylaminophenyl magnesium bromide (Scheme 1). Here, dimethylaminophenyl groups were employed in place of the phenyl groups of **1a** to lower the energy barrier for reductive elimination and avoid complications in the kinetic analysis of reductive elimination.^60^ The platinum dichloride complex of the most extended *E*(2,2) ligand isomerized to the *Z*(2,2) analog within 10 min at room temperature, which precluded the generation of the platinum diaryl complex of the *E*(2,2) ligand.

The platinum dichloride and diaryl complexes were characterized in solution by ^1^H and ^31^P NMR spectroscopy. Unfortunately, despite extensive efforts we were unable to obtain X-ray structures of platinum diaryl and dichloride complexes containing force probe ligands. However, the DFT estimate of the variation of the P–Pt–P angle among the 5 macrocycles being ∼1.5° is consistent with the narrow range of one-bond platinum-phosphorous coupling constants (^1^*J*_P–Pt_) across the series of both (P–P)PtCl_2_ (^1^*J*_P–Pt_ = 3645–3671 Hz) and (P–P)PtAr_2_ complexes (^1^*J*_P–Pt_ = 1763–1773 Hz). The conclusion is based on the high sensitivity of ^1^*J*_P–Pt_ of electronically and sterically homologous bis(phosphine) platinum complexes to the P–Pt–P angle.^75–77^ For example, ^1^*J*_P–Pt_ varies by >230 Hz for the ∼12° increase in the P–Pt–P angle (∼19 Hz/°) across the structurally characterized platinum dichloride complexes [Ph_2_P(CH_2_)_*x*_PPh_2_]PtCl_2_ (*x* = 3–5).^76,77^

**Fig. 4:**
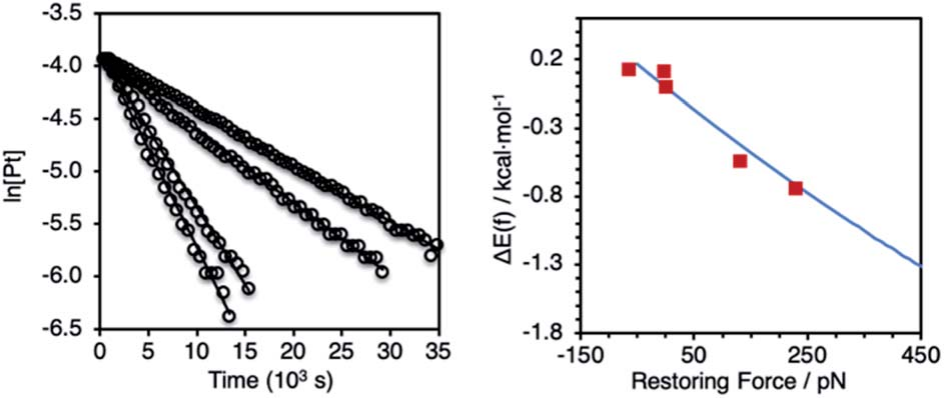
Left: representative first–order plots of the reductive elimination of (P–P)PtAr_2_ complexes. Right: comparison between the calculated force response for the reductive elimination of complex **1a** (blue curve from Fig. 3) and experimentally determined values for reductive elimination of (P–P)PtAr_2_ complexes (red squares).

Solutions of (P–P)PtAr_2_ (16 mM) and dibenzylidene acetone (DBA; 1 equiv.) in toluene-*d*_8_ were heated at 85 °C and analyzed periodically by ^1^H NMR spectroscopy (Scheme 1). In each case, the disappearance of (P–P)PtAr_2_ obeyed first-order kinetics to ≥3 half-lives to form 4,4′-bis(dimethylamino)-1,1′-diphenyl and (P–P)Pt(DBA) as the exclusive organic and organometallic product, respectively (Fig. 4 and Table 1). DBA was employed as a trapping ligand to prevent secondary decomposition of the (P–P)Pt(0) species released *via* reductive elimination.^60–66^ The rate of reductive elimination increased by a factor of 3.4 with increasing extension force in the order *Z*(2,2) < *Z*(3,3) < MeOBiphep < *E*(3,3) < *E*(2,3). The correlation between the estimated force and ln(*k*) determined for the reductive elimination of the (P–P)PtAr_2_ complexes (red squares, Fig. 4) agreed very well with Δ*E*_act_(*f*) calculated for the reductive elimination of **1a** (blue line, Fig. 4) for both compressive and stretching force.

## Discussion

**Fig. 5:**
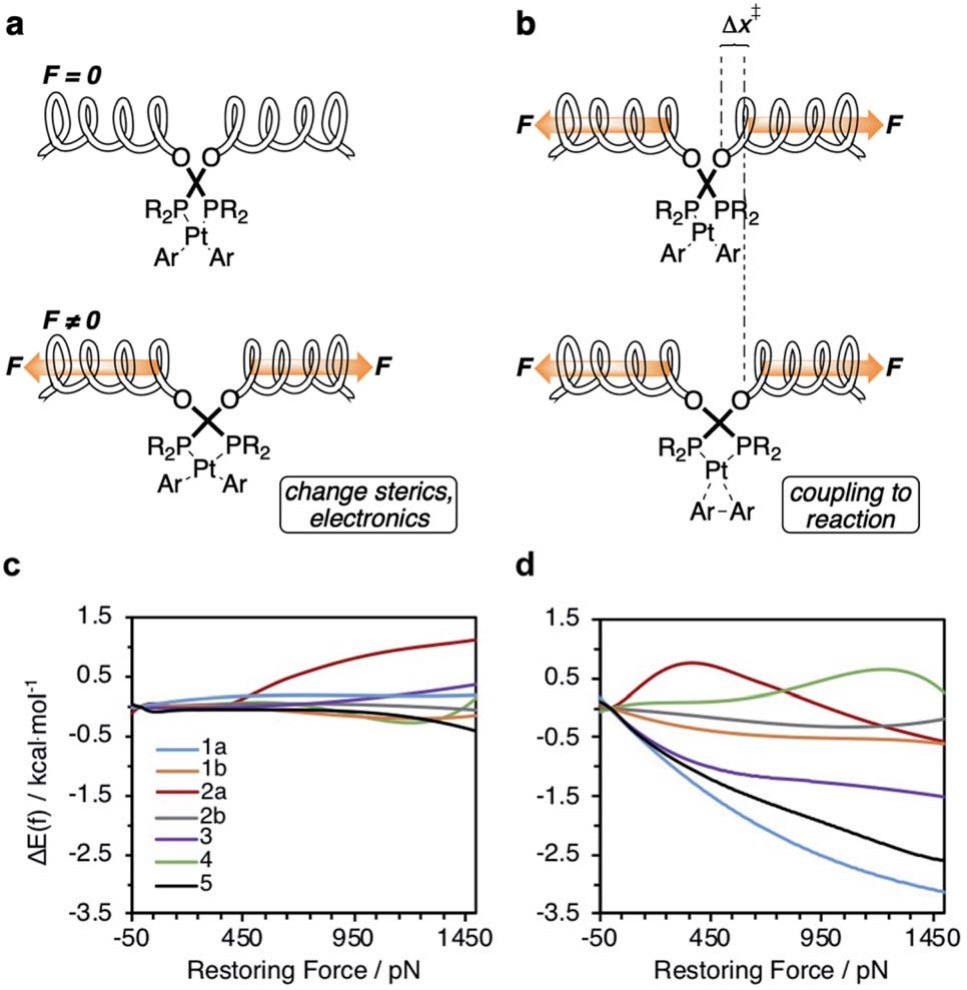
(a and b) The constraining potential that applies force to a ligand (depicted here by a coupled spring) affects reactivity of the metal complex by: (a) distorting the geometry of the complex, thereby changing its stereoelectronic properties in the force-coupled state (bottom) relative to the force-free state (top), and (b) coupling the structural differences between the reactant (top) and the transition states (bottom) to changes in the energy of the constraining potential. (c and d) Calculated force-dependent changes in the contributions to Δ*E*_act_ (Fig. 2a) of the reactive site strain (c) and of the constraining potential (d) for complexes **1–5**.

The well-established elementary nature of reductive elimination from diaryl platinum bis(phosphine) complexes^60–66^ (*i.e.*, free from dynamic structural rearrangements^60,63–66^) facilitates quantitative molecular interpretation of force/rate correlations in the reductive elimination of (P–P)PtAr_2_ complexes. To a good approximation, force-dependent activation energies are a sum of two contributions.^3,74^ One captures the kinetic effects of strain imposed on the reactive moiety and the other of changes in strain energy of all other molecular degrees of freedom of the molecule and its surroundings (represented by spring in Fig. 5a).^78,79^ The former contribution is reminiscent of entatic states of bioinorganic chemistry, with the altered catalytic activity arising from the changed sterics of the active site or the relative energies and shapes of molecular orbitals that participate in catalytic reactions.^80^ In the (P–P)PrAr_2_ (P–P = force probe ligand) complexes, this contribution reflects how much the kinetics is affected by distortions of the P–Pt–P angle or P–Pt bonds caused by applied force. This contribution is insensitive to how the geometry of the reactive site changes throughout the reaction.

The second contribution is directly proportional to the structural differences along the pulling axis between the reactant and the rate-limiting transition states. For example, a transition state that is longer than the reactant along the pulling axis is stabilized by stretching force, because elongation of the reactive site accompanying its formation allows partial relaxation of all other strained molecular coordinates whose bonding does not change during the reaction. This contribution tends to dominate mechanochemical kinetics of organic reactions and its importance increases with force.^3,39^

Our calculations indicate the platinum bisphosphine complexes **1–5** follow the same general trend: the molecular strain imposed by force on either the reactant (Fig. 5a) or the transition state negligibly affects the kinetics as evident by the lack of correlation between Δ*E*_act_(*f*) and the molecular-strain component (average correlation coefficient 0.05). Conversely, changes in the energy of the constraining potential (spring in Fig. 5b) dominate Δ*E*_act_(*f*) (average correlation coefficient 0.86) and account for >90% of variation of Δ*E*_act_(*f*) across the series. In other words, the variation in the mechanochemical sensitivity of the rate of reductive elimination from complexes **1–5**, from 30-fold acceleration to 2-fold deceleration per 1 nN of stretching force despite the same reaction mechanism and similar transition state, is attributable to how well each ligand couples the structural changes in the 1^st^ coordination sphere to the pulling coordinate (*i.e.*, the pair of atoms across which the force is applied). For bidentate ligands, capacity to accommodate the opening of the P–Pt–P angle in the transition state is a secondary contribution.

In the simplest case of monodentate trialkyl phosphines (**5**, Fig. 1), the coupling is very similar to that established in diverse S_N_2 displacement reactions at Si, P and S atoms.^39,81^ The changes in C⋯C, P⋯P and P–Pt–P coordinates are proportional. Biphep (**1**) and binap (**3**) ligands restrict the opening of the P–Pt–P angle in the transition state from 7.6° to 2–3°, depending on the remaining P substituents, but are reasonably effective at coupling local and remote changes. *cis*-DPPE (**2a**) and dmpe ligands (**2b**) are both poor at accommodating P–Pt–P angle opening and inefficient at coupling it to changes in the constrained coordinate. The former is evident from the very small opening of the P–Pt–P angle during strain-free reaction and the latter from the ∼10-fold higher apparent stiffness of the P–Pt–P angle in DPPE and dmpe ligands compared to biphep analogs when tensile force is applied to the C_OMe_ atoms. The DPPF derived ligand of complex **4** is unusual in that the changes in the P–Pt–P angle and P⋯P distance are inversely correlated with changes in the C⋯C and O⋯O distances. Tensile force stretching the C⋯C distance simultaneously contracts both P–Pt–P and P⋯P coordinates, while opening up of the P–Pt–P angle in the transition state contracts the C⋯C and O⋯O distances.

It is productive to contrast the effect of force on the reaction kinetics reported here with the historically important analysis of the effects of the bite angle of chelating bisphosphine ligands on the reactivity of transition metal complexes in the context of perturbations of the P–M–P angle.^73,82,83^ The energy decomposition analysis shown in Fig. 5 and measured ^1^*J*_P–Pt_ couplings indicate that these classical P–M–P bite angle effects are not responsible for the observed trends in reactivity. The concepts of natural bite angle and ligand flexibility developed by Casey, however, acknowledge the potential modulation of the reactivity of a transition metal complex by a bisphosphine ligand in a manner distinct from perturbations of the P–M–P angle in the nascent complex. Conversely, absent from previous analyses of bite angle effects are efforts to correlate reactivity to the strain within the ligand backbone resulting from metal-imposed deviation of ligand geometry away from the preferred natural bite angle.^84,85^ The framework and results presented here therefore extend these concepts and demonstrate that coupling of mechanical force imposed on the bisphosphine backbone measurably changes the reactivity of the metal center even in the absence of discernible changes in ground state metal–ligand geometry. The change in reactivity observed here is attributed to a structural perturbation that occurs beyond the catalyst active site: the P–Pt–P angle within the complex does not change (as supported by the relative invariance within the experimental one-bond coupling constants), but the molecular strain outside the active site does. The reactivity is driven by the relaxation of the outer-sphere strain in the transition state relative to the ground state.

## Conclusions

The DFT-level calculations of force-dependent activation energies, Δ*E*_act_(*f*), for reductive elimination from 7 Pt bisphosphine complexes (**1–5**) and experimental validation of the computed trend for MeOBiphep complex **1a** demonstrate that mechanochemical kinetics of this reaction is sensitive to the molecular geometry away from the reactive site to the extent usually inaccessible in organic reactions. We speculate that these results augur well for the viability of multi-state catalysts that are switched by mechanical force.

Transition metal-catalyzed processes typically comprise a number of discrete elementary transformations, and these elementary steps are often affected differently by ligand geometry.^73,82,83^ For example, reductive elimination often closes catalytic cycles initiated by oxidative addition, and these two transformations often display opposing responses to ligand bite angle perturbations.^73,82,83,86–91^ In such cases, the most effective achievable single-state catalysts likely represent a compromise among the various microscopic steps.^73,82,83,92–95^ For this reason, catalysts systems that could be reversibly switched between force-coupled geometries that are optimized for specific steps within the catalytic cycle on the timescale of catalytic turnover^96^ or polymer enchainment^97,98^ have the potential to circumvent the inherent compromise associated with geometrically static transition metal catalysts. Toward this broader objective, forces on the order of ∼100 pN similar to those employed here have been shown to be attainable reversibly and repeatably in elastomers under tension,^57^ including in a range of soft devices that respond to a variety of triggers.^99,100^ In conjunction with our previous work,^58^ we have now demonstrated that the rates of two elementary transformations, namely reductive elimination and oxidative addition, are affected differentially by force.

The use of molecular design to impose controlled, intramolecular forces, as employed here, might also be useful for elucidating aspects of mechanistic pathways that are otherwise difficult to probe experimentally.^101^ Analysis of force-dependent kinetics allows structural changes between reactant and transition states to be quantified in much the same way that one would apply a substituent effect in a linear free energy relationship to quantify changes in charge distribution or a kinetic isotope effect to quantify changes in bonding. Complexes that involve a minimal initial structural perturbation and are chosen to ensure a consistent mechanism across a range of forces are particularly well suited for such studies. Application of the approach described here to other metal complexes should provide mechanistic insights into a broad range of organometallic transformations that would complement traditional mechanistic studies.

Finally, we note that the combined computational and experimental methods employed here offer ongoing opportunities to refine mechanistic understandings of ligand effects in organometallic catalysis. In the current system, the resting state of the complex is negligibly distorted by force. This obviously need not be general, and other metal–ligand scaffolds with more pliable bonding geometries, such as octahedral and trigonal bipyramidal complexes possessing accessible equatorial/equatorial and equatorial/axial binding sites, might lead to increasingly sensitive to mechanical force *via* force-induced perturbations of ground state geometry.^84,85^

## Data availability

All associated computational and experimental data is provided in the ESI.

## Author contributions

S. L. C. and R. A. W. conceived the project. S. L. C., R. A. W., and Y. Y. designed the experiments. R. B. designed the computational approach. Y. Y. and L. W. conducted the experiments. C. W. and C.-L. S. conducted the computations. Y. Y., R. B., S. L. C., and R. A. W. analysed the data and wrote the paper.

## Conflicts of interest

There are no conflicts to declare.

## Supplementary Material

SC-012-D1SC03182A-s001
