# Supplementary material for: Force-modulated reductive elimination from platinum(ii) diaryl complexes
Source: Chem Sci. 2021 Jul 26;12(33):11130–7. doi: 10.1039/d1sc03182a (PMC8386663; doi:10.1039/d1sc03182a)
Supplement: SC-012-D1SC03182A-s001 [file SC-012-D1SC03182A-s001.pdf]

Electronic Supplementary Material (ESI) for Chemical Science.
This journal is © The Royal Society of Chemistry 2021

Electronic Supplementary Information

Force-Modulated Reductive Elimination from Platinum(II) Diaryl Complexes

Yichen Yu,[1] Chenxu Wang,[2] Liqi Wang,[1] Caili Sun,[2] Roman Boulatov,[2*] Ross A. Widenhoefer,[1*] and Stephen L. Craig[1*]

[1]Department of Chemistry, Duke University, Durham, North Carolina 27708, USA.

[2]Department of Chemistry, University of Liverpool, Crown Street, Liverpool L69 7ZD, UK.

Table of Contents

General Methods	S2
Synthesis of (P–P)$PtCl_2$ complexes	S2-S5
Synthesis of (P–P)$PtAr_2$ complexes	S6-S8
Kinetic analysis of reductive elimination	S9-S11
Computed O···O distances and restoring forces for force probe ligands	S12-S51
References	S52
Scans of NMR Spectra	S53-S67

General Methods

All reactions were performed under a nitrogen atmosphere in flame-dried glassware employing standard Schlenk or glovebox techniques unless otherwise noted. Nitrogen-flushed plastic syringes and oven-dried stainless steel cannulas were employed for reagent transfer. NMR spectra were obtained at 25 °C unless noted otherwise. ^{1}H and ^{13}C chemical shifts are referenced to the solvent residual peaks and ^{31}P NMR spectra were referenced using absolute frequency referencing in Mnova software or from trimethylphosphine oxide internal standard. Chemical shifts are given in units of ppm (δ) and coupling constants (*J*) in Hz. Multiplicities are assigned as singlet (s), doublet (d), triplet (t), quartet (q), pentet (p), multiplet (m), or broad (br). For high-temperature NMR experiments, probe temperature was determined from a single scan of neat ethylene glycol with accuracy of ± 1 °C.

Anhydrous solvents were obtained either from Sigma-Aldrich in Sure/Seal™ containers or were dried and degassed using an Innovative Technologies PureSolv solvent purification system. All deuterated solvents were obtained from Cambridge Isotope Laboratory and were dried using activated 3Å molecular sieves. Freshly opened anhydrous toluene-d_8 was degassed via three freeze-pump-thaw cycles and stored in a glovebox. CD_2Cl_2 was dried over CaH_2 and distilled prior to use. All other reagents were purchased from major chemical suppliers and were used as received unless otherwise noted. (*R*)-MeO-BIPHEP was purchased from Strem. Force probe ligands were synthesized employing published procedures.[S1, S2]

Synthesis of (P–P)PtCl$_2$ complexes

Synthesis of (P–P)PtCl$_2$: General procedure 1. A solution of (COD)PtCl$_2$ (COD = 1,5-cyclooctadiene (1 equiv), bisphosphine ligand (1.05 eq) in CH_2Cl_2 (3 mL) was stirred at room temperature for 1 h. The resulting yellow solution was diluted with diethyl ether (20 mL) and the resulting suspension was filtered. The white precipitate was washed with diethyl ether and recrystallized by layering a saturated CH_2Cl_2 solution with diethyl ether to give pure (P-P)PtCl$_2$.

(MeOBiphep)PtCl$_2$.[S3] Complex (MeOBiphep)PtCl$_2$ was isolated as colorless crystals (261.1 mg, 99.6%) from reaction of (*R*)-MeO-BIPHEP (189.2 mg, 0.325 mmol) with (COD)PtCl$_2$ (115.7 mg, 0.309 mmol) employing general procedure 1. ^{1}H NMR (500 MHz, CD_2Cl_2): δ 7.80 (s, br, 4H), 7.64 (dd, *J* = 11.4, 7.1 Hz, 4H), 7.46 (t, *J* = 7.4 Hz, 2H), 7.40 (t, *J* = 7.6 Hz, 6H), 7.25 (t, *J* = 7.6 Hz, 4H), 6.95 – 6.88 (m, 2H), 6.67 (dd, *J* = 10.6, 7.6 Hz, 2H), 6.41 (d, *J* = 8.3 Hz, 2H), 3.47 (s, 6H). ^{13}C{^{1}H} NMR (126 MHz, CD_2Cl_2, J_{CP} not included): δ 157.97, 157.92, 157.88, 135.62, 135.58, 135.54, 131.86, 131.08, 129.65, 129.60, 129.55, 129.09, 129.05, 128.49, 128.44, 128.28, 128.24, 128.20, 127.61, 127.57, 127.52, 127.45, 127.40, 125.14, 125.11, 125.07, 124.47, 124.42,

123.92, 123.87, 113.08, 66.03, 55.44, 15.48. ^{31}P NMR (202 MHz, CD_2Cl_2): δ 8.01 (s, J_{PtP} = 3646 Hz). HRMS (ESI$^+$) calcd (found) for $C_{38}H_{32}ClO_2P_2Pt$ [M–Cl]$^+$: 813.1215 (813.1213).

[Z(2,2)]PtCl₂. Complex [**Z(2,2)**]$PtCl_2$ was isolated as a white solid (83.1 mg, 70.0%) from reaction of **Z(2,2)** (95.0 mg, 0.109 mmol) with (COD)$PtCl_2$ (38.9 mg, 0.104 mmol) employing general procedure 1. ^{1}H NMR (500 MHz, CD_2Cl_2): δ 7.84 (s, br, 4H), 7.72 – 7.65 (m, 4H), 7.56 (s, 2H), 7.50 – 7.38 (m, 8H), 7.34 (t, J = 7.7 Hz, 4H), 7.16 (d, J = 8.2 Hz, 2H), 6.88 (t, J = 8.2 Hz, 2H), 6.77 – 6.68 (m, 4H), 6.44 (d, J = 8.3 Hz, 2H), 4.14 – 3.93 (m, 8H), 2.97 – 2.74 (m, 8H). ^{13}C{^{1}H} NMR (126 MHz, CD_2Cl_2) δ 157.04, 156.99, 156.96, 141.79, 141.59, 135.81, 135.65, 135.61, 135.58, 131.94, 131.08, 129.73, 129.67, 129.63, 129.21, 129.18, 129.10, 129.06, 128.39, 127.58, 127.53, 127.49, 127.14, 127.09, 127.03, 126.14, 125.39, 125.36, 125.32, 124.42, 124.38, 123.87, 123.83, 114.99, 113.53, 109.08, 66.71, 66.49, 35.38, 30.06. ^{31}P NMR (202 MHz, CD_2Cl_2): δ 8.10 (s, $J_{Pt\text{-}P}$ = 3652 Hz). HRMS (ESI$^+$) calcd (found) for $C_{58}H_{48}ClO_4P_2Pt$ [M–Cl]$^+$: 1101.2367 (1101.2340).

[Z(3,3)]PtCl₂. Complex [**Z(3,3)**]$PtCl_2$ was isolated as a white solid (83.6 mg, 56.1%) from reaction of **Z(3,3)** (122.1 mg, 0.136 mmol) with (COD)$PtCl_2$ (48.0 mg, 0.128 mmol) employing general procedure 1. ^{1}H NMR (500 MHz, CD_2Cl_2): δ 7.85 (s, br, 4H), 7.65 (dd, J = 11.3, 7.4 Hz, 4H), 7.58 (s, 2H), 7.49 (t, J = 7.3 Hz, 2H), 7.46 – 7.41 (m, 4H), 7.38 (t, J = 7.5 Hz, 2H), 7.22 (t, J = 7.6 Hz, 4H), 7.14 (d, J = 8.2 Hz, 2H), 6.93 (td, J = 8.2, 2.3 Hz, 2H), 6.71 – 6.61 (m, 2H), 6.58 (dd, J = 8.3, 2.2 Hz, 2H), 6.33 (d, J = 8.3 Hz, 2H), 3.79 (dt, J = 12.0, 6.2 Hz, 2H), 3.66 (dt, J = 10.5, 7.0 Hz, 2H), 3.56 (dd, J = 8.2, 3.5 Hz, 4H), 2.98 – 2.75 (m, 8H), 1.96 – 1.79 (m, 4H). ^{13}C{^{1}H} NMR (126 MHz, CD_2Cl_2): δ 156.83, 156.37, 141.88, 140.98, 135.70, 135.46, 131.99, 131.11, 129.57, 129.53, 129.48, 129.44, 129.05, 129.01, 128.91, 128.88, 128.45, 128.41, 128.08, 127.65, 127.60, 127.56, 126.10, 124.98, 124.95, 124.91, 124.47, 124.43, 123.93, 123.89, 114.16, 112.61, 110.12, 100.93, 64.42, 63.73, 35.11, 31.25, 30.16, 28.71. ^{31}P NMR (162 MHz, CD_2Cl_2): δ 7.81 (J_{PtP} = 3655 Hz). HRMS (ESI$^+$) calcd (found) for $C_{60}H_{52}ClO_4P_2Pt$ [M–Cl]$^+$: 1129.2681 (1129.2687).

Synthesis of (P-P)PtCl₂: General Procedure 2. A saturated solution of (COD)$PtCl_2$ (1 equiv) in CD_2Cl_2 (0.2 mL) was added dropwise via syringe to a solution of P–P (1.05 equiv) in CD_2Cl_2 (0.5 mL) in an NMR tube, which was sealed under nitrogen. The reaction mixture was monitored by ^{31}P NMR spectroscopy to ~95% conversion. The resultant yellow solution was diluted with diethyl ether (20 mL) and the resulting precipitate was filtered. The white precipitate was washed with diethyl ether and recrystallized by layering a saturate dichloromethane solution with diethyl ether at –20 °C to give pure (P–P)$PtCl_2$.

[E(2,3)]PtCl₂. Complex [**E(2,3)**]$PtCl_2$ was isolated as a white solid in (81.0 mg, 68.6%) from reaction of **E(2,3)** (95.4 mg, 0.108 mmol) with (COD)$PtCl_2$ (38.4 mg, 0.103 mmol) employing

general procedure 2. ^{1}H NMR (500 MHz, CD_2Cl_2): δ 7.84 (qd, *J* = 13.4, 7.8 Hz, 4H), 7.63 (ddd, *J* = 31.8, 11.8, 7.4 Hz, 5H), 7.54 – 7.30 (m, 13H), 7.19 (dt, *J* = 17.0, 7.8 Hz, 4H), 7.00 (s, 1H), 6.86 (d, *J* = 8.5 Hz, 3H), 6.69 (ddq, *J* = 30.5, 21.8, 12.3, 9.9 Hz, 3H), 6.20 (d, *J* = 8.6 Hz, 1H), 5.48 (d, *J* = 8.4 Hz, 1H), 4.34 (dd, *J* = 12.2, 6.1 Hz, 2H), 4.04 (dt, *J* = 42.9, 10.6 Hz, 3H), 3.21 – 2.76 (m, 10H), 2.63 (q, *J* = 10.7 Hz, 1H), 2.40 (t, *J* = 10.9 Hz, 1H), 2.12 (td, *J* = 11.2, 6.0 Hz, 1H), 1.69 (dq, *J* = 13.7, 7.4 Hz, 1H), 1.42 (dt, *J* = 16.9, 9.4 Hz, 1H). $^{13}C\{^{1}H\}$ NMR (126 MHz, CD_2Cl_2): δ 158.09, 157.99, 157.17, 157.11, 157.01, 155.59, 146.68, 145.81, 144.09, 142.02, 135.77, 135.68, 135.58, 135.52, 135.44, 135.37, 135.29, 134.90, 132.27, 132.26, 131.95, 131.94, 131.05, 131.03, 130.99, 130.98, 129.29, 129.25, 129.19, 129.02, 128.82, 128.74, 128.51, 128.27, 128.18, 128.14, 128.05, 127.61, 127.58, 127.52, 127.49, 126.81, 126.12, 125.43, 125.36, 124.90, 124.83, 124.48, 124.44, 123.99, 123.94, 120.38, 118.90, 117.99, 113.80, 113.09, 109.34, 71.13, 68.47, 66.21, 63.60, 37.32, 36.07, 32.39, 32.14, 30.97. ^{31}P NMR (202 MHz, CD_2Cl_2): δ 9.44, 8.44 (ABq, J_{PP} = 18 Hz, J_{PtP} = 3670, 3662 Hz). HRMS (ESI$^+$) calcd (found) for $C_{59}H_{50}ClO_4P_2Pt$ $[M–Cl]^+$: 1115.2524 (1115.2508).

[E(3,3)]PtCl₂. Complex [**E(3,3)**]$PtCl_2$ was isolated as a colorless crystals (49.5 mg, 81.7%) from reaction of **E(3,3)** (48.9 mg, 0.054 mmol) with (COD)$PtCl_2$ (19.4 mg, 0.052 mmol) employing general procedure 2. ^{1}H NMR (500 MHz, CD_2Cl_2): δ 7.78 (s, br, 4H), 7.66 – 7.59 (m, 4H), 7.50 – 7.38 (m, 8H), 7.25 (t, *J* = 7.5 Hz, 4H), 7.18 (d, *J* = 8.1 Hz, 2H), 6.95 (s, 2H), 6.88 (td, *J* = 8.3, 2.3 Hz, 2H), 6.75 – 6.63 (m, 4H), 6.26 (d, *J* = 8.4 Hz, 2H), 4.31 – 4.19 (m, 4H), 3.38 – 3.28 (m, 2H), 3.17 – 2.80 (m, 10H), 1.65 – 1.49 (m, 4H). $^{13}C\{^{1}H\}$ NMR (126 MHz, CD_2Cl_2): δ 157.29, 157.25, 157.20, 155.78, 145.25, 141.61, 135.55, 135.52, 135.48, 135.07, 131.96, 131.04, 129.52, 129.49, 129.47, 129.42, 129.37, 129.13, 129.09, 128.97, 128.94, 128.53, 128.49, 128.26, 128.22, 128.17, 127.59, 127.54, 127.50, 126.87, 126.81, 126.75, 126.35, 125.23, 125.19, 125.16, 124.50, 124.45, 123.96, 123.91, 118.55, 113.33, 111.03, 66.10, 65.39, 36.48, 32.12, 27.76. ^{31}P NMR (162 MHz, CD_2Cl_2) δ 8.78 (s, J_{PtP} = 3664 Hz). HRMS (ESI$^+$) calcd (found) for $C_{60}H_{52}ClO_4P_2Pt$ $[M–Cl]^+$: 1129.2681 (1129.2685).

Attempted Synthesis of [E(2,2)]PtCl₂. A saturated solution of (COD)$PtCl_2$ (21.9 mg, 0.058 mmol) in CD_2Cl_2 (0.2 mL) was added portionwise (~0.25 equiv) via syringe to a solution of **E(2,2)** (53.8 mg, 0.062 mmol) in CD_2Cl_2 (0.4 mL) at room temperature and analyzed after each addition by ^{31}P NMR spectroscopy within 5 min of addition (Figure S1). After each addition, ^{31}P NMR analysis displayed platinum-coupled resonances for both [**E(2,2)**]$PtCl_2$ (δ 9.34) and [**Z(2,2)**]$PtCl_2$ (δ 8.10), with the [**E(2,2)**]$PtCl_2$:[**Z(2,2)**]$PtCl_2$ ratio decreasing after each addition with conversion of [**E(2,2)**]$PtCl_2$ to [**Z(2,2)**]$PtCl_2$ ultimately forming exclusively [**Z(2,2)**]$PtCl_2$ (Figure S1).

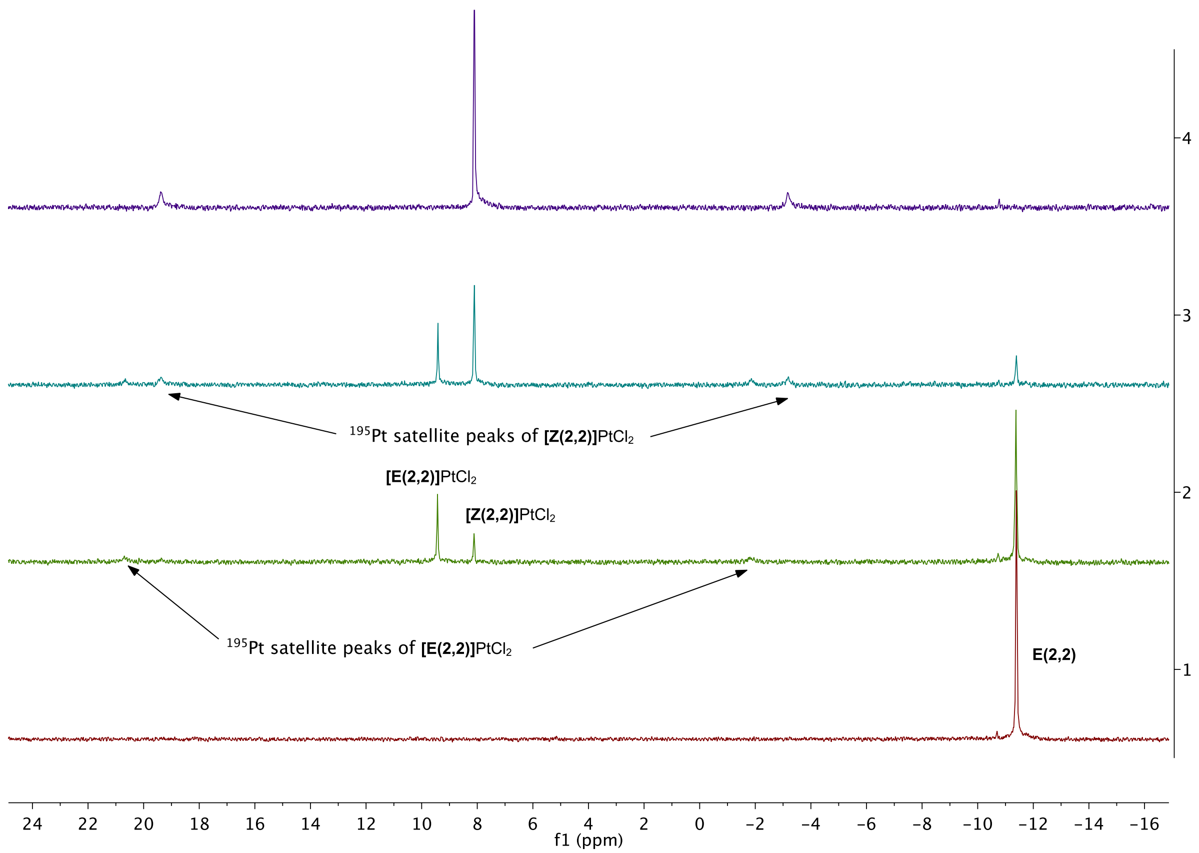

Figure S1. Stacked ^{31}P NMR spectra for sequential addition of portions of (COD)$PtCl_2$ (~0.25 equiv) to **E(2,2)** showing the initial formation of [**E(2,2)**]$PtCl_2$ and subsequent isomerization to [**Z(2,2)**]$PtCl_2$ at ambient temperature in CD_2Cl_2. (1) Spectrum of pure **E(2,2)** ligand. (2-4) Spectra recorded during sequential addition of portions of (COD)$PtCl_2$ (0.25 eq) to **E(2,2)**. Time interval between two consecutive spectra was ca. 5 min.

Synthesis of (P–P)PtAr2 complexes

General procedure for the synthesis of (P–P)PtAr$_2$ (Ar = 4-$C_6H_4NMe_2$) complexes. A solution of (P–P)PtCl_2 (1 eq) in diethyl ether (3 mL) was stirred at room temperature for 10 min, to which a solution of 4-(*N*,*N*-dimethyl)aniline magnesium bromide (0.5 M in THF, 2.3 eq) was added dropwise. The resulting solution was stirred for 4 h and treated with degassed saturated aqueous $NaHCO_3$ (3 mL). The layers were separated and the organic fraction was washed with water (3 × 15 mL) and dried (Na_2SO_4). The resulting light yellow solution was diluted with diethyl ether to achieve a total volume of ~30 mL, layered with pentane (30 mL), and cooled at –20 ° C overnight. The resulting suspension was filtered and the filtrate was concentrated under vacuum. The resultant white solid was dissolved in toluene and precipitated with pentane to give (P–P)PtAr$_2$.

(MeOBiphep)PtAr$_2$. Complex (MeOBiphep)PtAr$_2$ was isolated as a beige solid (56.0 mg, 31.1%) from (MeOBiphep)PtCl_2 (150 mg, 0.177 mmol) applying the general procedure. ^{1}H NMR (500 MHz, CD_2Cl_2): δ 7.90 – 7.83 (m, 4H), 7.30 (t, *J* = 7.5 Hz, 2H), 7.27 – 7.19 (m, 8H), 7.08 (t, *J* = 7.3 Hz, 2H), 7.02 (t, *J* = 7.5 Hz, 4H), 6.91 – 6.85 (m, 3H), 6.85 – 6.77 (m, 5H), 6.25 (d, *J* = 8.3 Hz, 2H), 6.06 (d, *J* = 7.9 Hz, 4H), 3.35 (s, 6H), 2.55 (s, 12H). ^{13}C{^{1}H} NMR (126 MHz, CD_2Cl_2) δ 157.87, 146.38, 138.24, 136.30, 136.25, 136.20, 135.40, 135.36, 135.32, 130.18, 128.71, 128.43, 128.39, 128.36, 127.90, 127.85, 127.81, 127.23, 127.19, 127.15, 126.96, 123.43, 123.40, 123.37, 114.15, 114.13, 114.10, 113.34, 111.18, 55.00, 42.01. ^{31}P NMR (202 MHz, CD_2Cl_2): δ 17.04 (s, J_{PtP} = 1766 Hz). HRMS (ESI$^+$) calcd (found) for $C_{54}H_{52}N_2O_2P_2Pt$ [M+2H]$^{2+}$: 509.6649 (509.6655).

[Z(2,2)]PtAr$_2$. Complex [**Z(2,2)**]PtAr$_2$ was isolated as a beige solid (20.0 mg, 21.0%) from [**Z(2,2)**]PtCl_2 (83.1 mg, 0.073 mmol) applying the general procedure. ^{1}H NMR (500 MHz, CD_2Cl_2): δ 7.80 (t, *J* = 8.7 Hz, 4H), 7.46 (d, *J* = 2.4 Hz, 2H), 7.26 (t, *J* = 7.4 Hz, 2H), 7.21 – 7.14 (m, 8H), 7.06 (d, *J* = 8.3 Hz, 2H), 7.02 – 6.92 (m, 6H), 6.79 – 6.72 (m, 6H), 6.60 (dd, *J* = 8.2, 2.1 Hz, 2H), 6.18 – 6.13 (m, 2H), 5.98 (d, *J* = 7.9 Hz, 4H), 3.95 (dt, *J* = 12.0, 6.4 Hz, 2H), 3.90 – 3.82 (m, 4H), 3.75 (dt, *J* = 10.7, 5.7 Hz, 2H), 2.85 – 2.64 (m, 8H), 2.47 (s, 12H). ^{13}C{^{1}H} NMR (126 MHz, CD_2Cl_2): δ 157.22, 156.92, 156.88, 156.85, 146.39, 141.84, 141.52, 138.23, 136.25, 136.20, 136.14, 135.89, 135.44, 135.40, 135.36, 135.03, 130.64, 130.23, 128.74, 128.51, 128.48, 128.44, 128.01, 127.97, 127.93, 127.69, 127.63, 127.57, 127.23, 127.19, 127.15, 126.94, 126.11, 123.69, 123.67, 123.64, 114.89, 114.10, 114.08, 114.05, 113.33, 111.54, 109.25, 66.48, 66.20, 53.84, 41.98, 40.88, 35.47, 30.09. ^{31}P NMR (202 MHz, CD_2Cl_2): δ 16.98 (J_{PtP} = 1765 Hz).

[Z(3,3)]PtAr$_2$. Complex [**Z(3,3)**]PtAr$_2$ was isolated as a beige solid (36.6 mg, 38.6%) from [**Z(3,3)**]PtCl_2 (83.1 mg, 0.073 mmol) applying the general procedure. ^{1}H NMR (500 MHz, CD_2Cl_2) δ 7.95 (s, br, 4H), 7.55 (d, *J* = 2.5 Hz, 2H), 7.33 – 7.23 (m, 6H), 7.20 (t, *J* = 7.5 Hz, 4H), 7.16 –

7.02 (m, 8H), 6.93 – 6.73 (m, 8H), 6.56 (dd, J = 8.5, 2.4 Hz, 2H), 6.15 (d, J = 8.2 Hz, 2H), 6.04 (d, J = 7.9 Hz, 4H) 3.71 (dt, J = 12.1, 6.1 Hz, 2H), 3.58 (dt, J = 10.6, 6.9 Hz, 2H), 3.48 – 3.38 (m, 4H), 3.02 – 2.90 (m, 2H), 2.89 – 2.76 (m, 6H), 2.55 (s, 12H), 1.87 – 1.71 (m, 4H). $^{13}C\{^{1}H\}$ NMR (126 MHz, CD_2Cl_2) δ 156.76, 156.72, 156.68, 156.49, 153.96, 153.88, 153.04, 152.95, 146.33, 141.86, 140.74, 138.29, 136.56, 136.51, 136.46, 135.67, 135.37, 135.34, 135.30, 134.94, 134.92, 134.57, 134.55, 130.30, 130.27, 129.89, 129.85, 129.51, 129.47, 128.37, 128.26, 128.19, 128.16, 128.13, 127.99, 127.64, 127.60, 127.56, 127.17, 127.13, 127.09, 126.04, 123.10, 123.07, 123.05, 114.13, 110.57, 110.08, 64.50, 63.01, 42.02, 35.12, 30.20, 28.62. ^{31}P NMR (202 MHz, CD_2Cl_2) δ 17.14 (J_{PtP} = 1771 Hz). HRMS (ESI$^+$) calcd (found) for $C_{76}H_{72}N_2O_4P_2Pt$ $[M+2H]^{2+}$: 668.2381 (668.2394).

[E(2,3)]PtAr₂. Complex [**E(2,3)**]$PtAr_2$ was isolated (33.5 mg, 34.4%) as a beige solid from [**E(2,3)**]$PtCl_2$ (84.8 mg, 0.074 mmol) applying the general procedure. ^{1}H NMR (500 MHz, CD_2Cl_2): δ 7.92 – 7.86 (m, 2H), 7.86 – 7.80 (m, 2H), 7.21 – 6.83 (m, 27H), 6.72 – 6.62 (m, 4H), 6.07 (d, J = 7.3 Hz, 1H), 6.02 (t, J = 6.8 Hz, 3H), 5.42 (d, J = 8.1 Hz, 1H), 4.34 – 4.20 (m, 2H), 4.00 – 3.85 (m, 3H), 3.33 (q, J = 8.8 Hz, 1H), 3.15 – 2.99 (m, 4H), 2.90 – 2.76 (m, 5H), 2.55 (d, J = 3.3 Hz, 12H), 2.26 – 2.16 (m, 1H), 1.91 (td, J = 10.9, 5.7 Hz, 1H), 1.59 – 1.42 (m, 1H). $^{13}C\{^{1}H\}$ NMR (126 MHz, CD_2Cl_2): δ 158.17, 158.09, 157.33, 157.19, 157.10, 155.71, 146.73, 146.29, 146.18, 145.79, 144.19, 141.74, 137.35, 137.19, 136.48, 136.37, 136.34, 136.27, 135.69, 135.33, 135.02, 134.99, 134.91, 134.77, 134.69, 130.51, 130.23, 130.21, 128.68, 128.67, 128.11, 128.09, 128.04, 128.02, 127.94, 127.86, 127.55, 127.37, 127.31, 127.30, 127.24, 127.14, 127.11, 126.95, 126.85, 126.24, 126.18, 125.97, 123.58, 123.52, 123.01, 122.95, 120.41, 118.84, 118.57, 114.28, 114.22, 114.16, 113.33, 111.95, 111.33, 109.17, 71.64, 67.92, 66.19, 63.39, 42.06, 42.02, 40.89, 37.25, 35.94, 32.43, 32.11, 30.88, 30.20, 30.18, 29.98. ^{31}P NMR (202 MHz, CD_2Cl_2): δ 16.84 (J_{PP} = 14 Hz, J_{PtP} = 1770 Hz), 15.62 (J_{PP} = 16 Hz, J_{PtP} = 1755 Hz).

[E(3,3)]PtAr₂. Complex [**E(3,3)**]$PtAr_2$ was isolated (13.7 mg, 24.2%) as a beige solid from [**E(3,3)**]$PtCl_2$ (9.5 mg, 0.042 mmol) applying the general procedure. ^{1}H NMR (500 MHz, CD_2Cl_2) δ 7.80 (t, J = 8.8 Hz, 4H), 7.32 – 7.22 (m, 4H), 7.21 – 7.12 (m, 10H), 7.10 (d, J = 7.3 Hz, 2H), 7.03 (t, J = 7.6 Hz, 4H), 6.93 (d, J = 2.3 Hz, 2H), 6.91 – 6.84 (m, 4H), 6.75 (dd, J = 7.7, 4.7 Hz, 2H), 6.68 (d, J = 7.8 Hz, 2H), 6.17 – 6.09 (m, 2H), 6.05 (d, J = 8.0 Hz, 4H), 4.30 – 4.12 (m, 4H), 3.23 (td, J = 10.5, 5.6 Hz, 2H), 3.09 – 2.77 (m, 8H), 2.55 (s, 12H), 1.55 – 1.39 (m, 2H). $^{13}C\{^{1}H\}$ NMR (126 MHz, CD_2Cl_2) δ 157.32, 157.28, 157.24, 155.88, 146.33, 145.21, 141.58, 138.38, 137.50, 136.14, 136.09, 136.04, 135.08, 135.02, 134.98, 134.94, 130.15, 128.73, 128.30, 128.26, 128.22, 127.74, 127.70, 127.66, 127.36, 127.32, 127.29, 126.96, 126.37, 123.50, 123.47, 123.45, 118.61, 114.22, 114.20, 114.18, 113.34, 111.41, 111.26, 65.63, 53.84, 40.89, 36.42, 32.12, 27.79. ^{31}P

NMR (202 MHz, CD_2Cl_2) δ 16.30 (s, J_{PtP} = 1765 Hz). HRMS (ESI$^+$) calcd (found) for $C_{76}H_{72}N_2O_4P_2Pt$ $[M+2H]^{2+}$: 668.2381 (668.2391).

Table S1. ^{31}P NMR chemical shifts and $^1J_{Pt\text{-}P}$ for platinum bisphosphine complexes.

		$(P–P)PtCl_2$		$(P–P)PtAr_2$	
entry	(P–P)	δ ^{31}P	$^1J_{Pt\text{-}P}$ (Hz)	δ ^{31}P	$^1J_{Pt\text{-}P}$ (Hz)
1	MeOBiphep	8.01	3646	17.04	1766
2	Z(2,2)	8.10	3652	16.98	1765
3	Z(3,3)	7.81	3655	17.14	1771
4	E(2,2)	9.34	3646	—	—
5	E(2,3)	9.44	3670	16.70	1770
		8.44	3662	15.62	1755
6	E(3,3)	8.78	3664	16.30	1765

Table S2. ^{31}P NMR chemical shifts, $^1J_{PtP}$, and P–Pt–P bond angles for structurally characterized complexes of the form $[Ph_2P(CH_2)_xPPh_2]PtCl_2$.

x	δ_P[a]	$^1J_{PtP}$ (Hz)	P–Pt–P (°)[b]
5	9.4	3643	103.73
4	10.8	3544	95.37
3	–5.5	3409	91.63

[a]In CH_2Cl_2 / $CDCl_3$. [b]Data taken from reference.[S4]

Kinetic analysis of reductive elimination

An NMR tube containing a solution of (P–P)$PtAr_2$ (9.8 × 10^{-3} mmol, 16.3 mM), dibenzylideneacetone (2.30 mg, 9.8 × 10^{-3} mmol, 16.3 mM), and trimethylphosphine oxide (0.60 mg, 6.5 × 10^{-3} mmol, 10.8 mM) in toluene-d_8 (0.60 mL) was placed into the probe of an NMR spectrometer preheated at 85 °C and analyzed in 10 min intervals by ^{1}H NMR spectroscopy through three half-lives. The concentration of (P–P)$PtAr_2$ was determined by integrating the dimethyamino resonance of (P-P)$PtAr_2$ at δ 2.46 versus the methyl resonance of trimethylphosphine oxide at δ 0.85. Reductive elimination gives 77 – 95% yield of the Ar-Ar product determined by ^{1}H NMR. First-order rate constants were obtained from linear plots of ln[(P–P)$PtAr_2$] versus time (Figures S2-S6; Table S3). Owing to the low forces and modest changes in rate of reductive elimination to the compressive ligands, the reductive elimination of [Z(2,3)]$PtAr_2$ was not investigated.

Table S3. First order rate constants for the reductive elimination of (P–P)$PtAr_2$ (16.3 mM) in toluene-d_8 containing DBA (16.3 mM) at 85 °C.

entry	(P–P)	Restoring force (pN)	$(10^5)k$ (s^{-1})
1	MeOBiphep	0	6.92 ± 0.05
2	Z(2,2)	-65	5.32 ± 0.03
3	Z(2,2)	-65	6.07 ± 0.06
4	Z(2,2)	-65	5.87 ± 0.07
5	Z(3,3)	-3	6.12 ± 0.05
6	Z(3,3)	-3	5.67 ± 0.02
7	E(2,3)	228	18.7 ± 0.3
8	E(2,3)	228	20.2 ± 0.5
9	E(3,3)	130	14.8 ± 0.2
10	E(3,3)	130	13.9 ± 0.4

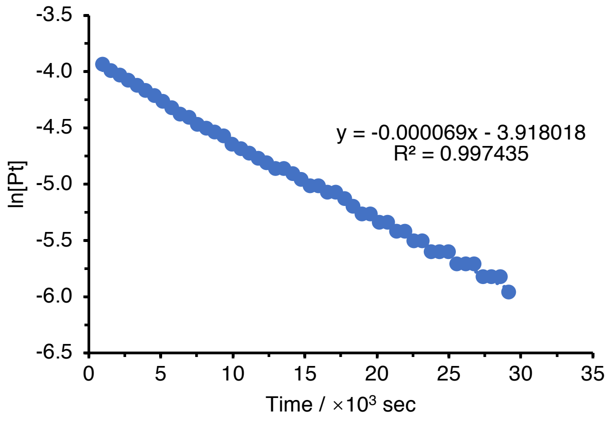

Figure S2. First order rate plot for the reductive elimination of (MeOBiphep)$PtAr_2$ (16.3 mM) in toluene-d_8 containing DBA (16.3 mM) at 85 °C.

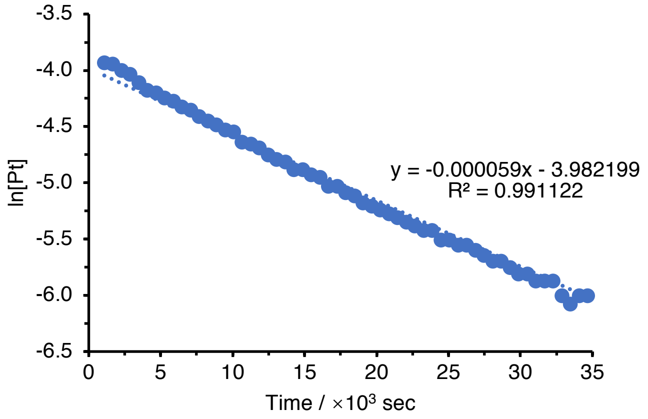

Figure S3. First order rate plot for the reductive elimination of [**Z(2,2)**]$PtAr_2$ (16.3 mM) in toluene-d_8 containing DBA (16.3 mM) at 85 °C.

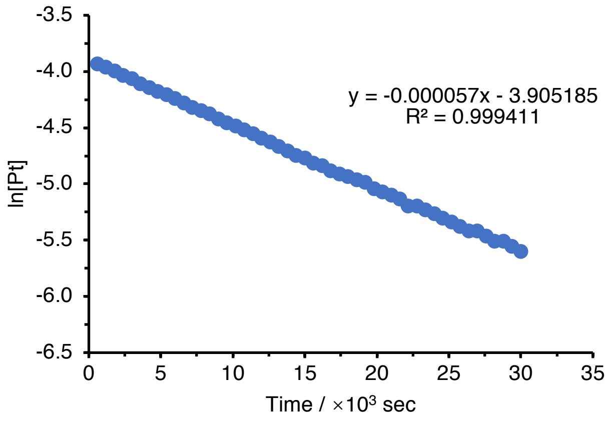

Figure S4. First order rate plot for the reductive elimination of [**Z(3,3)**]$PtAr_2$ (16.3 mM) in toluene-d_8 containing DBA (16.3 mM) at 85 °C.

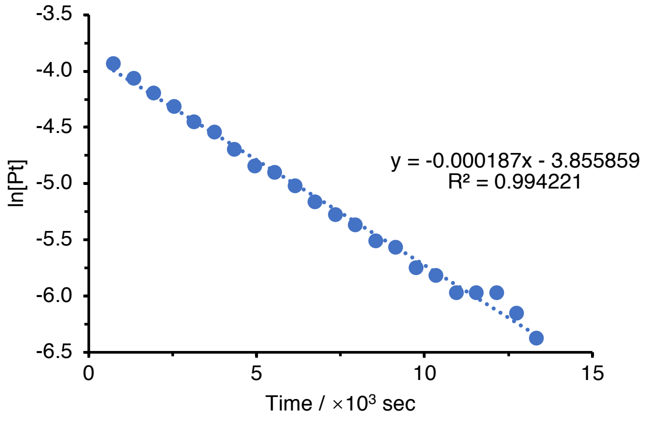

Figure S5. First order rate plot for the reductive elimination of [**E(2,3)**]$PtAr_2$ (16.3 mM) in toluene-d_8 containing DBA (16.3 mM) at 85 °C.

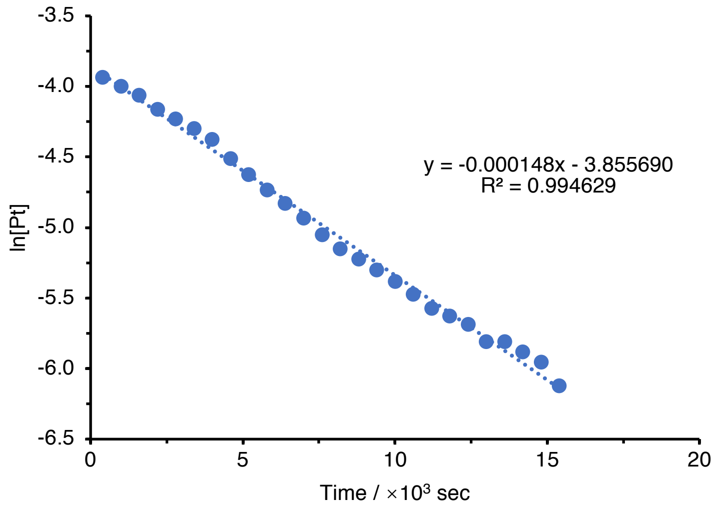

Figure S6. First order rate plot for the reductive elimination of [**E(3,3)**]$PtAr_2$ (16.3 mM) in toluene-d_8 containing DBA (16.3 mM) at 85 °C.

Computed O···O distances and restoring forces for force probe ligands

All calculations were performed in the gas phase with Gaussian09.[S5, S6] All conformations of the ligand found with VeraChem conformational search were then optimized at the B3LYP/6-31+G(d) level using the Berny algorithm, followed by reoptimization of all unique conformers within 1.2 kcal/mol of the global conformational minimum (for each diastereomer for E ligands) at B3LYP/6-311+G(d) level. All metal complexes were optimized at B3LYP/def2SVP. Because of the size of the molecules, frequencies were not calculated and ensemble-averaging of the structural parameters was performed using electronic energies only. The latter is equivalent to assuming that thermodynamic corrections are identical for all conformers of the same conformational state.

Table S4. Force dependent geometrical parameters of MeOBiphep.

Restoring force (pN)	P···P distance (Å)	O···O distance (Å)	(P)C-C-C-C(P) torsion of biaryl (°)
-200	3.680	2.902	76
-175	3.697	2.955	77
-150	3.717	3.009	78
-125	3.738	3.063	79
-100	3.761	3.117	80
-75	3.786	3.171	81
-50	3.813	3.225	82
-25	3.842	3.280	83
0	3.858	3.330	84
25	3.904	3.389	86
50	3.939	3.444	87
75	3.974	3.499	88
100	4.012	3.555	90
125	4.052	3.610	91
150	4.093	3.666	92
175	4.136	3.721	94
200	4.181	3.777	95
225	4.228	3.833	97
250	4.277	3.890	98
275	4.327	3.946	100
300	4.380	4.002	102
325	4.434	4.059	103
350	4.490	4.116	105
375	4.547	4.173	107
400	4.607	4.230	109
425	4.668	4.287	111
450	4.732	4.345	112
475	4.797	4.402	114
500	4.864	4.460	116

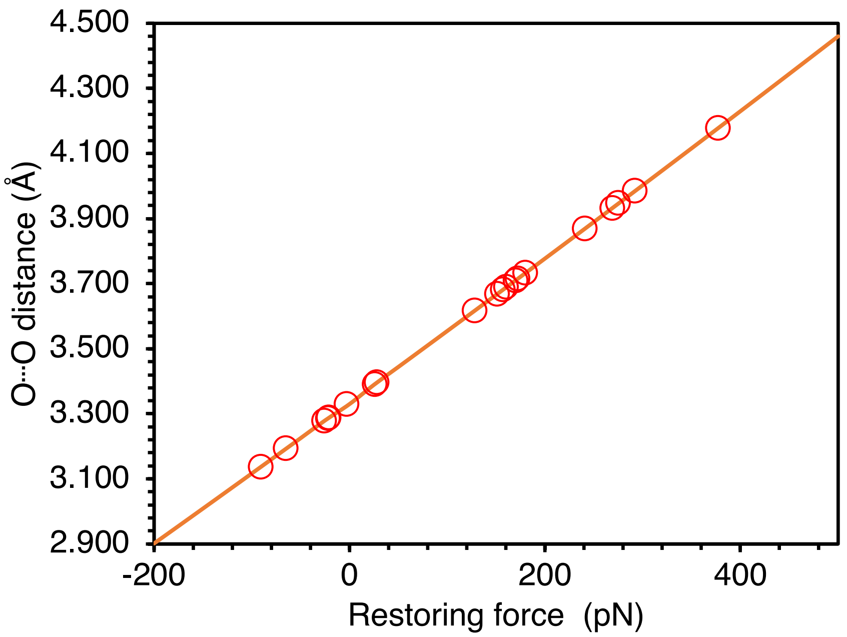

Figure S7. Calculated $_{biphen}O \cdots O_{biphen}$ distance of force probe ligands (circles) plotted on MeOBiphep calibration curve (solid curve, data from Table S4) yields restoring force values for individual conformers.[S1]

Table S5. Force dependent ensemble average $_{biphen}O \cdots O_{biphen}$ distances and ensemble-averaged forces of force probe ligands.

entry	(P-P)	distance (Å)	Restoring force (pN)
1	Z(2,2)	3.19	-65
2	Z(2,3)	3.33	-6
3	Z(3,3)	3.36	-3
4	MeOBiphep	3.77	0
5	E(3,3)	3.99	130
6	E(2,3)	3.97	228
7	E(2,2)	4.22	241

Computed structure-reactivity relationships of reductive elimination

All calculations were performed with aforementioned method. Energy of optimized reactants and transition states are calculated for (P-P)$PtPh_2$ complexes with diverse ligands under different restoring forces. Force-dependent activation free energies of reductive elimination from (P-P)$PtPh_2$ complexes vs restoring force is plotted in Figure 2 and coordinates of optimized conformations for each complex are listed below.

Table S6. Minimal energy conformation for **1a**.

Atomic number	Coordinates (Å)		
	X	Y	Z
78	0.305404	-2.38292	0.437471
6	0.209932	1.767205	0.710405
6	1.286806	1.010262	1.222194
6	1.852185	1.35659	2.464291
1	2.69099	0.788086	2.861331
6	1.355512	2.429645	3.197414
1	1.808121	2.685618	4.158889
6	0.285142	3.18277	2.716408
6	-0.28007	2.856225	1.479024
6	-0.41205	1.571183	-0.6424
6	-1.30821	0.53226	-0.97695
6	-1.906	0.512777	-2.25155
1	-2.60843	-0.27581	-2.51453
6	-1.61571	1.499533	-3.18841
1	-2.09051	1.469759	-4.17253
6	-0.72454	2.527583	-2.88316
6	-0.13113	2.565375	-1.61683
6	3.499184	-0.88974	1.171988
6	3.470434	-1.54804	2.41495
1	2.513437	-1.84164	2.849603
6	4.653472	-1.8493	3.09119
1	4.608514	-2.36445	4.053805
6	5.889099	-1.5173	2.527155
1	6.816488	-1.76401	3.050499
6	5.930437	-0.88444	1.283416
1	6.891229	-0.63429	0.825911
6	4.744918	-0.57194	0.609553
1	4.803827	-0.08787	-0.36526
6	2.350353	0.053022	-1.33535

6	2.057308	-0.76345	-2.43928
1	1.506854	-1.6945	-2.28294
6	2.450731	-0.38524	-3.72667
1	2.214764	-1.03032	-4.57669
6	3.141164	0.813305	-3.92431
1	3.453658	1.107013	-4.92993
6	3.427513	1.639024	-2.83134
1	3.964646	2.579548	-2.98127
6	-3.10252	-1.72082	-0.51765
6	-2.91732	-2.58887	-1.60912
1	-1.91198	-2.76519	-1.99543
6	-4.00238	-3.24485	-2.19216
1	-3.8357	-3.91783	-3.03675
6	-5.29099	-3.06195	-1.68111
1	-6.13963	-3.58505	-2.12942
6	-5.48352	-2.22054	-0.58382
1	-6.48433	-2.08238	-0.16634
6	-4.39819	-1.55377	-0.00528
1	-4.57264	-0.91096	0.857568
6	-2.21643	-0.09787	1.736129
6	-3.12882	0.971605	1.71547
1	-3.45134	1.395758	0.762355
6	-3.61744	1.505916	2.910258
1	-4.33424	2.331124	2.882041
6	-3.19346	0.987312	4.13895
1	-3.57783	1.406241	5.072728
6	-2.27403	-0.06439	4.168378
1	-1.9307	-0.46782	5.124254
6	-1.78792	-0.60408	2.973548
1	-1.05963	-1.41861	2.993338
6	3.030582	1.26545	-1.54498
1	3.242162	1.925795	-0.70148
15	1.876063	-0.52953	0.352052
15	-1.60061	-0.89422	0.187446
8	0.743209	3.532469	-1.23841
8	-1.3208	3.539675	0.938053
6	1.038996	4.590709	-2.11943
1	1.734705	5.25371	-1.58723
1	1.524878	4.232057	-3.04429
6	-1.83567	4.669682	1.602573
1	-2.25837	4.409354	2.589294

1	-1.0672	5.451811	1.738304
1	-0.50025	3.289714	-3.62863
1	-0.09912	4.015713	3.304131
1	0.134468	5.164352	-2.39045
1	-2.63848	5.066203	0.966103
6	1.833737	-3.77212	0.640864
6	1.950767	-4.56343	1.801804
1	1.217297	-4.45672	2.60564
6	2.784122	-3.99255	-0.37492
1	2.744232	-3.41252	-1.30116
6	3.804308	-4.94323	-0.23758
1	4.523219	-5.08568	-1.05055
6	3.909359	-5.70239	0.930089
1	4.706068	-6.44326	1.041273
6	2.974401	-5.50534	1.951178
1	3.032665	-6.10002	2.8683
6	-0.91743	-4.0566	0.540235
6	-1.82635	-4.25482	1.597703
1	-1.91895	-3.50786	2.391108
6	-0.8545	-5.06773	-0.44026
1	-0.14041	-4.9797	-1.26358
6	-1.67102	-6.20227	-0.38186
1	-1.59364	-6.96517	-1.16298
6	-2.5696	-6.37389	0.675803
1	-3.20376	-7.26336	0.727683
6	-2.63828	-5.39458	1.669312
1	-3.33165	-5.5123	2.508018

Table S7. Transition state for the reductive elimination of **1a**

Atomic number	Coordinates (Å)		
	X	Y	Z
78	0.225408	-2.33128	0.192856
6	0.070909	1.801159	0.645557
6	1.121493	1.082433	1.260302
6	1.635249	1.525873	2.494409
1	2.448075	0.982915	2.974243
6	1.121262	2.662591	3.111064
1	1.534253	2.995661	4.066852
6	0.082724	3.383519	2.520724
6	-0.43836	2.953478	1.295762

6	-0.48737	1.475993	-0.70913
6	-1.3449	0.388856	-0.98453
6	-1.7726	0.156406	-2.3088
1	-2.39354	-0.71026	-2.53611
6	-1.4009	1.015538	-3.33579
1	-1.7408	0.822806	-4.35666
6	-0.5933	2.125019	-3.07586
6	-0.14005	2.352506	-1.77245
6	3.115967	-0.99172	1.666894
6	2.740216	-1.72334	2.808668
1	1.685839	-1.96427	2.967069
6	3.695585	-2.14565	3.735033
1	3.382731	-2.70563	4.620337
6	5.050002	-1.86614	3.522862
1	5.800802	-2.20554	4.241216
6	5.43676	-1.15825	2.382461
1	6.493333	-0.94167	2.203748
6	4.477819	-0.71985	1.462546
1	4.7989	-0.16408	0.58019
6	2.745156	0.150718	-0.96331
6	3.043456	-0.74453	-2.00406
1	2.680482	-1.77237	-1.95381
6	3.809847	-0.33108	-3.09736
1	4.035528	-1.0414	-3.89692
6	4.284412	0.981816	-3.16776
1	4.884261	1.304786	-4.02293
6	3.989657	1.880101	-2.13689
1	4.36161	2.907471	-2.18214
6	-3.3842	-1.57606	-0.18488
6	-3.67203	-2.90346	0.169375
1	-2.89643	-3.52734	0.614683
6	-4.94795	-3.43679	-0.04303
1	-5.14767	-4.47361	0.238254
6	-5.95122	-2.65389	-0.61707
1	-6.94667	-3.07224	-0.78825
6	-5.67554	-1.32884	-0.97303
1	-6.45581	-0.70706	-1.42046
6	-4.40503	-0.79194	-0.75489
1	-4.21155	0.244701	-1.0371
6	-2.16164	-0.09278	1.860805
6	-3.07005	0.97741	1.925265

1	-3.45247	1.427774	1.007561
6	-3.48029	1.482908	3.160023
1	-4.18995	2.314126	3.196791
6	-2.99429	0.923881	4.348024
1	-3.32211	1.317469	5.313854
6	-2.09147	-0.13987	4.29335
1	-1.70728	-0.58242	5.216085
6	-1.67755	-0.64485	3.055856
1	-0.97583	-1.48144	3.008903
6	3.224875	1.469619	-1.04075
1	2.996366	2.182224	-0.24674
15	1.781643	-0.4804	0.484801
15	-1.70346	-0.92588	0.266837
8	0.657031	3.400042	-1.4373
8	-1.45889	3.584289	0.659059
6	1.005785	4.351957	-2.41403
1	1.613921	5.112333	-1.90508
1	1.603286	3.90611	-3.22913
6	-1.94822	4.803511	1.1645
1	-2.40787	4.683483	2.162184
1	-1.15397	5.56894	1.229457
1	-0.31186	2.791954	-3.89003
1	-0.3162	4.266154	3.019746
1	0.114512	4.839183	-2.849
1	-2.71811	5.147371	0.460214
6	1.440471	-4.04692	-0.28781
6	2.437497	-4.50475	0.615133
1	2.243226	-4.47876	1.688849
6	1.7581	-4.14758	-1.6723
1	1.02125	-3.83332	-2.41506
6	2.984672	-4.64212	-2.11321
1	3.185668	-4.69795	-3.1874
6	3.953253	-5.06872	-1.19669
1	4.911534	-5.46417	-1.54221
6	3.663505	-4.99403	0.172268
1	4.404659	-5.32277	0.906504
6	-0.28124	-4.41973	0.148515
6	-0.50118	-4.95099	1.452213
1	0.083972	-4.56828	2.291789
6	-1.06511	-4.98917	-0.89386
1	-0.93362	-4.6357	-1.9185

6	-1.99159	-5.99977	-0.65222
1	-2.56891	-6.40597	-1.48804
6	-2.19456	-6.49351	0.644051
1	-2.91656	-7.2923	0.83042
6	-1.44126	-5.95154	1.693414
1	-1.58194	-6.31928	2.714238

Table S8. Minimal energy conformation for **2a**

Atomic number	Coordinates (Å)		
	X	Y	Z
6	0.341484	15.23817	5.164782
6	1.104654	10.29102	8.715032
6	-0.04412	10.33083	2.151546
6	0.409949	13.90338	4.729042
6	1.540568	11.83187	6.401027
6	0.535584	7.933415	3.438889
6	1.192786	10.21677	2.818788
6	1.974162	9.832715	7.720647
6	1.461584	8.984989	3.446695
6	2.184153	10.594	6.566902
6	3.268424	16.47143	-0.14022
6	3.33162	15.76788	1.066902
6	3.995878	16.03277	-1.25004
6	3.308201	13.98181	5.389762
6	2.619746	11.51256	-0.22529
6	4.132681	14.61846	1.187602
6	3.153475	11.00123	0.973852
6	3.747261	14.7719	4.122461
6	3.001146	11.00412	-1.47402
6	4.781122	14.88032	-1.14715
6	4.846601	14.17644	0.05777
6	4.456211	13.23099	6.106884
6	4.403335	13.34117	7.628029
6	4.082174	9.94837	0.864759
6	3.934754	9.966541	-1.55953
6	4.343386	16.9701	5.335166
6	4.790242	15.87611	4.366486
6	5.537031	12.61694	9.558149
6	4.473353	9.440128	-0.38089
6	5.93665	13.1161	2.896388

6	5.032787	19.05575	6.182661
6	6.248765	11.81985	3.33925
6	6.987497	14.01023	2.619195
6	7.579472	11.43037	3.5212
6	8.316924	13.61923	2.799288
6	8.615492	12.32969	3.253891
6	-0.72969	13.33299	4.130557
6	-0.69558	8.080615	2.793101
6	0.6599	12.2801	7.401663
6	-0.97798	9.286032	2.142856
6	-0.83261	15.98372	5.0043
6	0.444231	11.51379	8.550546
6	-1.90408	14.07481	3.981411
6	-1.95888	15.40413	4.414327
1	-0.69582	12.299	3.775743
1	1.200015	15.71908	5.640091
1	2.643222	17.36706	-0.21012
1	0.782727	6.989581	3.938494
1	2.738835	16.12993	1.910622
1	2.482805	8.87067	7.834857
1	1.885845	12.32529	-0.19268
1	3.945005	16.58325	-2.19459
1	2.882246	14.70928	6.101299
1	2.845926	10.21966	5.782339
1	2.424475	8.823884	3.944175
1	2.833817	15.2619	3.739569
1	2.561869	11.42341	-2.38649
1	3.444519	12.94334	8.020825
1	4.474899	12.16092	5.838679
1	3.346194	17.36844	5.035001
1	5.342113	14.51632	-2.01324
1	4.630567	12.14896	10.00052
1	5.453685	13.2695	0.113477
1	4.507425	9.498346	1.768478
1	6.420835	12.02963	9.858386
1	5.431736	13.628	5.783449
1	4.070546	19.55716	5.937377
1	4.234276	16.57214	6.368253
1	4.23397	9.566496	-2.53418
1	5.017027	16.37429	3.408021
1	4.459005	14.41289	7.93167

1	5.74326	15.45616	4.727753
1	5.437491	11.11249	3.53373
1	6.771394	15.01521	2.247977
1	5.198697	8.619759	-0.42812
1	5.633983	13.64271	9.978285
1	4.985972	18.72351	7.243201
1	5.848965	19.78976	6.078892
1	7.805606	10.41671	3.865623
1	9.123789	14.3255	2.580038
1	9.657526	12.02452	3.392068
1	0.93206	9.690317	9.613603
1	-0.28391	11.24963	1.604573
1	-1.42129	7.260556	2.784951
1	0.132445	13.23062	7.28373
1	-0.86422	17.02272	5.347487
1	-2.77925	13.6105	3.516605
1	-0.24694	11.87271	9.319737
1	-1.92877	9.412609	1.61219
1	-2.87675	15.98732	4.290572
8	5.489747	12.62668	8.158406
8	5.305675	17.99249	5.312268
15	1.898868	12.81485	4.882182
15	4.158807	13.57457	2.706955
78	2.569536	11.7797	2.808864

Table S9. Transition state for the reductive elimination of **2a**

Atomic number	Coordinates (Å)		
	X	Y	Z
6	0.301504	15.18159	5.167143
6	1.053155	10.15146	8.611194
6	0.100982	10.40459	1.686972
6	0.38275	13.8531	4.714513
6	1.51924	11.7648	6.350542
6	0.308759	8.095938	3.231869
6	1.38139	10.05991	2.205022
6	1.86768	9.687183	7.573817
6	1.441092	8.866457	2.976647
6	2.090444	10.48396	6.446258
6	3.221654	16.52217	-0.13735
6	3.350116	15.83718	1.076228

6	3.771655	15.99439	-1.3082
6	3.291037	13.92522	5.353468
6	2.511633	10.91163	-0.17263
6	4.036932	14.61162	1.142188
6	2.803779	10.41041	1.126848
6	3.733798	14.73142	4.093113
6	3.264906	10.54925	-1.2872
6	4.445789	14.76864	-1.25866
6	4.571712	14.08151	-0.05012
6	4.437931	13.15121	6.047585
6	4.405107	13.23775	7.570501
6	3.899679	9.506402	1.216206
6	4.346098	9.66488	-1.16615
6	4.341744	16.90052	5.356461
6	4.780079	15.82732	4.36096
6	5.55449	12.47486	9.47606
6	4.654516	9.149937	0.099925
6	5.956709	13.19622	2.773774
6	5.036102	18.96764	6.243678
6	6.348148	11.93158	3.245494
6	6.953461	14.1175	2.401693
6	7.701893	11.60163	3.365452
6	8.306078	13.78559	2.517123
6	8.68356	12.52849	3.003114
6	-0.74814	13.28519	4.09543
6	-0.94227	8.469369	2.721881
6	0.692142	12.21811	7.394531
6	-1.03089	9.632163	1.945193
6	-0.8769	15.92094	5.008805
6	0.460775	11.41626	8.515627
6	-1.9274	14.01949	3.949871
6	-1.99529	15.34182	4.403225
1	-0.69943	12.25868	3.718249
1	1.154928	15.66007	5.6547
1	2.687294	17.47726	-0.16223
1	0.402952	7.189276	3.839663
1	2.901769	16.27753	1.969982
1	2.322457	8.693571	7.63456
1	1.675678	11.6032	-0.30834
1	3.670961	16.53172	-2.25633
1	2.879005	14.64913	6.077192

1	2.702826	10.10717	5.621812
1	2.401451	8.534243	3.381489
1	2.820636	15.22896	3.718353
1	3.005113	10.96618	-2.26655
1	3.448234	12.84099	7.96898
1	4.437405	12.0848	5.762493
1	3.340385	17.30281	5.07561
1	4.871284	14.33704	-2.17009
1	4.64918	12.00739	9.921722
1	5.086136	13.11618	-0.03534
1	4.163995	9.074112	2.185768
1	6.436822	11.87632	9.758378
1	5.415409	13.5398	5.71894
1	4.06881	19.47144	6.023647
1	4.244799	16.48234	6.382524
1	4.931378	9.374868	-2.04412
1	5.008468	16.3478	3.414951
1	4.473651	14.30367	7.891192
1	5.733316	15.39494	4.707198
1	5.579019	11.19998	3.510348
1	6.674668	15.09878	2.008749
1	5.495364	8.457891	0.219724
1	5.663885	13.49336	9.911001
1	5.004128	18.61022	7.296693
1	5.84819	19.70736	6.147287
1	7.988864	10.61251	3.735532
1	9.069525	14.51344	2.224467
1	9.743546	12.27002	3.091789
1	0.869682	9.524024	9.489186
1	-0.00543	11.30044	1.068551
1	-1.829	7.858398	2.916052
1	0.219594	13.20255	7.331436
1	-0.91892	16.95486	5.36632
1	-2.79587	13.55732	3.470212
1	-0.18763	11.7807	9.318943
1	-1.99681	9.940424	1.529451
1	-2.91648	15.92022	4.281348
8	5.492295	12.50613	8.077791
8	5.301037	17.92702	5.344846
15	1.875267	12.76655	4.840177
15	4.147301	13.55951	2.651965

78	2.585935	11.75461	2.798678

Table S10. Minimal energy conformation for **4**

Atomic number	Coordinates (Å)		
	X	Y	Z
6	7.529718	9.653392	9.707915
6	8.745291	9.295002	10.3864
6	9.15875	11.26472	9.215959
6	7.800545	10.8655	8.98103
6	8.282803	8.578854	6.591676
6	9.440766	9.384273	6.315231
6	10.57645	8.829198	6.994476
6	10.10687	7.677015	7.710327
6	8.713411	7.516812	7.463089
6	5.017619	9.717366	11.06388
6	3.956279	9.093373	11.74401
6	3.255387	9.772918	12.74314
6	3.593068	11.0893	13.07331
6	4.639923	11.72147	12.39732
6	5.348535	11.04078	11.40107
6	6.268697	7.175474	10.50518
6	6.671899	7.073972	11.84893
6	6.954434	5.826648	12.41287
6	6.830777	4.662786	11.64498
6	6.416453	4.751342	10.31303
6	6.133377	6.00021	9.748215
6	6.561747	10.47744	5.25263
6	7.310373	10.788	4.103064
6	7.300827	12.08341	3.577907
6	6.537274	13.08515	4.188968
6	5.778524	12.78369	5.323968
6	5.788024	11.48633	5.84953
6	6.557323	7.688783	4.497494
6	7.434392	6.597028	4.38119
6	7.38767	5.760815	3.260313
6	6.466889	6.004946	2.23798
6	5.588901	7.088164	2.345854
6	5.628096	7.920305	3.467217
26	9.021829	9.343269	8.340754
1	8.896955	8.408985	11.00047

1	7.082174	11.39842	8.360609
1	9.451725	10.27791	5.694563
1	10.70413	7.041795	8.36378
1	8.07853	6.740218	7.887517
1	3.665047	8.071445	11.49143
1	2.431462	9.26877	13.25713
1	3.040263	11.62116	13.85424
1	4.915804	12.75093	12.64737
1	6.171255	11.55117	10.89552
1	6.754566	7.974112	12.46452
1	7.266395	5.7628	13.46018
1	7.047362	3.686349	12.08976
1	6.301943	3.844782	9.710705
1	5.786526	6.071266	8.712172
1	7.896946	10.01028	3.606073
1	7.887719	12.30993	2.682156
1	6.525036	14.09722	3.77213
1	5.166246	13.55699	5.798149
1	5.17667	11.24107	6.72407
1	8.171379	6.39464	5.161554
1	8.082204	4.917813	3.187011
1	6.432152	5.352212	1.359898
1	4.856147	7.28561	1.557642
1	4.922556	8.75111	3.537726
15	5.9021	8.806403	9.709626
15	6.559065	8.781833	5.99753
78	4.703644	8.513892	7.585783
6	2.942418	8.298502	8.663449
6	2.113779	9.404121	8.931379
6	2.501564	7.039967	9.115629
6	0.908143	9.264893	9.629917
1	2.408091	10.40186	8.588118
6	1.299296	6.893896	9.820981
1	3.100259	6.145426	8.913488
6	0.495204	8.007896	10.08369
1	0.286804	10.14743	9.819577
1	0.986136	5.89925	10.15854
1	-0.44851	7.895884	10.62804
6	3.438898	8.174354	5.974611
6	2.771005	9.228744	5.322692
6	3.177263	6.86875	5.518605

6	1.89845	8.9935	4.25139
1	2.924006	10.26132	5.654076
6	2.301764	6.625447	4.453167
1	3.663269	6.014723	6.002703
6	1.65976	7.68816	3.80898
1	1.395181	9.837859	3.766513
1	2.120238	5.595637	4.125387
1	0.972661	7.500442	2.977054
6	11.96765	9.323605	6.940409
6	13.04855	8.48689	7.289851
6	12.27475	10.6259	6.513687
6	14.36453	8.92964	7.218934
1	12.85805	7.458157	7.607397
6	13.59296	11.08448	6.433171
1	11.47018	11.31726	6.250841
6	14.65322	10.2361	6.787767
1	15.19772	8.273774	7.484745
1	13.77518	12.10761	6.100125
8	15.96595	10.58	6.74645
6	16.3295	11.86544	6.301923
1	15.91293	12.65941	6.95316
1	17.42911	11.90948	6.343258
1	16.00047	12.04874	5.260045
6	9.737244	10.26856	10.07218
1	10.77538	10.23704	10.40248
6	9.818502	12.47917	8.695558
6	10.97036	12.99762	9.30626
6	9.302719	13.18228	7.584791
6	11.59534	14.16092	8.842511
1	11.39296	12.49538	10.18045
6	9.912271	14.33788	7.112117
1	8.411623	12.81278	7.069943
6	11.0681	14.84308	7.736393
1	12.48562	14.52488	9.358518
1	9.508887	14.87587	6.250316
8	11.58981	15.97838	7.205817
6	12.73392	16.55281	7.792117
1	13.60382	15.86719	7.755008
1	12.96461	17.45554	7.20517
1	12.55389	16.84434	8.845602

Table S11. Transition state for the reductive elimination of **4**

Atomic number	Coordinates (Å)		
	X	Y	Z
6	7.464611	9.637889	9.708729
6	8.680026	9.268971	10.3809
6	9.104498	11.23512	9.207759
6	7.742764	10.84538	8.978792
6	8.223107	8.543741	6.57521
6	9.380895	9.351369	6.305494
6	10.51358	8.798087	6.992125
6	10.0409	7.646334	7.707228
6	8.649148	7.484988	7.451771
6	4.936405	9.754266	11.03194
6	3.719605	9.237826	11.51334
6	2.996298	9.92037	12.49428
6	3.466011	11.13679	13.00115
6	4.667656	11.66474	12.5203
6	5.399454	10.97884	11.54444
6	6.175796	7.203155	10.56465
6	6.515213	7.140008	11.92806
6	6.777295	5.9102	12.53887
6	6.697402	4.725061	11.79822
6	6.347561	4.775201	10.44576
6	6.084144	6.006562	9.834922
6	6.517517	10.41122	5.186045
6	7.232616	10.67168	4.003322
6	7.226285	11.94894	3.434937
6	6.499566	12.98349	4.03613
6	5.774968	12.73281	5.205505
6	5.781051	11.4533	5.773248
6	6.490827	7.618565	4.50189
6	7.495301	6.665454	4.258338
6	7.421599	5.822006	3.144229
6	6.346936	5.920094	2.255699
6	5.341365	6.863509	2.492279
6	5.407172	7.700731	3.608569
26	8.95668	9.31419	8.332724
1	8.823813	8.383267	10.99774
1	7.023531	11.38315	8.363776
1	9.39394	10.24262	5.680858
1	10.63602	7.014009	8.365631

1	8.010396	6.709099	7.87139
1	3.330504	8.293379	11.1237
1	2.053109	9.498691	12.85505
1	2.895939	11.67229	13.7671
1	5.046473	12.61526	12.90985
1	6.342057	11.40302	11.19111
1	6.566573	8.057854	12.52085
1	7.039854	5.876863	13.60115
1	6.898536	3.762478	12.27917
1	6.269462	3.851846	9.863145
1	5.788484	6.048222	8.781467
1	7.792275	9.868286	3.515869
1	7.787011	12.13532	2.513394
1	6.488585	13.981	3.585161
1	5.191793	13.53216	5.673617
1	5.196205	11.24738	6.675701
1	8.349484	6.58091	4.934069
1	8.215629	5.088833	2.968931
1	6.291875	5.262267	1.382407
1	4.489834	6.946393	1.809918
1	4.606476	8.425048	3.780054
15	5.827071	8.810758	9.708047
15	6.498152	8.734678	5.981504
78	4.706367	8.503233	7.58804
6	2.629931	8.136101	8.137855
6	1.830879	9.204597	8.629418
6	2.380078	6.858152	8.714712
6	0.841902	9.006633	9.591321
1	1.972469	10.21132	8.22709
6	1.401039	6.664589	9.687632
1	2.96205	5.994184	8.381658
6	0.60937	7.734218	10.12927
1	0.245906	9.861679	9.928175
1	1.248366	5.661818	10.10155
1	-0.17531	7.577277	10.87572
6	2.941222	8.166797	6.355331
6	2.476776	9.313962	5.650526
6	2.770116	6.920919	5.691595
6	1.877288	9.218745	4.395877
1	2.579035	10.30314	6.10558
6	2.156537	6.825745	4.443807

1	3.103659	6.001197	6.179675
6	1.697157	7.971452	3.781312
1	1.538089	10.13153	3.893887
1	2.038773	5.840172	3.980573
1	1.204833	7.894788	2.806997
6	11.90646	9.288021	6.940835
6	12.98171	8.441964	7.254777
6	12.21628	10.60784	6.54765
6	14.31047	8.876279	7.189942
1	12.789	7.404702	7.542322
6	13.5303	11.05294	6.475725
1	11.41129	11.3084	6.312058
6	14.59545	10.19223	6.797182
1	15.10965	8.17579	7.439192
1	13.76244	12.079	6.179347
8	15.84367	10.71718	6.698051
6	16.95726	9.913492	7.007679
1	17.02723	9.031984	6.340014
1	17.84746	10.54491	6.8605
1	16.93348	9.564596	8.059118
6	9.679224	10.23445	10.06222
1	10.71803	10.19607	10.38978
6	9.770709	12.44472	8.683629
6	10.94759	12.94411	9.278705
6	9.248516	13.15961	7.592637
6	11.57169	14.09307	8.805418
1	11.37986	12.42922	10.14063
6	9.864231	14.31647	7.104995
1	8.343688	12.80582	7.090842
6	11.03743	14.79448	7.710767
1	12.48211	14.47593	9.273767
1	9.418918	14.83088	6.251544
8	11.71293	15.9047	7.318856
6	11.22844	16.66231	6.235267
1	10.21463	17.06259	6.433883
1	11.92687	17.50407	6.107476
1	11.20264	16.07114	5.298508

Table S12. Minimal energy conformation for **2b**

Atomic number	Coordinates (Å)		

	X	Y	Z
6	-0.15142	10.47697	2.393938
6	0.499305	8.026941	3.544723
6	1.166453	10.28819	2.858789
6	1.465765	9.035303	3.431565
6	3.278538	13.96792	5.353911
6	2.614389	11.36832	-0.2858
6	3.224147	11.01447	0.935027
6	3.743484	14.76415	4.094498
6	3.071442	10.86461	-1.51111
6	4.424406	13.22701	6.084402
6	4.324521	13.27923	7.606817
6	4.311994	10.11979	0.867635
6	4.156485	9.982855	-1.55257
6	4.371087	16.94812	5.319532
6	4.805836	15.84744	4.353473
6	5.440053	12.54057	9.544221
6	4.773127	9.610139	-0.35395
6	5.090036	19.01997	6.177586
6	-0.80361	8.240928	3.082622
6	-1.12326	9.472815	2.502848
1	0.769941	7.063261	3.991709
1	1.752054	12.04507	-0.28683
1	2.817759	14.69121	6.052644
1	2.480756	8.830736	3.791499
1	2.847034	15.28276	3.701503
1	2.569452	11.15867	-2.44021
1	3.358505	12.86021	7.961887
1	4.483874	12.16843	5.771712
1	3.385291	17.36461	5.008023
1	4.528067	12.07712	9.980278
1	4.8111	9.799347	1.789845
1	6.317204	11.94481	9.845915
1	5.399875	13.6662	5.818635
1	4.14265	19.54072	5.916575
1	4.239939	16.54748	6.349176
1	4.513051	9.584727	-2.50847
1	5.058272	16.34548	3.400662
1	4.356408	14.3395	7.949242
1	5.747698	15.41087	4.726803
1	5.618326	8.912161	-0.36685

1	5.541872	13.56394	9.967706
1	5.019768	18.68926	7.237184
1	5.923123	19.73626	6.086174
1	-0.43159	11.42684	1.923008
1	-1.55969	7.453087	3.166678
1	-2.13655	9.654161	2.125816
8	5.400128	12.55621	8.143121
8	5.35447	17.94986	5.311819
15	1.909315	12.77724	4.802963
15	4.146284	13.58841	2.661343
78	2.583621	11.80926	2.749495
6	0.399402	13.84086	4.737417
1	-0.45314	13.20531	4.44139
1	0.18065	14.31145	5.713168
1	0.515127	14.62456	3.970352
6	1.530753	11.68387	6.236868
1	2.404998	11.06247	6.488697
1	1.203382	12.24886	7.127125
1	0.723683	11.00226	5.917059
6	4.084306	14.67836	1.175578
1	4.823254	15.49799	1.214823
1	4.280369	14.05172	0.28856
1	3.070506	15.10038	1.070568
6	5.939244	13.16956	2.774589
1	6.177375	12.51958	1.914549
1	6.58666	14.06325	2.746332
1	6.138867	12.59829	3.695431

Table S13. Transition state for the reductive elimination of **2b**

Atomic number	Coordinates (Å)		
	X	Y	Z
6	0.081297	10.20375	1.791832
6	0.647535	7.991049	3.386992
6	1.433044	9.999692	2.194155
6	1.674485	8.848817	2.998773
6	3.243835	13.90793	5.306233
6	2.271178	10.97136	-0.23874
6	2.706822	10.45136	1.014668
6	3.716714	14.72004	4.058786
6	2.95334	10.70791	-1.42465

6	4.38124	13.12258	6.004803
6	4.340818	13.19779	7.528586
6	3.868841	9.627196	0.974414
6	4.101245	9.903014	-1.42917
6	4.338408	16.89822	5.29952
6	4.784872	15.78997	4.347496
6	5.488123	12.43367	9.436933
6	4.548364	9.366431	-0.21366
6	5.063451	18.95661	6.184088
6	-0.67498	8.226633	2.985662
6	-0.94302	9.343969	2.182498
1	0.882698	7.122823	4.012823
1	1.376552	11.59934	-0.27871
1	2.811887	14.62882	6.026033
1	2.69364	8.627338	3.328209
1	2.825719	15.25416	3.674342
1	2.582524	11.13689	-2.36236
1	3.369649	12.82976	7.923949
1	4.377879	12.05872	5.703967
1	3.371931	17.33362	4.955306
1	4.570676	12.02242	9.912765
1	4.246394	9.185016	1.900805
1	6.345527	11.8004	9.719106
1	5.366828	13.50906	5.697279
1	4.134607	19.49421	5.891517
1	4.165259	16.50144	6.324191
1	4.629958	9.688746	-2.36294
1	5.074038	16.28512	3.403555
1	4.436185	14.25881	7.856188
1	5.708944	15.34109	4.749805
1	5.441849	8.732431	-0.19139
1	5.654028	13.45707	9.840196
1	4.949959	18.62665	7.24039
1	5.911154	19.65919	6.123633
1	-0.16771	11.0609	1.159705
1	-1.47878	7.546125	3.282733
1	-1.96829	9.547909	1.853752
8	5.399682	12.43002	8.038896
8	5.339627	17.88365	5.326915
15	1.835817	12.75561	4.760145
15	4.112047	13.56608	2.597384

78	2.545589	11.77844	2.711572
6	0.379666	13.903	4.6978
1	-0.52261	13.30808	4.472936
1	0.227113	14.441	5.652167
1	0.505259	14.63562	3.883189
6	1.412068	11.75835	6.261544
1	2.244342	11.08429	6.521546
1	1.151642	12.38087	7.137094
1	0.545849	11.12602	5.999109
6	4.039677	14.72235	1.153604
1	4.737525	15.57513	1.240465
1	4.286561	14.1485	0.243514
1	3.010213	15.10283	1.040205
6	5.927299	13.21199	2.693377
1	6.197378	12.62314	1.79925
1	6.546509	14.12635	2.722675
1	6.144967	12.59362	3.579376

Table S14. Minimal energy conformation for **1b**

Atomic number	Coordinates (Å)		
	X	Y	Z
78	0.304019	-2.36412	0.430535
6	0.189581	1.753609	0.718531
6	1.235752	0.968142	1.247663
6	1.72658	1.24865	2.539137
1	2.526367	0.645533	2.970451
6	1.196003	2.294237	3.290032
1	1.589888	2.501179	4.290044
6	0.161081	3.081988	2.78097
6	-0.34173	2.810379	1.502584
6	-0.38741	1.565567	-0.65349
6	-1.24634	0.503738	-1.00876
6	-1.75558	0.435467	-2.3216
1	-2.411	-0.38376	-2.61933
6	-1.42679	1.406769	-3.2635
1	-1.83273	1.341212	-4.27786
6	-0.57945	2.465121	-2.92796
6	-0.05951	2.541649	-1.63002
15	1.864803	-0.54061	0.369698
15	-1.58537	-0.90702	0.148027

8	0.793275	3.516004	-1.22447
8	-1.36347	3.503771	0.940081
6	1.138133	4.559078	-2.10595
1	1.811306	5.226351	-1.54554
1	1.67008	4.183019	-3.00169
6	-1.92554	4.601587	1.620364
1	-2.38728	4.298992	2.580561
1	-1.17396	5.391251	1.815456
1	-0.32754	3.215034	-3.67913
1	-0.24713	3.894078	3.384258
1	0.24787	5.132442	-2.43027
1	-2.70852	5.005501	0.959847
6	1.864607	-3.72135	0.627638
6	2.255291	-4.2276	1.883255
1	1.700775	-3.93592	2.782328
6	2.597491	-4.16055	-0.49387
1	2.321424	-3.81086	-1.49591
6	3.676532	-5.04672	-0.36806
1	4.221986	-5.36928	-1.26264
6	4.054548	-5.5242	0.891024
1	4.895828	-6.21796	0.992818
6	3.334104	-5.11145	2.016975
1	3.608734	-5.48659	3.0097
6	-0.95975	-4.00887	0.55157
6	-1.61385	-4.34164	1.75539
1	-1.42887	-3.74696	2.658002
6	-1.22192	-4.82807	-0.56455
1	-0.71843	-4.62534	-1.51657
6	-2.10628	-5.91213	-0.49012
1	-2.28486	-6.52908	-1.37845
6	-2.75351	-6.21612	0.712356
1	-3.44238	-7.06529	0.773757
6	-2.49826	-5.42631	1.838051
1	-2.98709	-5.65749	2.791695
6	-3.08647	-1.71355	-0.55756
1	-2.83756	-2.26122	-1.47966
1	-3.42699	-2.46259	0.176078
1	-3.89288	-0.98523	-0.75134
6	-2.29787	-0.15684	1.682875
1	-3.07901	0.58596	1.444792
1	-2.73901	-0.98026	2.27136

1	-1.51084	0.317193	2.287308
6	2.429122	0.028537	-1.29909
1	3.034884	0.948701	-1.22869
1	3.041492	-0.78355	-1.72845
1	1.570877	0.202858	-1.96416
6	3.490708	-0.88022	1.171693
1	3.349241	-1.27861	2.188219
1	3.982366	-1.67324	0.584574
1	4.13146	0.018116	1.19878

Table S15. Transition state for the reductive elimination of **1b**

Atomic number	Coordinates (Å)		
	X	Y	Z
78	0.365508	-2.29707	0.078492
6	0.198598	1.736533	0.52146
6	1.339554	0.997279	0.909876
6	1.96933	1.292777	2.137031
1	2.844804	0.726711	2.4579
6	1.485694	2.302673	2.96342
1	1.98883	2.519983	3.911074
6	0.359298	3.042704	2.595835
6	-0.27849	2.760014	1.381696
6	-0.53174	1.562241	-0.78111
6	-1.43108	0.508805	-1.06486
6	-2.09833	0.485602	-2.30742
1	-2.78796	-0.32467	-2.54786
6	-1.88654	1.486597	-3.25072
1	-2.41483	1.453848	-4.20898
6	-1.00193	2.535236	-2.98719
6	-0.33052	2.57146	-1.75887
15	1.907043	-0.48197	-0.05088
15	-1.60178	-0.94851	0.066004
8	0.558414	3.543257	-1.42632
8	-1.389	3.416053	0.955586
6	0.790972	4.613795	-2.30984
1	1.521174	5.271353	-1.81257
1	1.216605	4.269754	-3.27304
6	-1.90905	4.481512	1.713986
1	-2.2421	4.150795	2.717458
1	-1.17176	5.299661	1.832386

1	-0.84148	3.309588	-3.73864
1	-0.01244	3.826737	3.25701
1	-0.13427	5.18849	-2.51149
1	-2.78012	4.860608	1.15686
6	1.523193	-4.11772	0.135824
6	2.233326	-4.37416	1.344523
1	1.765442	-4.13295	2.303367
6	2.196584	-4.47896	-1.06739
1	1.699547	-4.3211	-2.02884
6	3.477672	-5.02701	-1.06112
1	3.956634	-5.28043	-2.01362
6	4.15368	-5.2587	0.145513
1	5.154813	-5.70043	0.149377
6	3.513144	-4.92569	1.347159
1	4.019789	-5.09939	2.303195
6	-0.25935	-4.36122	0.176227
6	-0.82315	-4.79566	1.411018
1	-0.3966	-4.436	2.351758
6	-0.86119	-4.89267	-1.00114
1	-0.46467	-4.61081	-1.98083
6	-1.94561	-5.76637	-0.94704
1	-2.37659	-6.14404	-1.88112
6	-2.48573	-6.16457	0.283696
1	-3.33118	-6.85805	0.325006
6	-1.90958	-5.66671	1.461287
1	-2.31268	-5.96478	2.435723
6	-2.1899	-0.25308	1.681174
1	-3.0245	0.456313	1.540383
1	-2.52861	-1.10042	2.302815
1	-1.36557	0.251271	2.206764
6	-3.14408	-1.79657	-0.50209
1	-3.35714	-2.59255	0.23218
1	-4.01212	-1.11698	-0.57098
1	-2.97691	-2.29292	-1.47123
6	3.600703	-0.83573	0.602547
1	4.265082	0.046524	0.584316
1	3.54333	-1.24171	1.625073
1	4.026334	-1.63048	-0.03416
6	2.33679	0.16216	-1.73566
1	2.965781	1.06809	-1.67813
1	2.888073	-0.63512	-2.26447

1	1.423646	0.386064	-2.30695

Table S16. Minimal energy conformation for **3**

Atomic number	Coordinates (Å)		
	X	Y	Z
78	0.306848	-2.35216	0.431761
6	0.215873	1.826514	0.737466
6	1.279864	1.056265	1.221415
6	1.850429	1.366029	2.492782
1	2.691703	0.7805	2.862332
6	1.373896	2.398614	3.264067
1	1.836267	2.618514	4.231817
6	0.287754	3.193175	2.820937
6	-0.30092	2.91315	1.542604
6	-0.38671	1.644112	-0.62959
6	-1.27687	0.612314	-0.94945
6	-1.87346	0.575325	-2.24582
1	-2.58189	-0.21569	-2.48993
6	-1.58782	1.524379	-3.19762
1	-2.06637	1.475652	-4.18113
6	-0.68006	2.576882	-2.92251
6	-0.07133	2.647064	-1.62496
6	3.503081	-0.85122	1.167322
6	3.473364	-1.52583	2.402067
1	2.514896	-1.82272	2.834531
6	4.65561	-1.83935	3.075169
1	4.60838	-2.36896	4.031449
6	5.892825	-1.50286	2.515923
1	6.820717	-1.75947	3.036765
6	5.93583	-0.85402	1.279864
1	6.898375	-0.6001	0.824577
6	4.750578	-0.53051	0.609451
1	4.812684	-0.03679	-0.3617
6	2.348978	0.054378	-1.35235
6	2.021719	-0.77916	-2.43496
1	1.447598	-1.69246	-2.25152
6	2.412989	-0.44248	-3.7348
1	2.150023	-1.10166	-4.5678
6	3.134528	0.732123	-3.96777
1	3.441602	0.995995	-4.98478

6	3.455055	1.57473	-2.89776
1	4.006717	2.503352	-3.07376
6	-3.09838	-1.64145	-0.51665
6	-2.91628	-2.50879	-1.60987
1	-1.91046	-2.68945	-1.99666
6	-4.00345	-3.159	-2.19665
1	-3.83735	-3.83305	-3.04238
6	-5.29275	-2.97081	-1.68776
1	-6.14415	-3.49004	-2.13901
6	-5.48299	-2.13058	-0.58845
1	-6.48493	-1.98824	-0.17121
6	-4.39471	-1.47066	-0.00611
1	-4.56883	-0.83152	0.861057
6	-2.18554	-0.09105	1.779284
6	-3.11852	0.959819	1.817715
1	-3.47749	1.408919	0.887598
6	-3.59031	1.445201	3.040133
1	-4.31419	2.265852	3.053161
6	-3.13019	0.892584	4.240332
1	-3.49918	1.27506	5.197305
6	-2.19078	-0.14249	4.213263
1	-1.8185	-0.57292	5.147976
6	-1.72032	-0.63088	2.990081
1	-0.9764	-1.43299	2.966319
6	3.061798	1.240611	-1.59921
1	3.308869	1.913909	-0.77382
15	1.87958	-0.48564	0.348032
15	-1.59037	-0.83189	0.198029
6	1.826184	-3.75205	0.65927
6	1.9235	-4.5362	1.826725
1	1.177615	-4.42212	2.620017
6	2.790433	-3.98036	-0.34147
1	2.764742	-3.40549	-1.27323
6	3.807364	-4.93209	-0.18268
1	4.539106	-5.0808	-0.98511
6	3.893467	-5.68478	0.991471
1	4.68796	-6.42773	1.119113
6	2.943111	-5.47989	1.997497
1	2.98581	-6.07035	2.920068
6	-0.91996	-4.02863	0.49398
6	-1.83978	-4.24528	1.538177

1	-1.93671	-3.51448	2.348031
6	-0.85032	-5.01826	-0.50757
1	-0.12684	-4.91465	-1.32277
6	-1.67199	-6.15117	-0.48221
1	-1.58871	-6.89873	-1.2796
6	-2.58273	-6.34163	0.562535
1	-3.22168	-7.23074	0.588967
6	-2.65773	-5.38311	1.576545
1	-3.36111	-5.51588	2.406651
6	-0.36492	3.565826	-3.89027
6	0.83015	3.712408	-1.37175
6	0.514826	4.593057	-3.61777
1	0.732877	5.335912	-4.38692
6	1.126094	4.6642	-2.33694
1	-0.83731	3.503717	-4.87583
1	1.323216	3.80955	-0.40489
8	2.015964	5.628611	-1.98178
6	2.340272	6.653994	-2.89083
1	3.055827	7.311367	-2.37251
1	2.819056	6.259071	-3.80872
1	1.450984	7.24913	-3.17765
6	-1.38379	3.725194	1.118628
6	-0.22595	4.261152	3.601556
6	-1.87167	4.7604	1.903095
6	-1.28001	5.03696	3.165105
1	-1.64833	5.850249	3.792694
1	-1.86967	3.552371	0.158721
1	0.232689	4.467228	4.573929
8	-2.92248	5.458785	1.39694
6	-3.45195	6.548807	2.114212
1	-4.27078	6.954506	1.499597
1	-3.86227	6.239846	3.095989
1	-2.69761	7.344086	2.274809

Table S17. Transition state for the reductive elimination of **3**

Atomic number	Coordinates (Å)		
	X	Y	Z
78	0.217282	-2.309	0.176216
6	0.022993	1.841126	0.610368
6	1.072693	1.139924	1.217722

6	1.582553	1.586948	2.47379
1	2.408073	1.048524	2.940194
6	1.063129	2.689953	3.109015
1	1.477072	3.017063	4.068449
6	-0.01116	3.41747	2.537258
6	-0.54032	2.995389	1.271786
6	-0.51331	1.481706	-0.74847
6	-1.37016	0.397555	-0.96426
6	-1.80829	0.095028	-2.2925
1	-2.43216	-0.7844	-2.46276
6	-1.45522	0.881165	-3.36055
1	-1.80866	0.63368	-4.36682
6	-0.62342	2.01739	-3.18085
6	-0.1351	2.322023	-1.86549
6	3.074813	-0.94036	1.666239
6	2.67418	-1.61972	2.832658
1	1.610458	-1.80878	3.006013
6	3.614211	-2.05668	3.768297
1	3.279801	-2.57459	4.672963
6	4.979665	-1.84693	3.541963
1	5.719203	-2.19816	4.268545
6	5.391183	-1.19497	2.376856
1	6.456829	-1.03438	2.18443
6	4.447246	-0.74159	1.447391
1	4.791232	-0.23311	0.54409
6	2.740384	0.192114	-0.97587
6	2.913318	-0.65025	-2.08589
1	2.448153	-1.6389	-2.09048
6	3.684372	-0.23619	-3.17689
1	3.811752	-0.90541	-4.03356
6	4.286394	1.025784	-3.17448
1	4.885673	1.351024	-4.03106
6	4.11689	1.873251	-2.07415
1	4.575011	2.86729	-2.06692
6	-3.40588	-1.54841	-0.126
6	-3.69157	-2.88161	0.209087
1	-2.90919	-3.51733	0.629591
6	-4.97299	-3.40761	0.008106
1	-5.17183	-4.45013	0.274283
6	-5.98335	-2.61153	-0.53656
1	-6.98416	-3.02453	-0.69892

6	-5.70887	-1.28135	-0.87632
1	-6.49436	-0.64922	-1.30316
6	-4.43256	-0.75235	-0.66897
1	-4.23852	0.288755	-0.94129
6	-2.12663	-0.12114	1.930506
6	-3.07298	0.908544	2.068258
1	-3.53571	1.353558	1.183968
6	-3.43516	1.37498	3.333638
1	-4.16912	2.181864	3.423314
6	-2.86514	0.812481	4.481846
1	-3.15469	1.175902	5.473076
6	-1.92853	-0.21665	4.356653
1	-1.48081	-0.66536	5.249043
6	-1.5618	-0.67923	3.087917
1	-0.83673	-1.49261	2.986515
6	3.350172	1.458945	-0.98146
1	3.226378	2.129833	-0.12702
15	1.758847	-0.42798	0.462171
15	-1.71692	-0.90759	0.302055
6	1.457627	-4.02077	-0.29365
6	2.437518	-4.45536	0.637744
1	2.221032	-4.4123	1.708179
6	1.805733	-4.13464	-1.66826
1	1.081989	-3.83654	-2.43223
6	3.046878	-4.62249	-2.07646
1	3.273043	-4.68846	-3.14655
6	3.998246	-5.02983	-1.13294
1	4.969085	-5.42061	-1.45278
6	3.678042	-4.94043	0.228562
1	4.406892	-5.2521	0.984417
6	-0.28407	-4.40314	0.109478
6	-0.51865	-4.9656	1.396507
1	0.054855	-4.60158	2.253982
6	-1.05662	-4.94387	-0.95567
1	-0.91744	-4.56094	-1.97018
6	-1.98682	-5.96039	-0.75034
1	-2.5574	-6.3434	-1.60353
6	-2.20237	-6.48869	0.530517
1	-2.92744	-7.29291	0.688746
6	-1.46028	-5.97363	1.601916
1	-1.61186	-6.36764	2.612938

6	-1.6202	3.731095	0.720392
6	0.714175	3.447108	-1.71695
6	-2.16083	4.830706	1.37167
6	1.071778	4.240581	-2.79795
6	-1.62869	5.248915	2.621624
1	-2.03921	6.113763	3.145596
6	0.576454	3.94184	-4.09452
1	0.84398	4.557372	-4.95501
6	-0.57857	4.548217	3.179491
1	-0.16801	4.864802	4.143706
6	-0.2505	2.849498	-4.26649
1	-0.63033	2.610025	-5.26495
1	1.116431	3.717117	-0.74112
1	-2.05694	3.445369	-0.23679
8	-3.19865	5.452098	0.751223
8	1.908642	5.280118	-2.53309
6	-3.78923	6.587382	1.337926
1	-4.58888	6.90893	0.652308
1	-4.23658	6.358895	2.325619
1	-3.06461	7.417164	1.455665
6	2.29117	6.151901	-3.56994
1	2.946559	6.909181	-3.11153
1	2.854852	5.627287	-4.36668
1	1.41924	6.662382	-4.02462

Table S18. Minimal energy conformation for **5**

Atomic number	Coordinates (Å)		
	X	Y	Z
6	0.153587	11.2206	2.930674
6	0.683214	8.831014	4.25576
6	1.386471	11.09081	3.601401
6	1.628628	9.865181	4.25347
6	0.006199	14.07763	5.19379
6	2.991573	11.86728	0.600623
6	3.510807	11.61719	1.886628
6	6.245519	13.46081	3.512209
6	3.456454	11.18115	-0.52908
6	-0.85237	14.57458	6.361516
6	-2.24344	15.00877	5.909853
6	4.50714	10.62538	1.987553

6	4.457798	10.2121	-0.40523
6	8.781692	13.55117	3.535652
6	7.491125	14.31715	3.261236
6	-4.25643	15.9024	6.73618
6	4.978115	9.93418	0.862402
6	11.11929	13.8139	3.534531
6	-0.53992	8.990017	3.595781
6	-0.79799	10.19108	2.928104
1	0.908682	7.890609	4.771634
1	2.197729	12.61173	0.470946
1	-0.48856	13.2384	4.672665
1	2.582165	9.703698	4.769152
1	6.218371	13.11489	4.562956
1	3.026478	11.40131	-1.51294
1	-2.77723	14.1543	5.436155
1	-0.9747	13.78683	7.125761
1	8.788277	13.17364	4.583821
1	-4.87809	15.08086	6.317025
1	4.921578	10.36885	2.969506
1	-4.71489	16.24537	7.678727
1	-0.37972	15.43575	6.866568
1	11.23324	13.45963	4.582958
1	8.846569	12.6586	2.873396
1	4.820864	9.672141	-1.28599
1	7.492322	15.21672	3.902199
1	-2.16416	15.80603	5.135701
1	7.52328	14.67252	2.216087
1	5.752076	9.167176	0.981014
1	-4.2668	16.74433	6.009035
1	11.28972	12.94618	2.85992
1	11.89284	14.57376	3.333438
1	-0.07184	12.14154	2.380363
1	-1.27935	8.182334	3.593199
1	-1.74444	10.32791	2.392483
8	-2.95402	15.47779	7.027177
8	9.871137	14.41029	3.316068
15	1.72576	13.46876	5.560214
15	4.57586	14.21759	3.192974
78	2.803571	12.62026	3.572236
6	1.490358	12.18136	6.864217
1	2.48147	11.80583	7.172654

1	0.968491	12.58353	7.749586
1	0.923626	11.33382	6.44706
6	2.445451	14.84392	6.575796
1	2.472391	15.77894	5.99348
1	1.859148	15.01465	7.495197
1	3.477935	14.58267	6.86355
6	4.672423	15.83438	4.097175
1	5.487085	16.46661	3.703802
1	3.71946	16.37713	3.977106
1	4.84327	15.66476	5.172336
6	4.66685	14.80937	1.445256
1	3.721977	15.32285	1.196306
1	5.505997	15.50966	1.292275
1	4.769236	13.94631	0.768465
1	6.267054	12.54592	2.893081
1	0.128752	14.87463	4.435943

Table S19. Transition state for the reductive elimination of **5**

Atomic number	Coordinates (Å)		
	X	Y	Z
6	0.40519	11.32575	2.527798
6	0.537159	9.180481	4.298223
6	1.65318	11.06332	3.162252
6	1.671694	9.952265	4.053089
6	-0.18029	13.92915	5.15359
6	2.924585	11.67737	0.785598
6	3.142572	11.30478	2.142726
6	6.312182	13.3569	3.340629
6	3.720764	11.19502	-0.25192
6	-1.14597	14.26966	6.293589
6	-2.5638	14.52556	5.792275
6	4.216706	10.39724	2.369434
6	4.775652	10.30784	0.00538
6	8.836902	13.23657	3.102843
6	7.589392	14.09734	2.93008
6	-4.71925	15.08684	6.548894
6	5.012284	9.916086	1.329968
6	11.17319	13.29198	2.833997
6	-0.6788	9.469176	3.662424
6	-0.72867	10.55208	2.774281

1	0.603227	8.337083	4.994717
1	2.105434	12.36006	0.541451
1	-0.52452	13.03322	4.604127
1	2.603524	9.68744	4.561293
1	6.379588	13.02439	4.394167
1	3.513064	11.51423	-1.27941
1	-2.94957	13.62704	5.25978
1	-1.19152	13.44761	7.029427
1	8.931617	12.90579	4.162469
1	-5.19703	14.20547	6.066715
1	4.428106	10.06023	3.388572
1	-5.26504	15.31819	7.478975
1	-0.81454	15.16879	6.843575
1	11.37498	12.97461	3.881174
1	8.754229	12.3136	2.485554
1	5.392792	9.921215	-0.81122
1	7.728067	15.01609	3.527857
1	-2.56597	15.36058	5.054769
1	7.539812	14.41232	1.872837
1	5.828127	9.220714	1.557502
1	-4.81824	15.95094	5.854944
1	11.19425	12.38368	2.192034
1	11.98014	13.97061	2.510345
1	0.327134	12.15717	1.820993
1	-1.56566	8.85546	3.846884
1	-1.6658	10.79655	2.261499
8	-3.38439	14.83846	6.889669
8	9.962784	13.98612	2.720745
15	1.605204	13.58799	5.565305
15	4.679574	14.24664	3.209169
78	2.836916	12.80893	3.679441
6	1.513525	12.34771	6.936253
1	2.537244	12.1385	7.292282
1	0.902471	12.69449	7.788103
1	1.09405	11.40748	6.538763
6	2.114977	15.11455	6.488758
1	2.080843	15.98411	5.810236
1	1.47297	15.31632	7.364695
1	3.156036	14.99312	6.833312
6	4.971705	15.79513	4.189144
1	5.832319	16.37852	3.816222

1	4.067821	16.42587	4.138739
1	5.145831	15.53481	5.247251
6	4.674612	14.90768	1.480389
1	3.769288	15.52356	1.340236
1	5.563465	15.5226	1.254322
1	4.620805	14.0609	0.774696
1	6.184979	12.43467	2.743505
1	-0.15741	14.74713	4.40823

Table S20. Calculated reductive elimination barrier lowering of (P-P)PtPh$_2$ complexes vs force.

Restoring force (pN)	ΔE(f) (kcal/mol)						
	**2a**	**4**	**2b**	**1b**	**3**	**5**	**1a**
-50	-0.164	-0.0239	-0.00159	0.055	0.201	0.178	0.168
-40	-0.13	-0.0191	-0.00103	0.0438	0.159	0.142	0.134
-30	-0.0962	-0.0143	-0.00059	0.0328	0.118	0.106	0.1
-20	-0.0635	-0.00949	-0.00028	0.0218	0.0777	0.07	0.0668
-10	-0.0314	-0.00473	-7.88E-05	0.0108	0.0384	0.0348	0.0333
0	0	0	0	0	0	0	0
10	0.0308	0.00471	-3.70E-05	-0.0108	-0.0376	-0.0582	-0.0331
20	0.0609	0.00939	-0.00019	-0.0214	-0.0743	-0.113	-0.066
30	0.0904	0.014	-0.00045	-0.032	-0.11	-0.165	-0.0988
40	0.119	0.0187	-0.00082	-0.0426	-0.145	-0.213	-0.131
50	0.148	0.0233	-0.00131	-0.053	-0.18	-0.259	-0.164
60	0.175	0.0279	-0.00189	-0.0634	-0.213	-0.301	-0.196
70	0.202	0.0324	-0.00258	-0.0737	-0.246	-0.34	-0.228
80	0.229	0.0369	-0.00338	-0.0839	-0.278	-0.377	-0.26
90	0.254	0.0414	-0.00427	-0.094	-0.309	-0.411	-0.292
100	0.28	0.0459	-0.00527	-0.104	-0.34	-0.442	-0.323
110	0.304	0.0504	-0.00636	-0.114	-0.37	-0.47	-0.354
120	0.328	0.0548	-0.00754	-0.124	-0.399	-0.497	-0.385
130	0.352	0.0592	-0.00882	-0.134	-0.427	-0.525	-0.416
140	0.375	0.0636	-0.0102	-0.143	-0.455	-0.551	-0.447
150	0.397	0.0679	-0.0117	-0.153	-0.481	-0.578	-0.478
160	0.419	0.0722	-0.0132	-0.163	-0.508	-0.604	-0.508
170	0.44	0.0765	-0.0148	-0.172	-0.533	-0.631	-0.538
180	0.461	0.0808	-0.0165	-0.182	-0.558	-0.656	-0.568
190	0.481	0.085	-0.0183	-0.191	-0.582	-0.682	-0.598
200	0.501	0.0892	-0.0202	-0.2	-0.606	-0.707	-0.628
210	0.52	0.0934	-0.0222	-0.209	-0.628	-0.732	-0.658

220	0.538	0.0976	-0.0242	-0.218	-0.651	-0.757	-0.687
230	0.556	0.102	-0.0263	-0.227	-0.672	-0.781	-0.716
240	0.574	0.106	-0.0285	-0.236	-0.693	-0.806	-0.745
250	0.591	0.11	-0.0307	-0.245	-0.713	-0.83	-0.774
260	0.607	0.114	-0.0331	-0.254	-0.733	-0.853	-0.802
270	0.623	0.118	-0.0354	-0.263	-0.752	-0.876	-0.831
280	0.639	0.122	-0.0379	-0.271	-0.771	-0.899	-0.859
290	0.654	0.126	-0.0404	-0.28	-0.788	-0.922	-0.887
300	0.668	0.13	-0.043	-0.288	-0.806	-0.945	-0.915
310	0.682	0.134	-0.0456	-0.297	-0.823	-0.967	-0.943
320	0.696	0.138	-0.0483	-0.305	-0.839	-0.989	-0.97
330	0.709	0.142	-0.051	-0.313	-0.855	-1.01	-0.998
340	0.722	0.146	-0.0538	-0.321	-0.87	-1.03	-1.02
350	0.734	0.149	-0.0567	-0.329	-0.884	-1.05	-1.05
360	0.746	0.153	-0.0596	-0.337	-0.898	-1.07	-1.08
370	0.757	0.157	-0.0625	-0.345	-0.912	-1.09	-1.11
380	0.768	0.161	-0.0655	-0.353	-0.925	-1.11	-1.13
390	0.778	0.164	-0.0685	-0.361	-0.938	-1.13	-1.16
400	0.788	0.168	-0.0716	-0.368	-0.95	-1.15	-1.18
410	0.798	0.172	-0.0747	-0.376	-0.961	-1.17	-1.21
420	0.807	0.175	-0.0779	-0.384	-0.973	-1.19	-1.24
430	0.816	0.179	-0.081	-0.391	-0.983	-1.21	-1.26
440	0.824	0.183	-0.0843	-0.398	-0.994	-1.23	-1.29
450	0.832	0.186	-0.0875	-0.406	-1	-1.25	-1.31
460	0.84	0.19	-0.0908	-0.413	-1.01	-1.27	-1.34
470	0.847	0.193	-0.0941	-0.42	-1.02	-1.28	-1.36
480	0.854	0.197	-0.0975	-0.427	-1.03	-1.3	-1.39
490	0.86	0.2	-0.101	-0.434	-1.04	-1.32	-1.41
500	0.866	0.204	-0.104	-0.441	-1.05	-1.34	-1.44
510	0.872	0.207	-0.108	-0.448	-1.05	-1.35	-1.46
520	0.877	0.211	-0.111	-0.454	-1.06	-1.37	-1.48
530	0.882	0.214	-0.115	-0.461	-1.07	-1.39	-1.51
540	0.887	0.217	-0.118	-0.467	-1.07	-1.4	-1.53
550	0.891	0.221	-0.121	-0.474	-1.08	-1.42	-1.55
560	0.895	0.224	-0.125	-0.48	-1.09	-1.44	-1.58
570	0.899	0.227	-0.128	-0.487	-1.09	-1.45	-1.6
580	0.902	0.23	-0.132	-0.493	-1.1	-1.47	-1.62
590	0.905	0.234	-0.135	-0.499	-1.1	-1.48	-1.65
600	0.908	0.237	-0.139	-0.505	-1.1	-1.5	-1.67
610	0.91	0.24	-0.142	-0.511	-1.11	-1.51	-1.69
620	0.912	0.243	-0.146	-0.517	-1.11	-1.53	-1.71

630	0.914	0.246	-0.149	-0.523	-1.12	-1.54	-1.73
640	0.916	0.249	-0.153	-0.529	-1.12	-1.56	-1.76
650	0.917	0.252	-0.157	-0.534	-1.12	-1.57	-1.78
660	0.918	0.255	-0.16	-0.54	-1.12	-1.59	-1.8
670	0.918	0.258	-0.163	-0.545	-1.13	-1.6	-1.82
680	0.919	0.261	-0.167	-0.551	-1.13	-1.62	-1.84
690	0.919	0.264	-0.17	-0.556	-1.13	-1.63	-1.86
700	0.919	0.267	-0.174	-0.561	-1.13	-1.65	-1.88
710	0.918	0.27	-0.177	-0.567	-1.13	-1.66	-1.9
720	0.917	0.273	-0.181	-0.572	-1.14	-1.68	-1.92
730	0.917	0.276	-0.184	-0.577	-1.14	-1.69	-1.94
740	0.915	0.279	-0.187	-0.582	-1.14	-1.7	-1.96
750	0.914	0.281	-0.191	-0.587	-1.14	-1.72	-1.98
760	0.912	0.284	-0.194	-0.591	-1.14	-1.73	-2
770	0.911	0.287	-0.197	-0.596	-1.14	-1.75	-2.02
780	0.909	0.29	-0.201	-0.601	-1.14	-1.76	-2.04
790	0.906	0.292	-0.204	-0.605	-1.14	-1.78	-2.06
800	0.904	0.295	-0.207	-0.61	-1.14	-1.79	-2.08
810	0.901	0.298	-0.21	-0.614	-1.14	-1.81	-2.1
820	0.898	0.3	-0.213	-0.619	-1.14	-1.82	-2.12
830	0.895	0.303	-0.216	-0.623	-1.14	-1.83	-2.14
840	0.892	0.305	-0.22	-0.627	-1.14	-1.85	-2.15
850	0.889	0.308	-0.223	-0.631	-1.14	-1.86	-2.17
860	0.885	0.311	-0.225	-0.635	-1.14	-1.88	-2.19
870	0.882	0.313	-0.228	-0.639	-1.14	-1.89	-2.21
880	0.878	0.315	-0.231	-0.643	-1.14	-1.91	-2.22
890	0.874	0.318	-0.234	-0.647	-1.14	-1.92	-2.24
900	0.869	0.32	-0.237	-0.65	-1.15	-1.94	-2.26
910	0.865	0.323	-0.239	-0.654	-1.15	-1.95	-2.28
920	0.861	0.325	-0.242	-0.658	-1.15	-1.97	-2.29
930	0.856	0.327	-0.245	-0.661	-1.15	-1.98	-2.31
940	0.851	0.33	-0.247	-0.664	-1.15	-2	-2.33
950	0.847	0.332	-0.25	-0.668	-1.15	-2.01	-2.34
960	0.842	0.334	-0.252	-0.671	-1.15	-2.03	-2.36
970	0.837	0.336	-0.254	-0.674	-1.15	-2.05	-2.37
980	0.832	0.339	-0.257	-0.677	-1.15	-2.06	-2.39
990	0.826	0.341	-0.259	-0.68	-1.15	-2.08	-2.4
1000	0.821	0.343	-0.261	-0.683	-1.15	-2.09	-2.42
1010	0.816	0.345	-0.263	-0.686	-1.15	-2.11	-2.44
1020	0.81	0.347	-0.265	-0.689	-1.15	-2.13	-2.45
1030	0.805	0.349	-0.267	-0.691	-1.15	-2.14	-2.46

1040	0.799	0.351	-0.269	-0.694	-1.15	-2.16	-2.48
1050	0.793	0.353	-0.27	-0.696	-1.15	-2.18	-2.49
1060	0.787	0.355	-0.272	-0.699	-1.15	-2.19	-2.51
1070	0.782	0.357	-0.274	-0.701	-1.15	-2.21	-2.52
1080	0.776	0.359	-0.275	-0.703	-1.15	-2.23	-2.54
1090	0.77	0.361	-0.276	-0.706	-1.15	-2.25	-2.55
1100	0.764	0.363	-0.278	-0.708	-1.15	-2.26	-2.56
1110	0.758	0.365	-0.279	-0.71	-1.15	-2.28	-2.58
1120	0.752	0.367	-0.28	-0.712	-1.15	-2.3	-2.59
1130	0.746	0.369	-0.281	-0.713	-1.15	-2.32	-2.6
1140	0.74	0.37	-0.282	-0.715	-1.15	-2.34	-2.62
1150	0.734	0.372	-0.283	-0.717	-1.15	-2.35	-2.63
1160	0.728	0.374	-0.283	-0.719	-1.15	-2.37	-2.64
1170	0.722	0.376	-0.284	-0.72	-1.15	-2.39	-2.65
1180	0.716	0.377	-0.285	-0.722	-1.15	-2.41	-2.66
1190	0.71	0.379	-0.285	-0.723	-1.15	-2.43	-2.68
1200	0.704	0.381	-0.285	-0.724	-1.15	-2.45	-2.69
1210	0.698	0.382	-0.285	-0.726	-1.15	-2.47	-2.7
1220	0.692	0.384	-0.285	-0.727	-1.15	-2.48	-2.71
1230	0.686	0.385	-0.285	-0.728	-1.15	-2.5	-2.72
1240	0.68	0.387	-0.285	-0.729	-1.15	-2.52	-2.73
1250	0.675	0.388	-0.285	-0.73	-1.15	-2.54	-2.74
1260	0.669	0.39	-0.285	-0.732	-1.15	-2.56	-2.75
1270	0.663	0.391	-0.284	-0.733	-1.15	-2.58	-2.76
1280	0.658	0.393	-0.283	-0.734	-1.15	-2.6	-2.78
1290	0.652	0.394	-0.283	-0.736	-1.15	-2.62	-2.79
1300	0.647	0.396	-0.282	-0.737	-1.15	-2.64	-2.8
1310	0.641	0.397	-0.281	-0.738	-1.15	-2.66	-2.8
1320	0.636	0.398	-0.279	-0.739	-1.15	-2.68	-2.81
1330	0.631	0.4	-0.278	-0.741	-1.15	-2.7	-2.82
1340	0.626	0.401	-0.277	-0.742	-1.15	-2.72	-2.83
1350	0.621	0.402	-0.275	-0.743	-1.15	-2.74	-2.84
1360	0.616	0.403	-0.273	-0.744	-1.15	-2.76	-2.85
1370	0.611	0.405	-0.271	-0.745	-1.15	-2.78	-2.86
1380	0.606	0.406	-0.269	-0.746	-1.15	-2.8	-2.87
1390	0.602	0.407	-0.267	-0.747	-1.15	-2.81	-2.88
1400	0.597	0.408	-0.265	-0.748	-1.15	-2.83	-2.88
1410	0.593	0.409	-0.262	-0.749	-1.15	-2.85	-2.89
1420	0.589	0.41	-0.26	-0.75	-1.15	-2.87	-2.9
1430	0.585	0.411	-0.257	-0.751	-1.15	-2.89	-2.91
1440	0.581	0.412	-0.254	-0.751	-1.15	-2.91	-2.91

1450	0.578	0.413	-0.251	-0.752	-1.15	-2.93	-2.92
1460	0.574	0.414	-0.248	-0.753	-1.15	-2.94	-2.93
1470	0.571	0.415	-0.244	-0.754	-1.15	-2.96	-2.94
1480	0.568	0.416	-0.241	-0.754	-1.15	-2.98	-2.94
1490	0.565	0.417	-0.237	-0.755	-1.15	-2.99	-2.95
1500	0.562	0.418	-0.233	-0.756	-1.15	-3.01	-2.96

References

S1) Kean, Z. S.; Akbulatov, S.; Tian, Y.; Widenhoefer, R. A.; Boulatov, R.; Craig, S. L. Photomechanical actuation of ligand geometry in enantioselective catalysis. *Angew. Chem., Int. Ed*. **2014**, *53*, 14508.

S2) L. Wang, Y. Yu, A. O. Razgoniaev, P. N. Johnson, C. Wang, Y. Tian, R. Boulatov, S. L. Craig and R. A. Widenhoefer, *J. Am. Chem. Soc.*, **2020**, *142*, 17714-17720.

S3) Scrivanti A.; Zeggio S.; Beghetto V.; Matteoli U. Asymmetric hydroformylation of styrene with $PtCl_2$(atropoisomeric diphosphine)$SnCl_2$ systems *J. Mol. Cat. A: Chemical* **1995**, *101*, 217.

S4) Momeni, B. Z.; Kazmi, H.; Najafi, A. Tin(II) Halide Insertion or Halogen Exchange in the Reactions of Dihaloplatinum(II) Complexes with Tin(II) Halide. *Helv. Chim. Acta* **2011**, *94*, 1618.

S5) Frisch, M. J.; Trucks, G. W.; Schlegel, H. B.; Scuseria, G. E.; Robb, M. A.; Cheeseman, J. R.; Scalmani, G.; Barone, V.; Mennucci, B.; Petersson, G. A.; Nakatsuji, H.; Caricato, M.; Li, X.; Hratchian, H. P.; Izmaylov, A. F.; Bloino, J.; Zheng, G.; Sonnenberg, J. L.; Hada, M.; Ehara, M.; Toyota, K.; Fukuda, R.; Hasegawa, J.; Ishida, M.; Nakajima, T.; Honda, Y.; Kitao, O.; Nakai, H.; Vreven, T.; Montgomery, J. A., Jr.; Peralta, J. E.; Ogliaro, F.; Bearpark, M.; Heyd, J. J.; Brothers, E.; Kudin, K. N.; Staroverov, V. N.; Kobayashi, R.; Normand, J.; Raghavachari, K.; Rendell, A.; Burant, J. C.; Iyengar, S. S.; Tomasi, J.; Cossi, M.; Rega, N.; Millam, M. J.; Klene, M.; Knox, J. E.; Cross, J. B.; Bakken, V.; Adamo, C.; Jaramillo, J.; Gomperts, R.; Stratmann, R. E.; Yazyev, O.; Austin, A. J.; Cammi, R.; Pomelli, C.; Ochterski, J. W.; Martin, R. L.; Morokuma, K.; Zakrzewski, V. G.; Voth, G. A.; Salvador, P.; Dannenberg, J. J.; Dapprich, S.; Daniels, A. D.; Farkas, Ö.; Foresman, J. B.; Ortiz, J. V.; Cioslowski, J.; Fox, D. J. Gaussian, Inc., Wallingford CT, **2009**.

S6) (a) Hay P.J.; Wadt W.R., Ab initio effective core potentials for molecular calculations. Potentials for the transition metal atoms Sc to Hg. *J. Chem. Phys.* **1985**, *82*, 270; (b) Wadt W.R.; Hay P.J., Ab initio effective core potentials for molecular calculations. Potentials for main group elements Na to Bi. *J. Chem. Phys.* **1985**, *82*, 284; (c) Hay P.J.; Wadt W.R., Ab initio effective core potentials for molecular calculations. Potentials for K to Au including the outermost core orbitals. *J. Chem. Phys.* **1985**, *82*, 299.

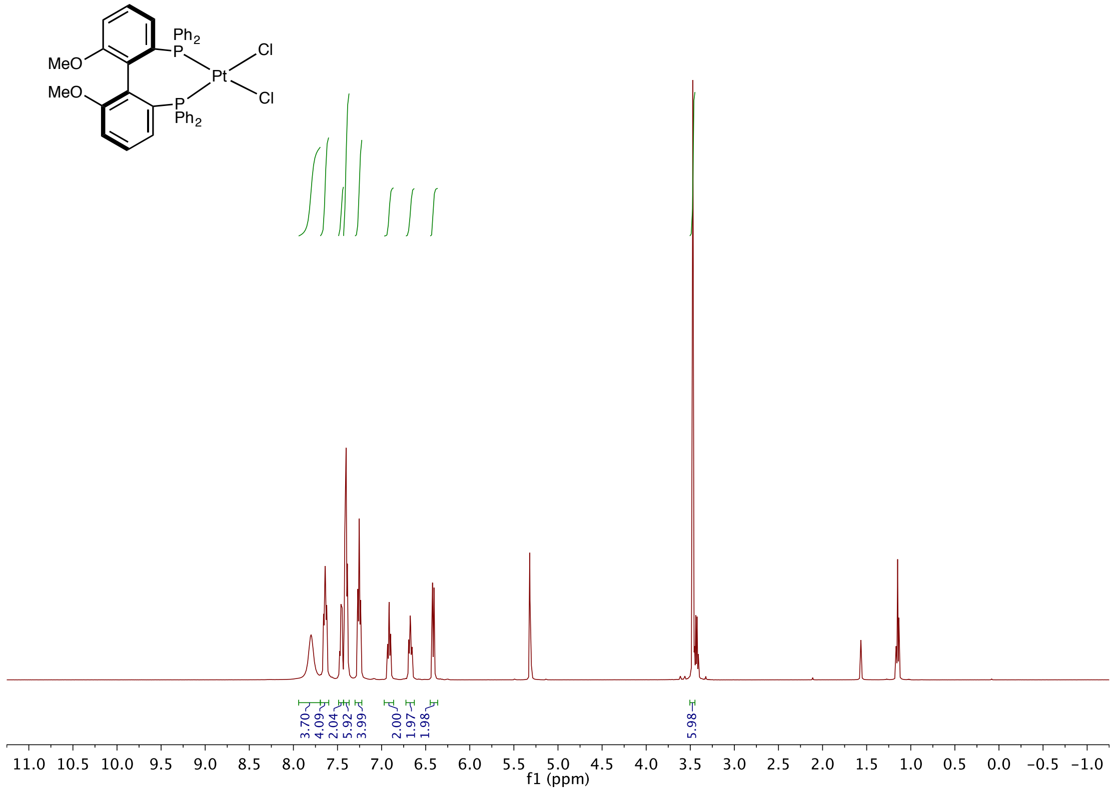

Figure S8. ^{1}H NMR spectrum of **(MeOBiphep)PtCl$_2$**.

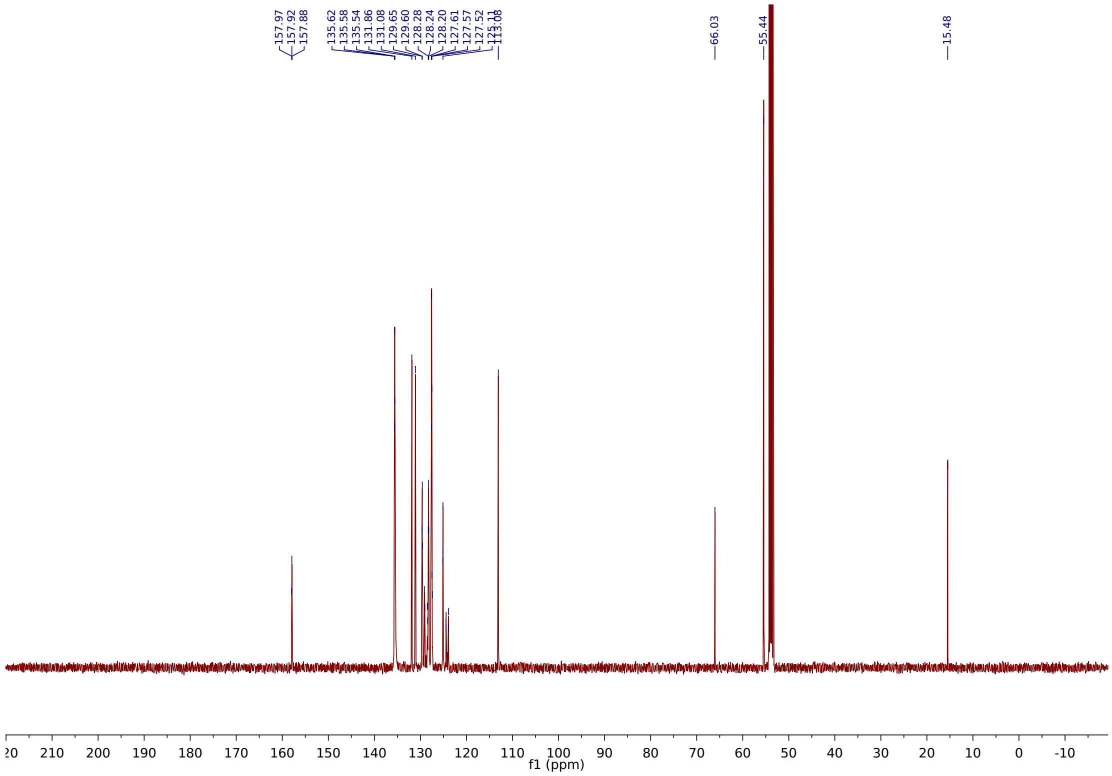

Figure S9. ^{13}C{^{1}H} NMR spectrum of **(MeOBiphep)PtCl$_2$**.

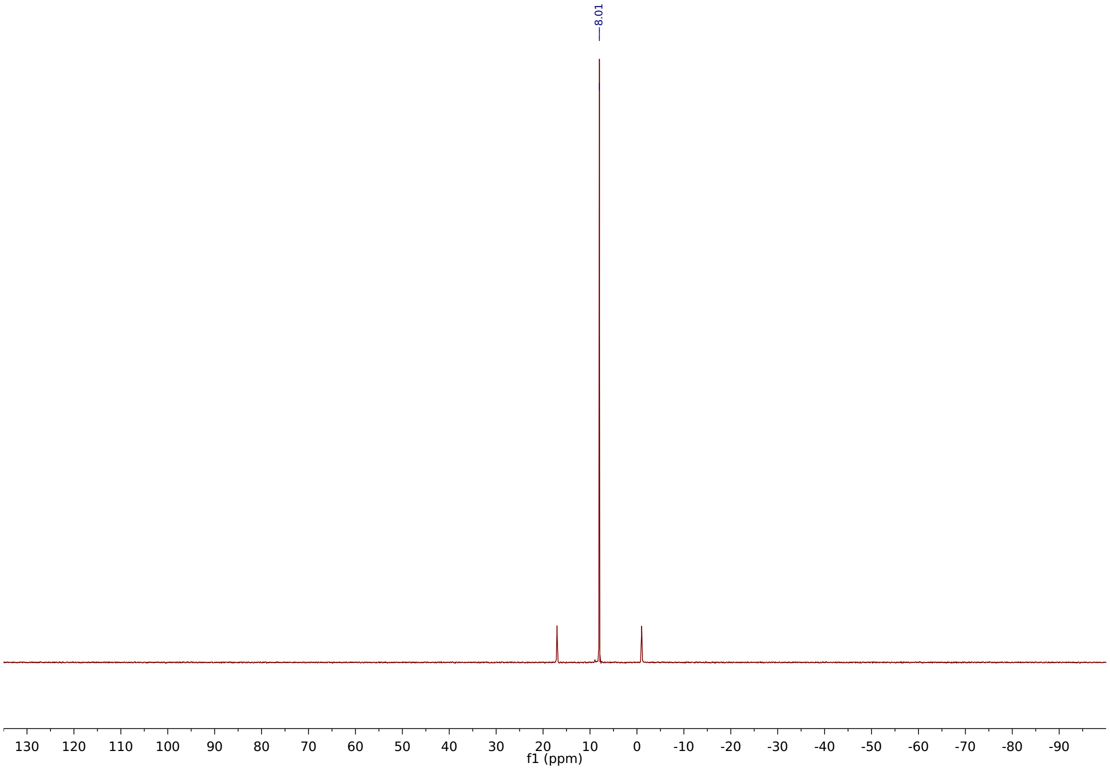

Figure S10. ^{31}P NMR spectrum of **(MeOBiphep)PtCl$_2$**.

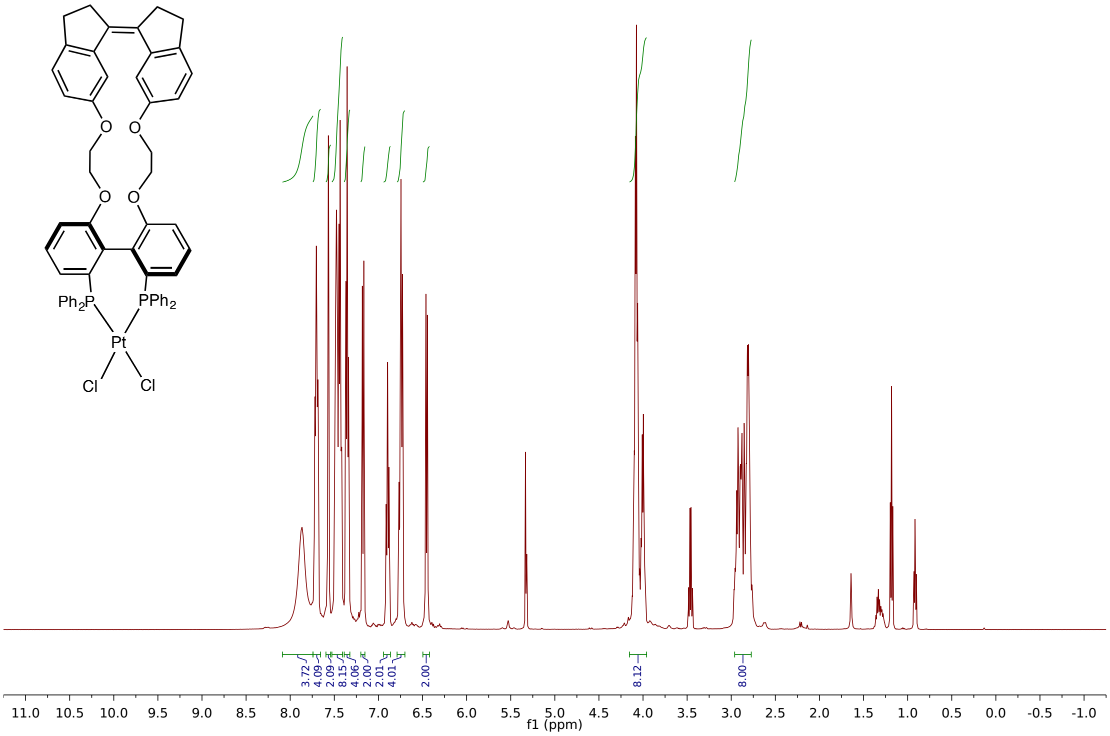

Figure S11. ^{1}H NMR spectrum of **[Z(2,2)]PtCl$_2$**.

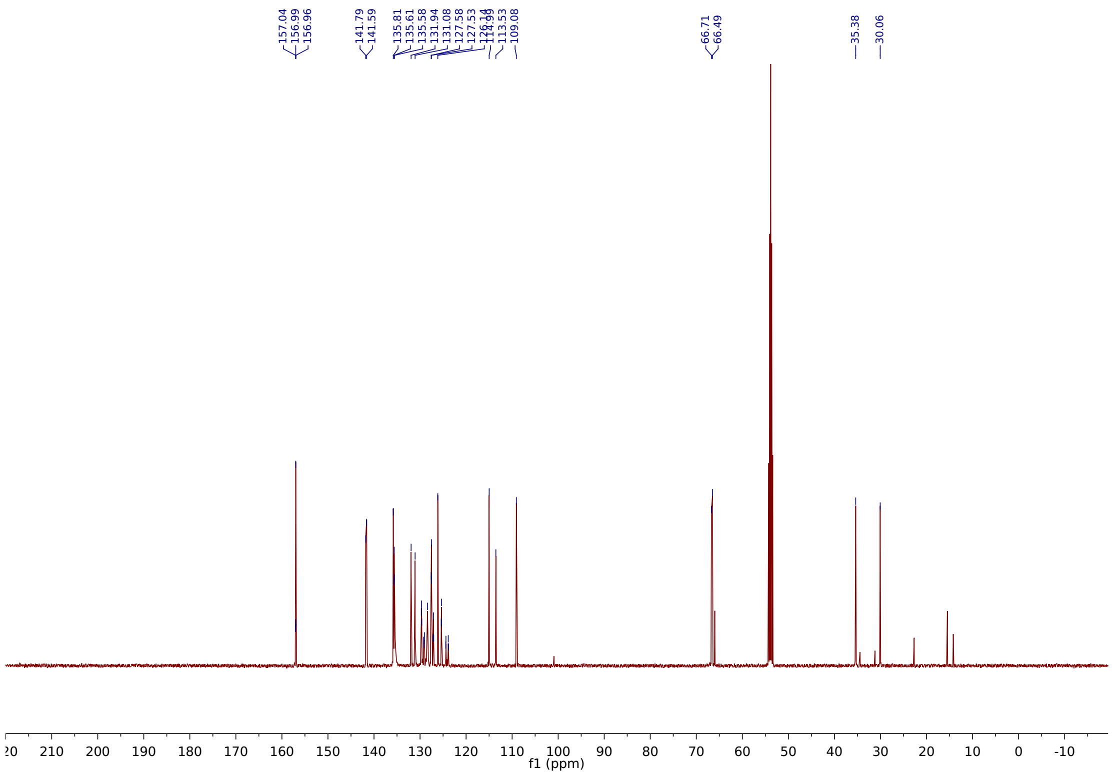

Figure S12. $^{13}C\{^{1}H\}$ NMR spectrum of **[Z(2,2)]PtCl₂**.

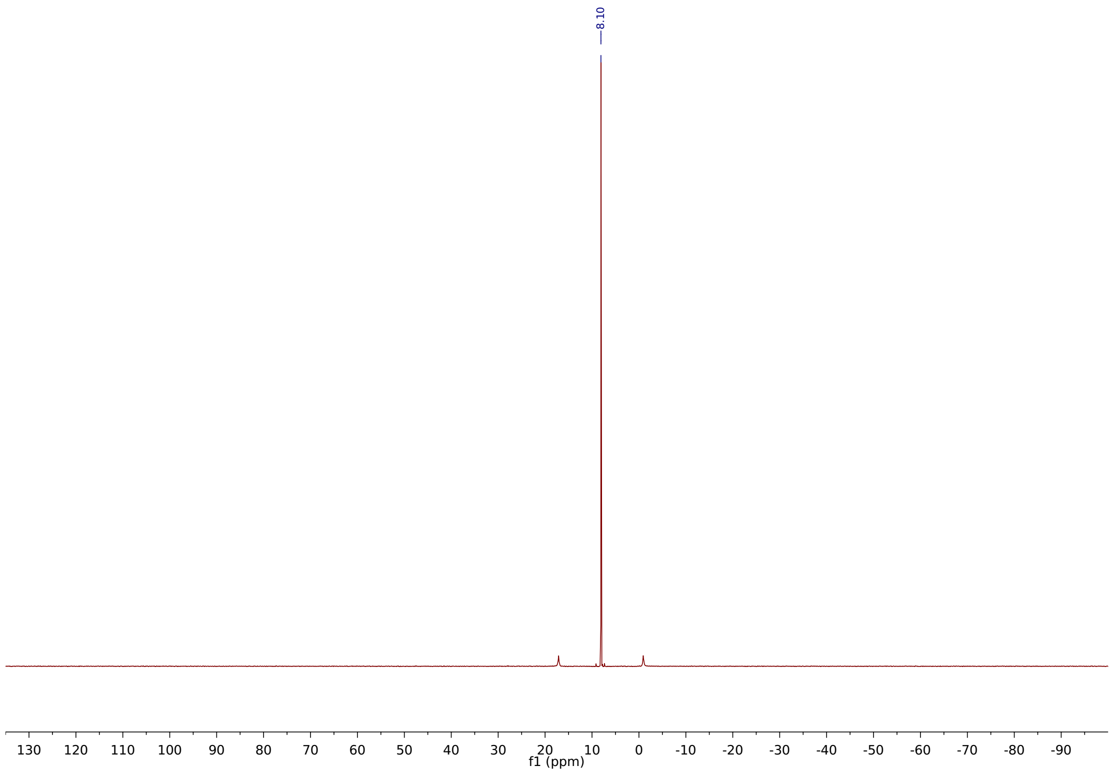

Figure S13. ^{31}P NMR spectrum of **[Z(2,2)]PtCl₂**.

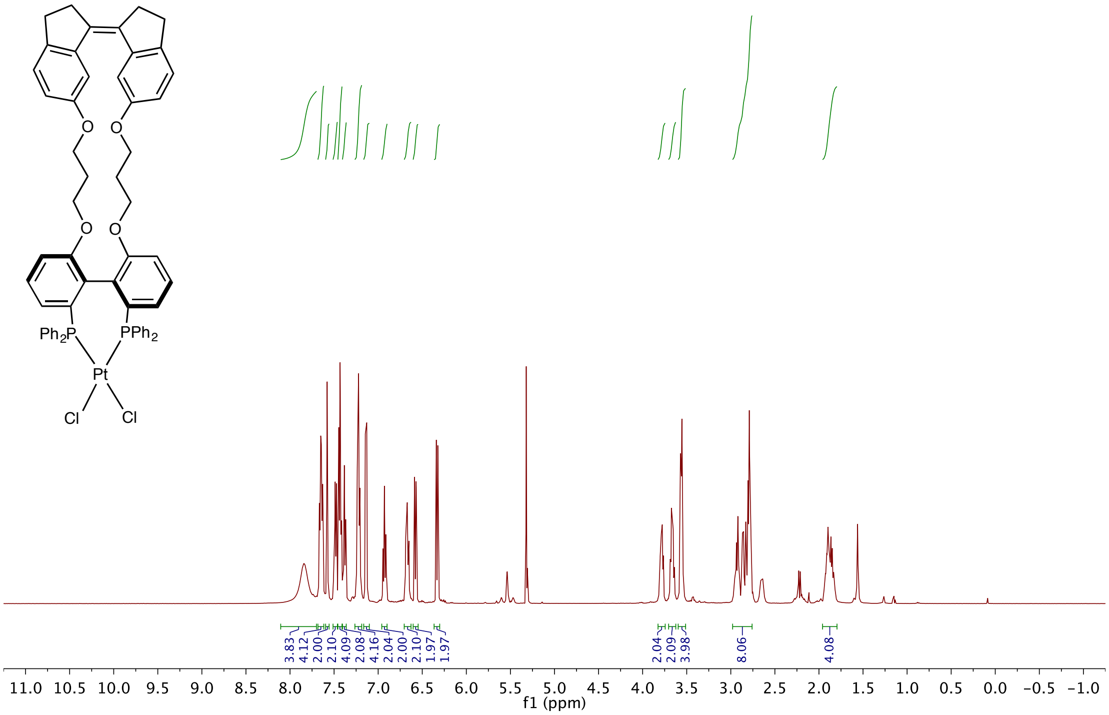

Figure S14. ^{1}H NMR spectrum of **[Z(3,3)]PtCl$_2$**.

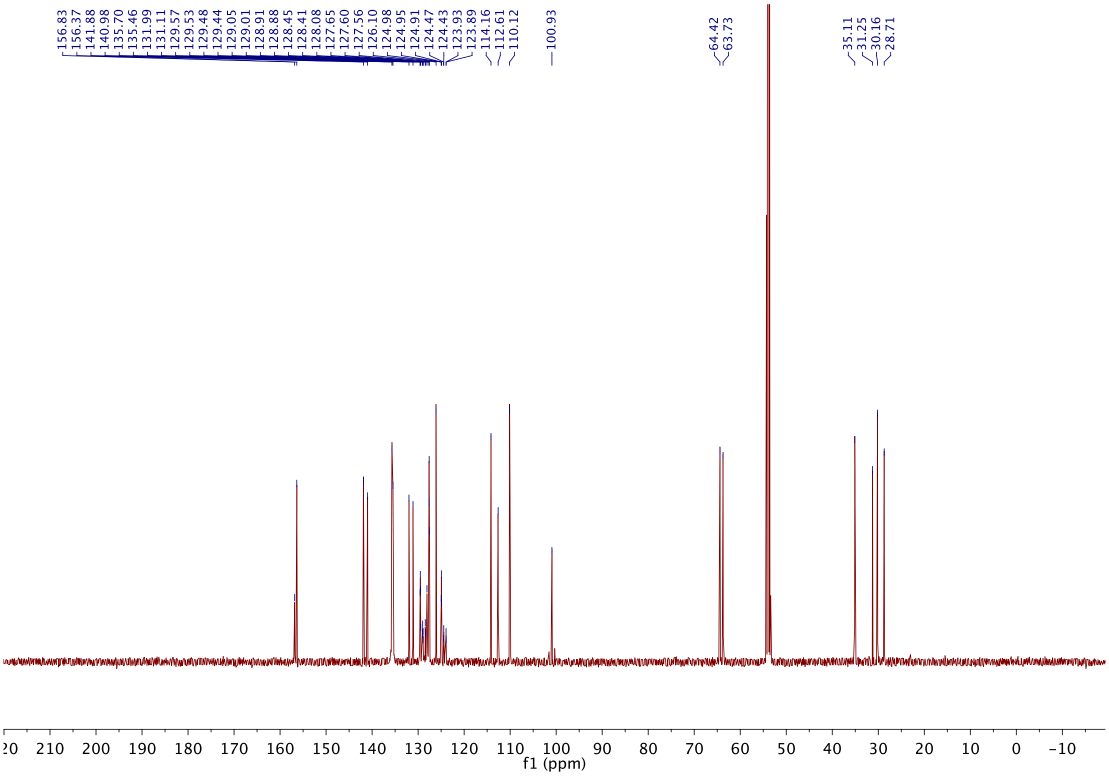

Figure S15. ^{13}C{^{1}H} NMR spectrum of **[Z(3,3)]PtCl$_2$**.

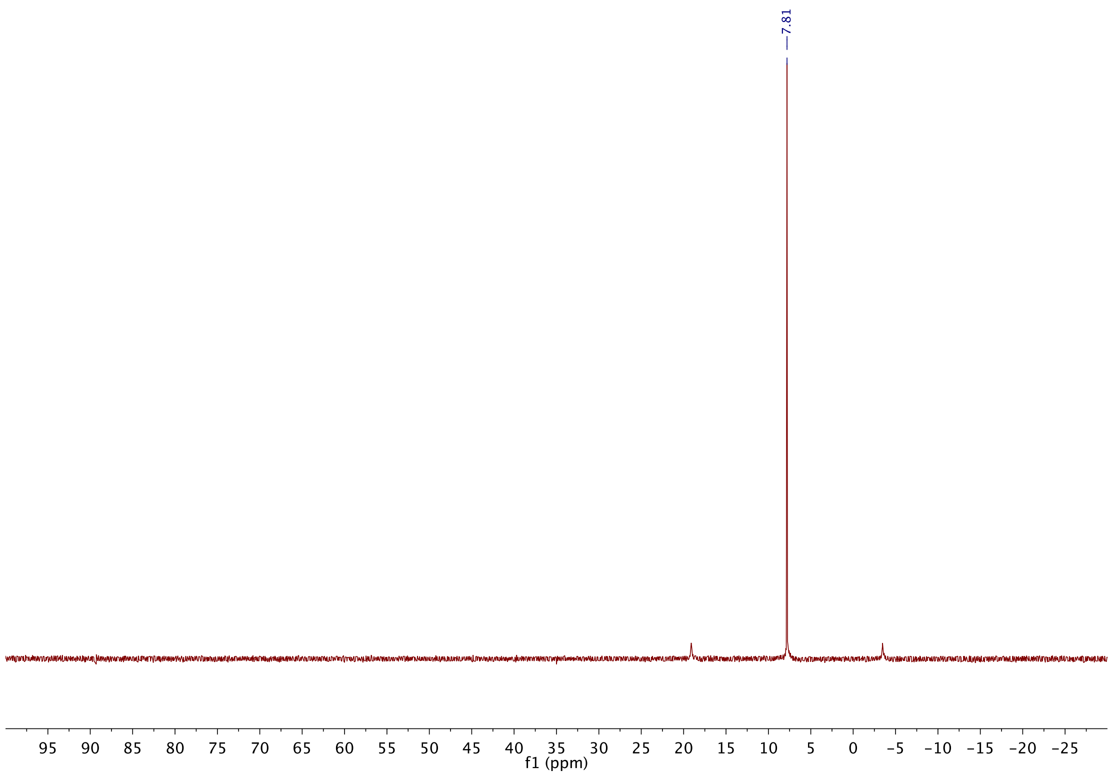

Figure S16. ^{31}P NMR spectrum of **[Z(3,3)]PtCl$_2$**.

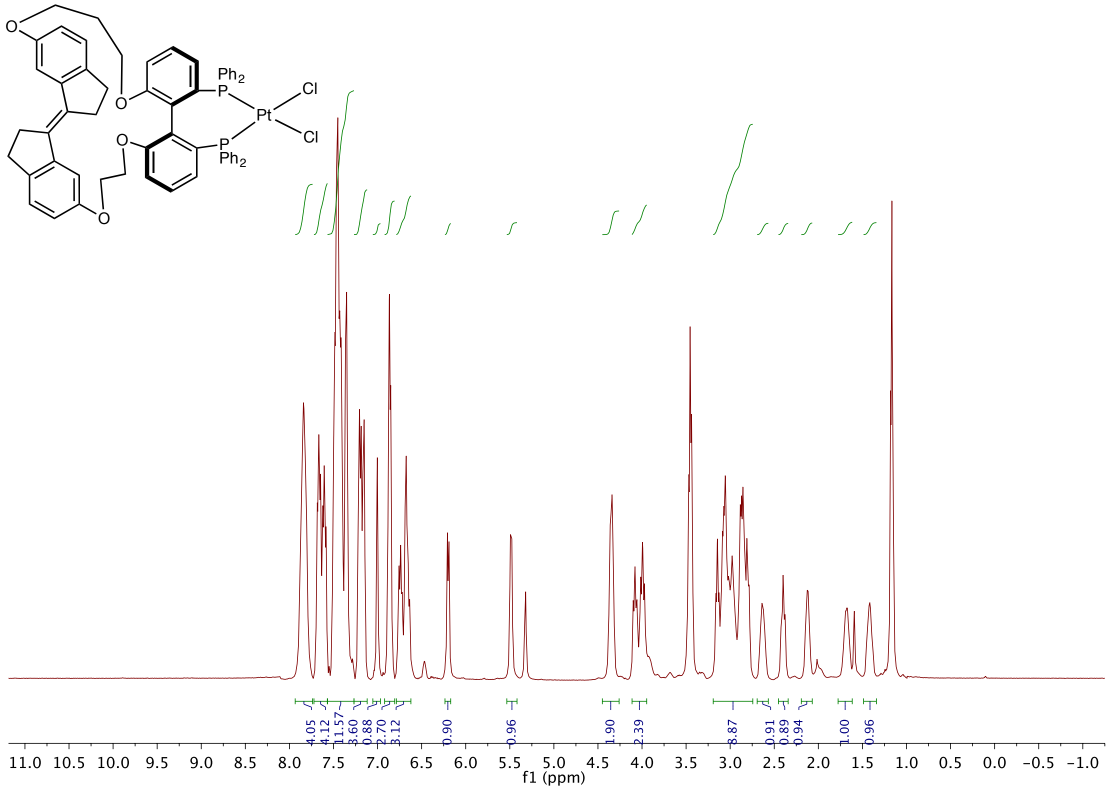

Figure S17. ^{1}H NMR spectrum of **[E(2,3)]PtCl$_2$**.

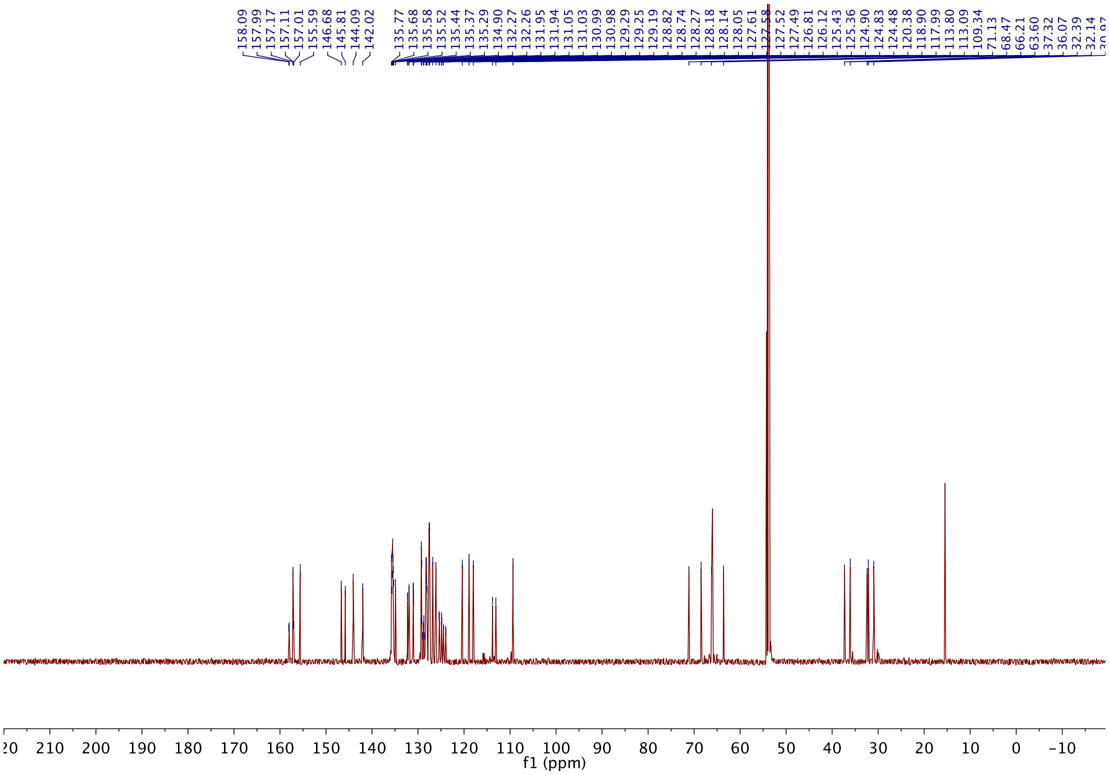

Figure S18. $^{13}C\{^{1}H\}$ NMR spectrum of **[E(2,3)]PtCl₂**.

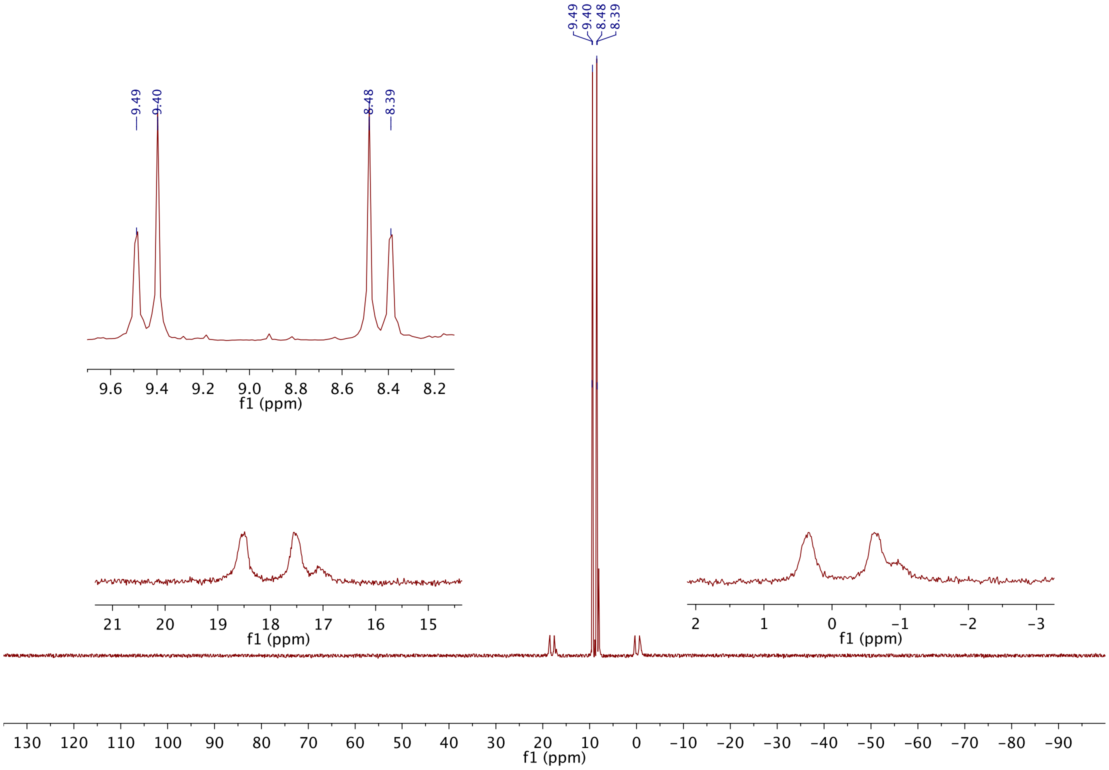

Figure S19. ^{31}P NMR spectrum of **[E(2,3)]PtCl₂**.

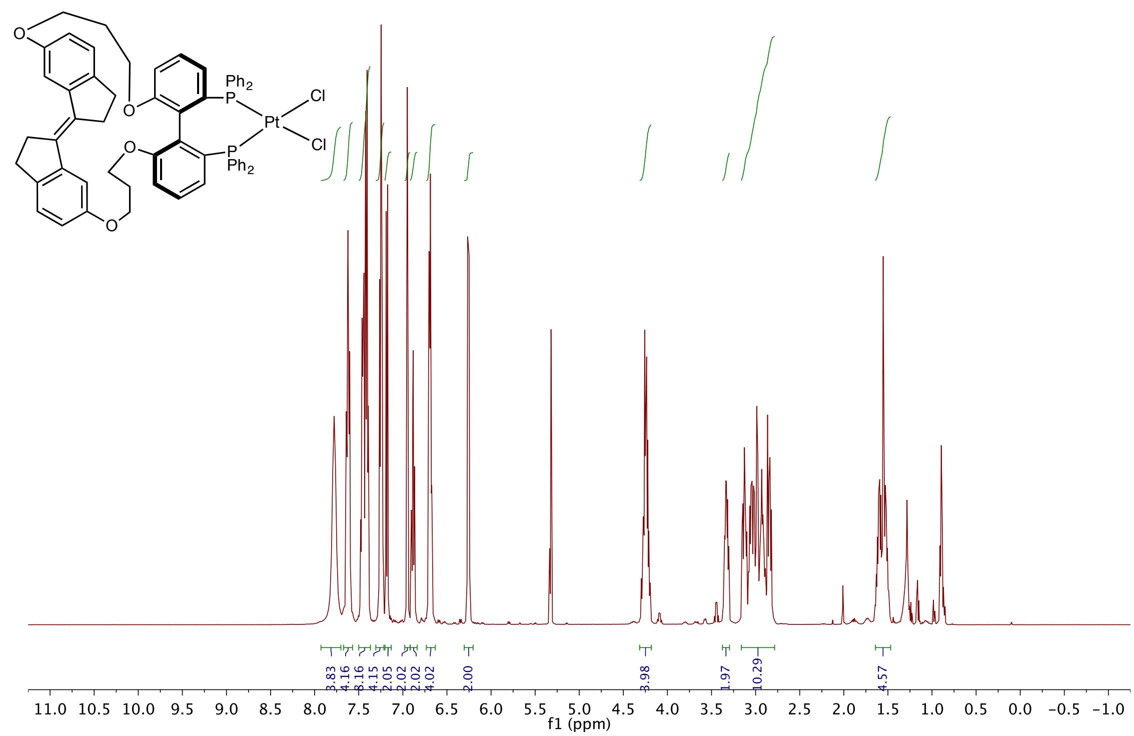

Figure S20. ^{1}H NMR spectrum of **[E(3,3)]PtCl$_2$**.

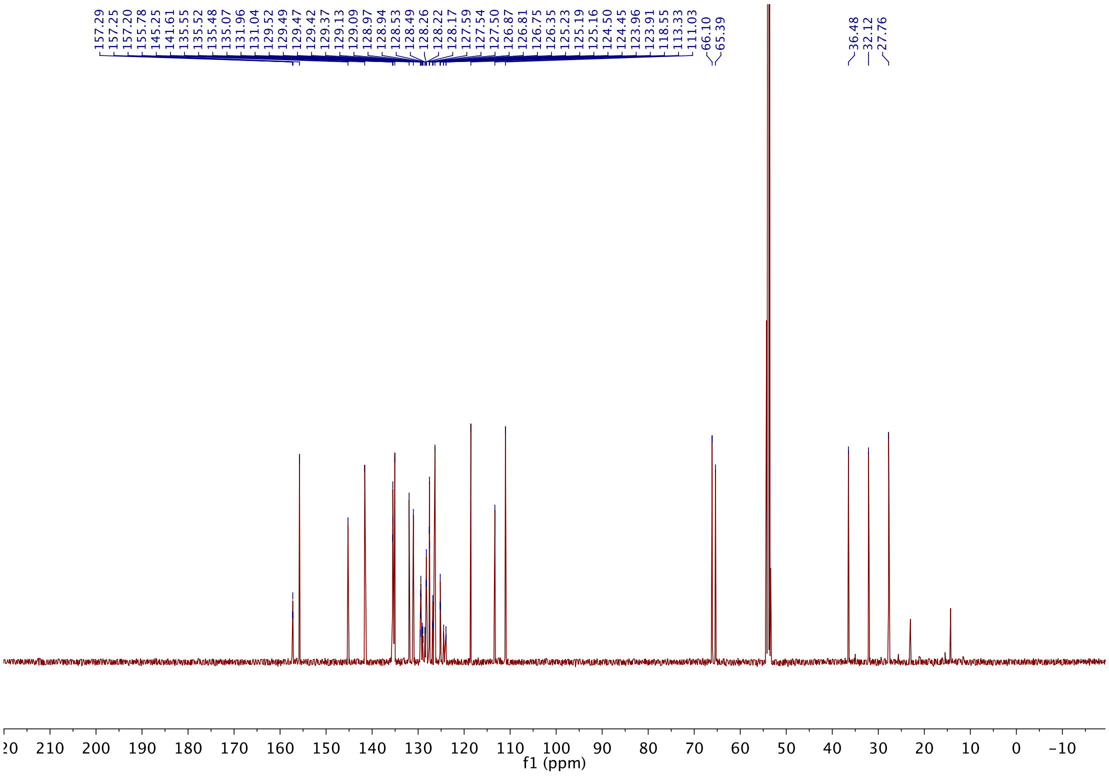

Figure S21. ^{13}C{^{1}H} NMR spectrum of **[E(3,3)]PtCl$_2$**.

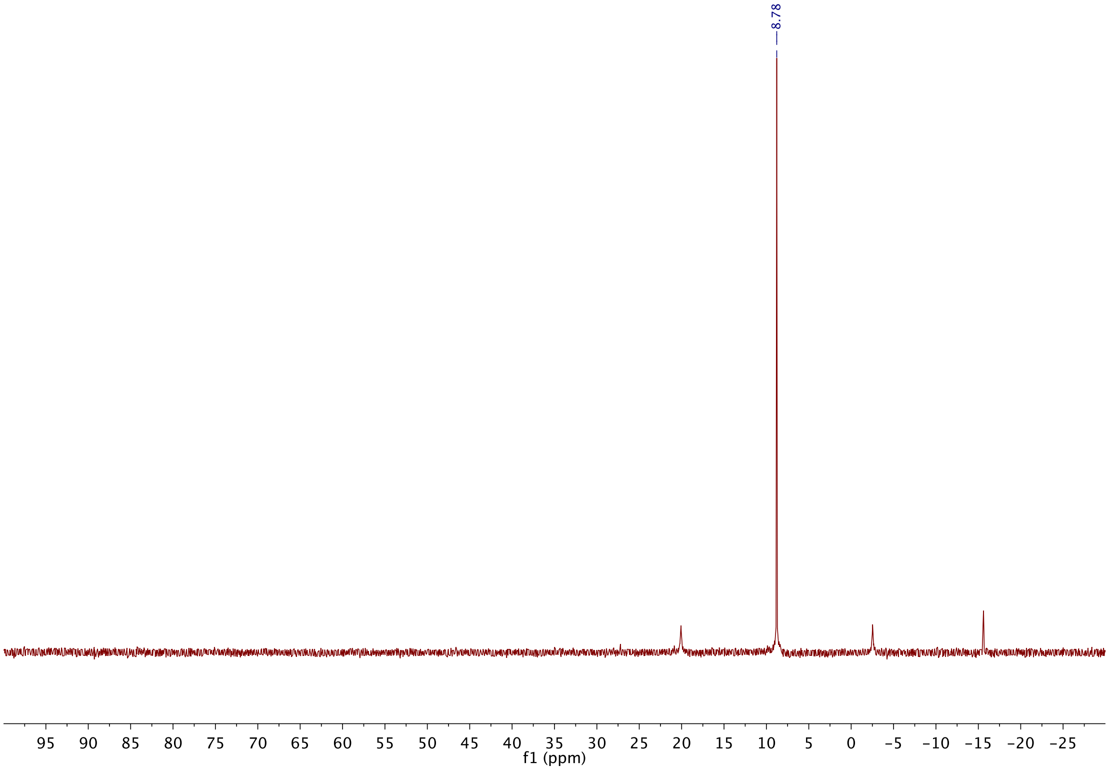

Figure S22. ^{31}P NMR spectrum of **[E(3,3)]PtCl$_2$**.

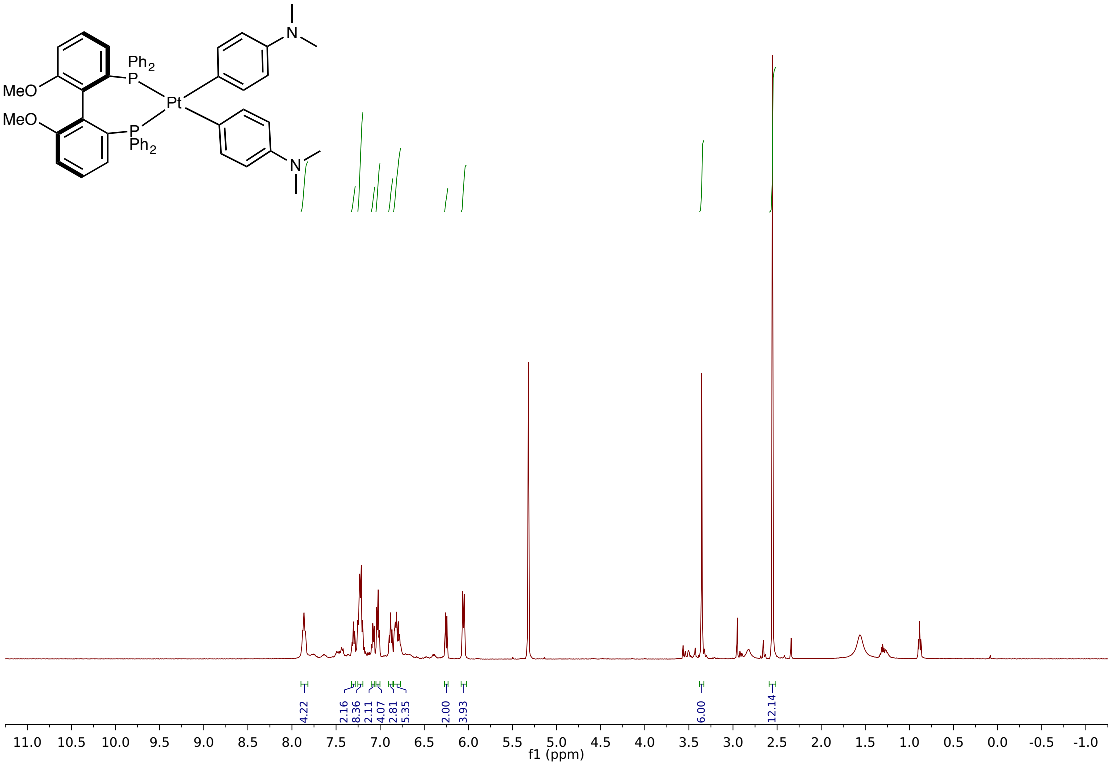

Figure S23. ^{1}H NMR spectrum of **(MeOBiphep)PtAr$_2$**.

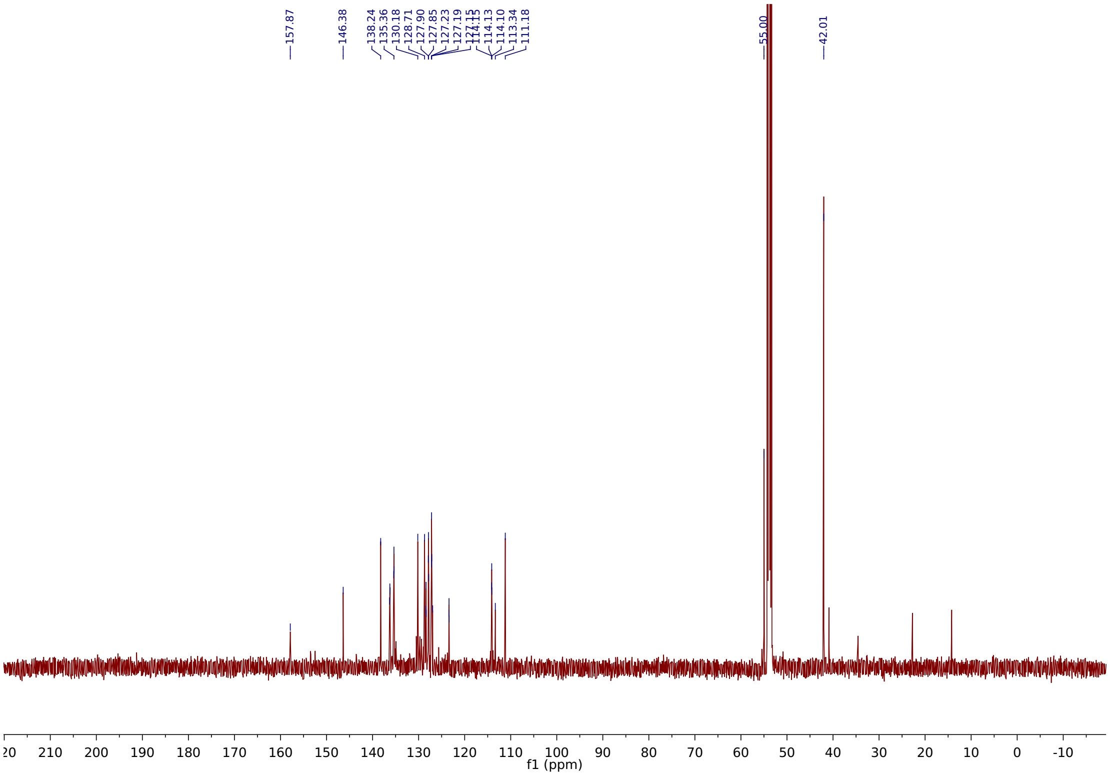

Figure S24. $^{13}C\{^1H\}$ NMR spectrum of **(MeOBiphep)PtAr$_2$**.

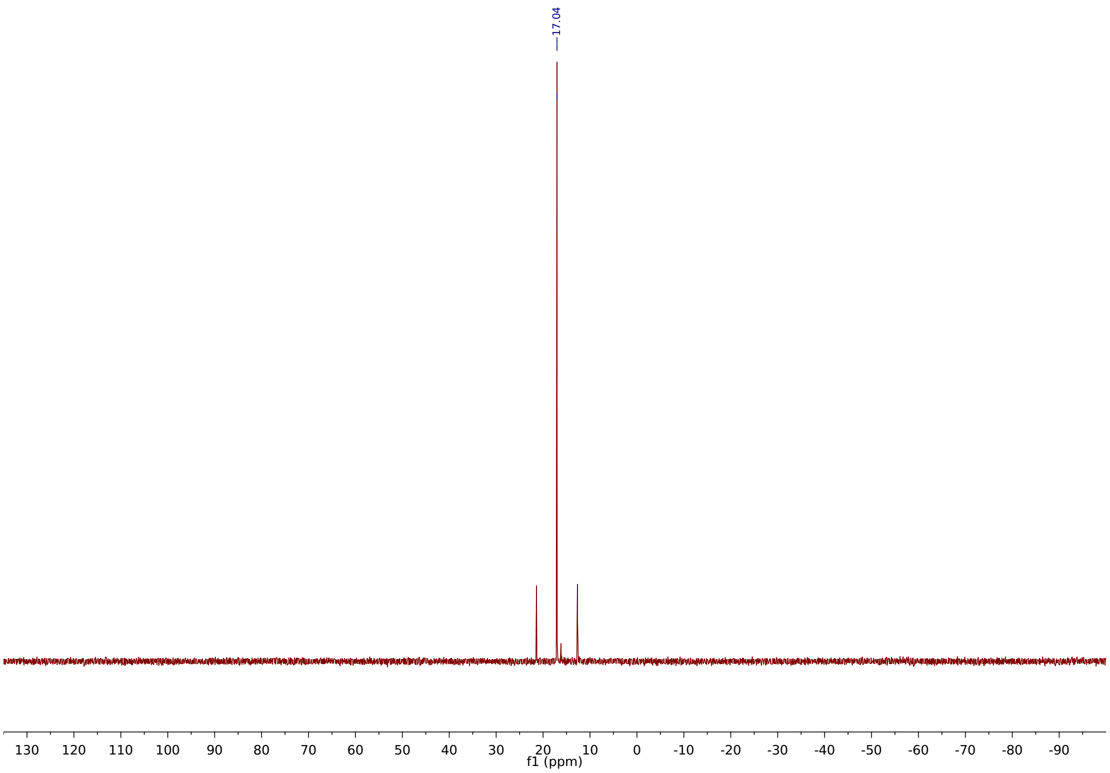

Figure S25. ^{31}P NMR spectrum of **(MeOBiphep)PtAr$_2$**.

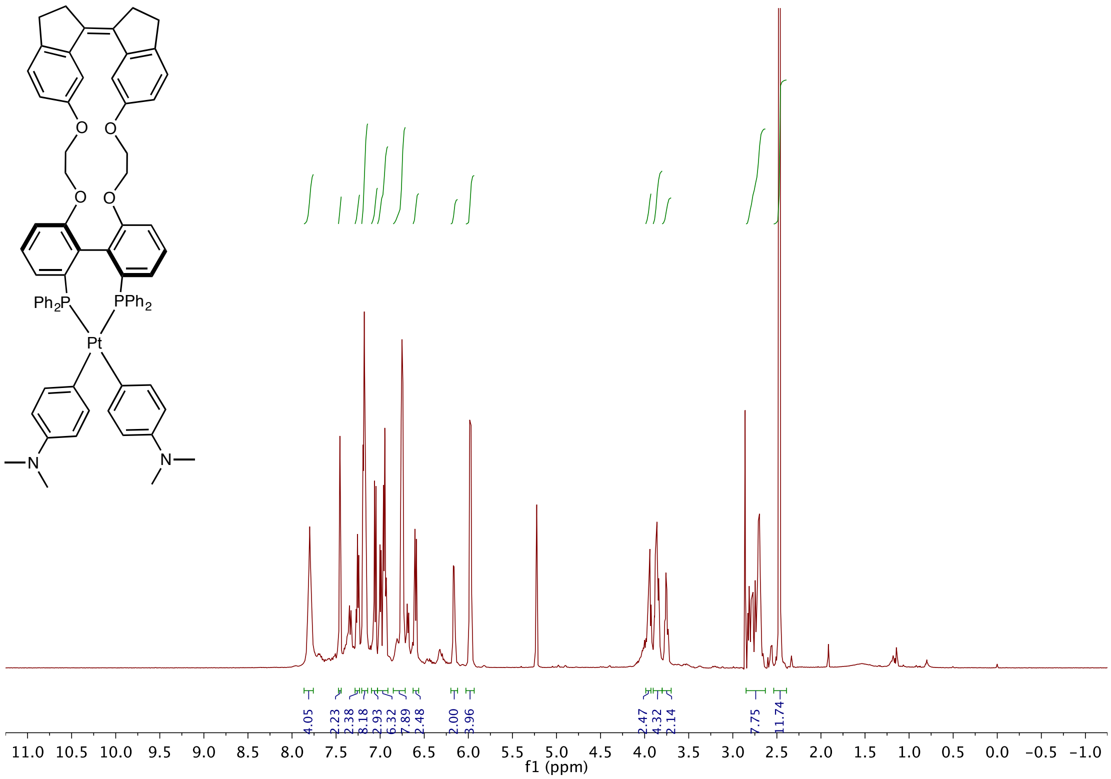

Figure S26. ^{1}H NMR spectrum of **[Z(2,2)]PtAr$_2$**.

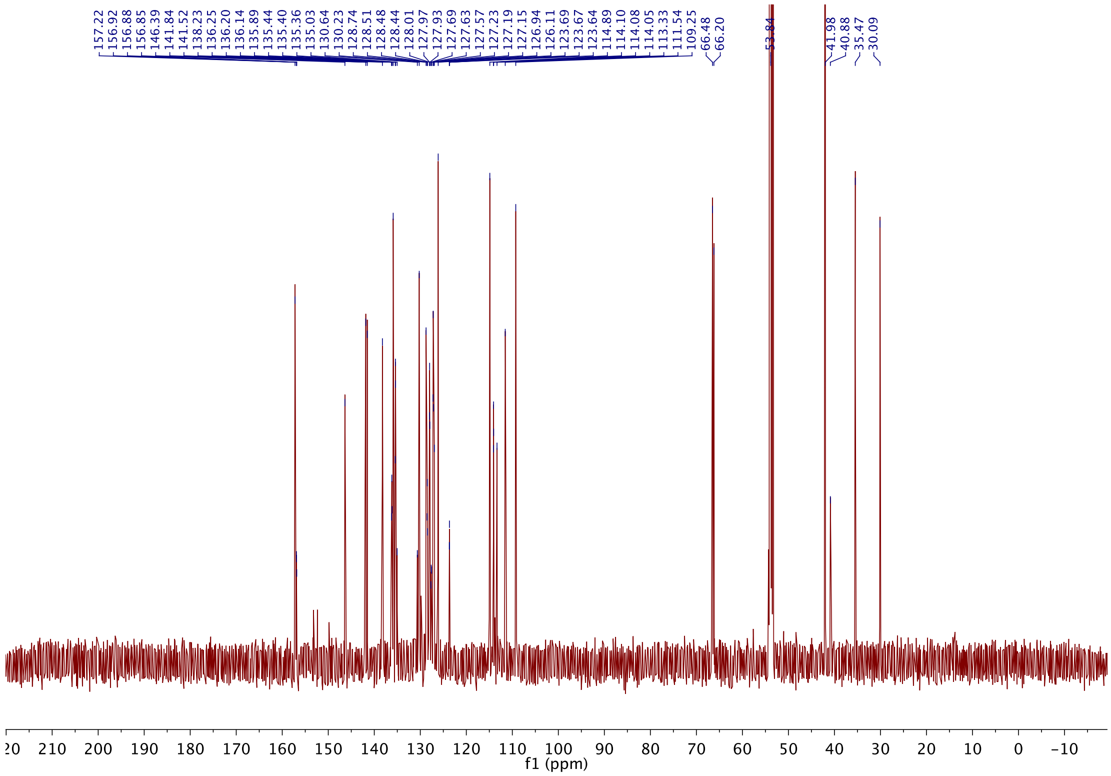

Figure S27. ^{13}C{^{1}H} NMR spectrum of **[Z(2,2)]PtAr$_2$**.

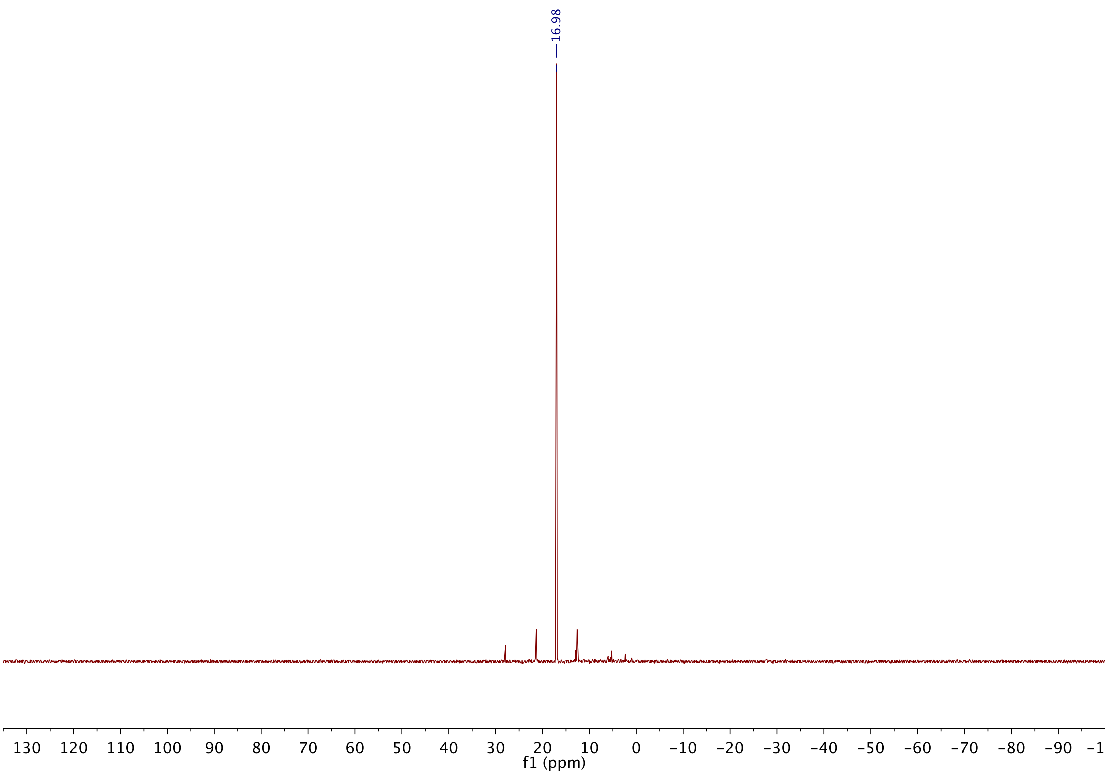

Figure S28. ^{31}P NMR spectrum of **[Z(2,2)]PtAr$_2$**.

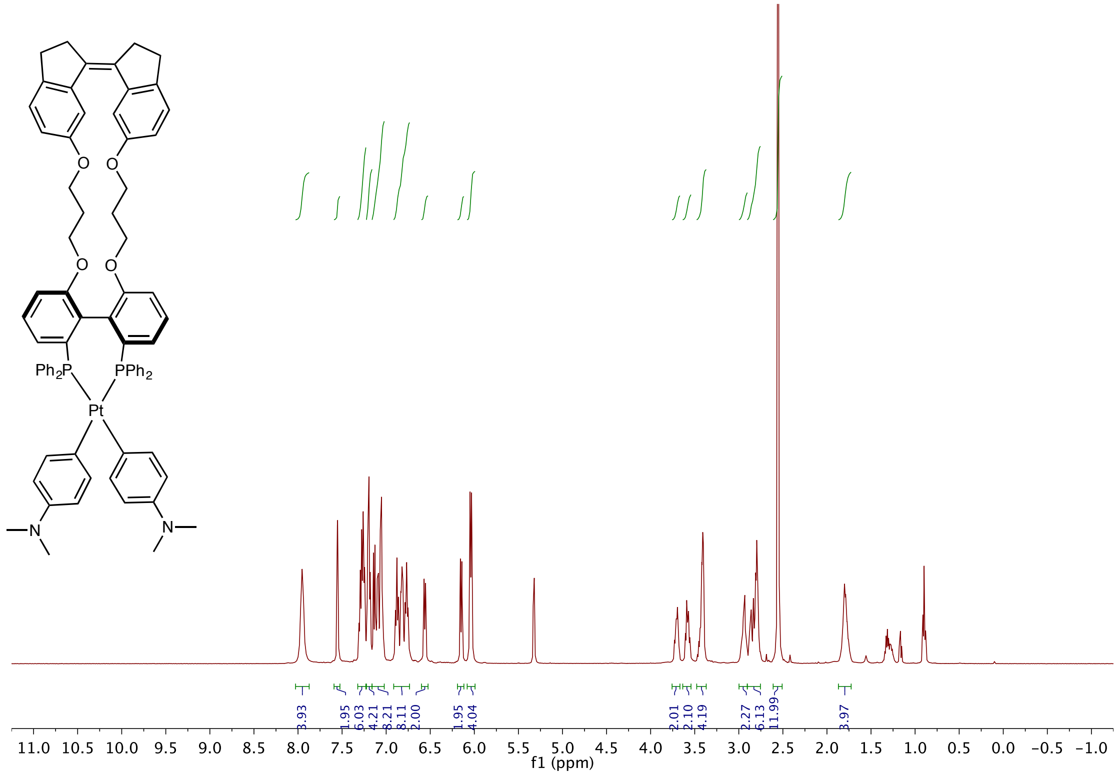

Figure S29. ^{1}H NMR spectrum of **[Z(3,3)]PtAr$_2$**.

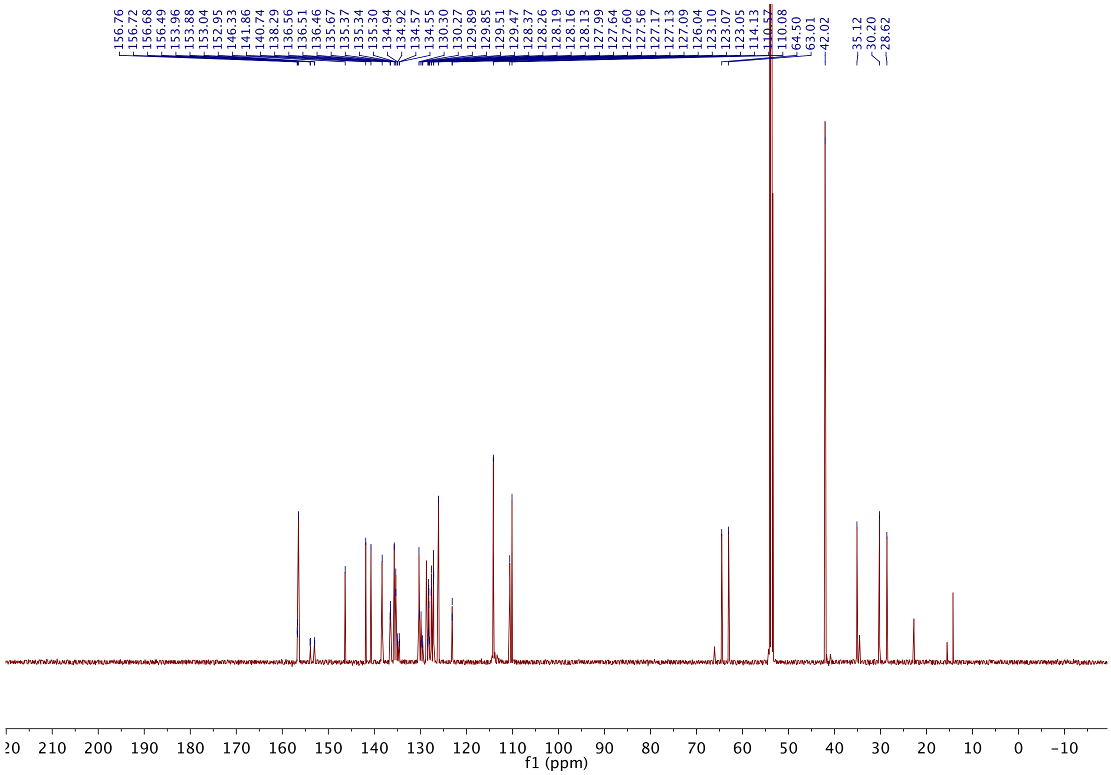

Figure S30. $^{13}C\{^{1}H\}$ NMR spectrum of **[Z(3,3)]PtAr₂**.

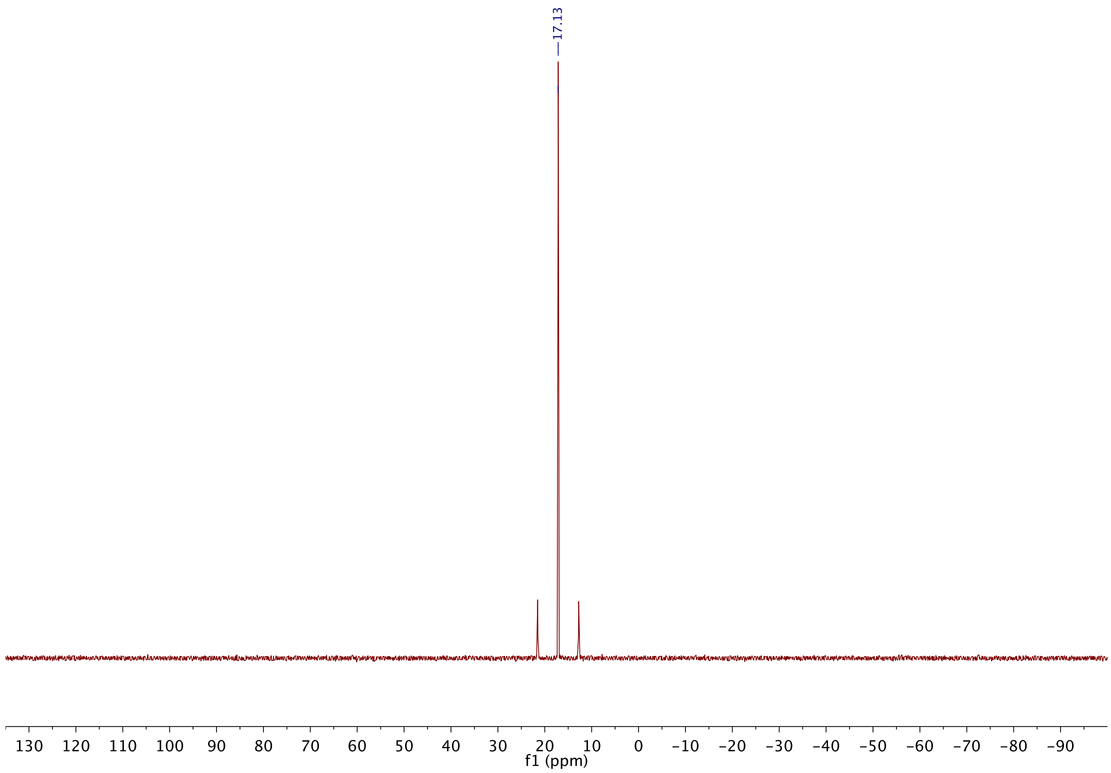

Figure S31. ^{31}P NMR spectrum of **[Z(3,3)]PtAr₂**.

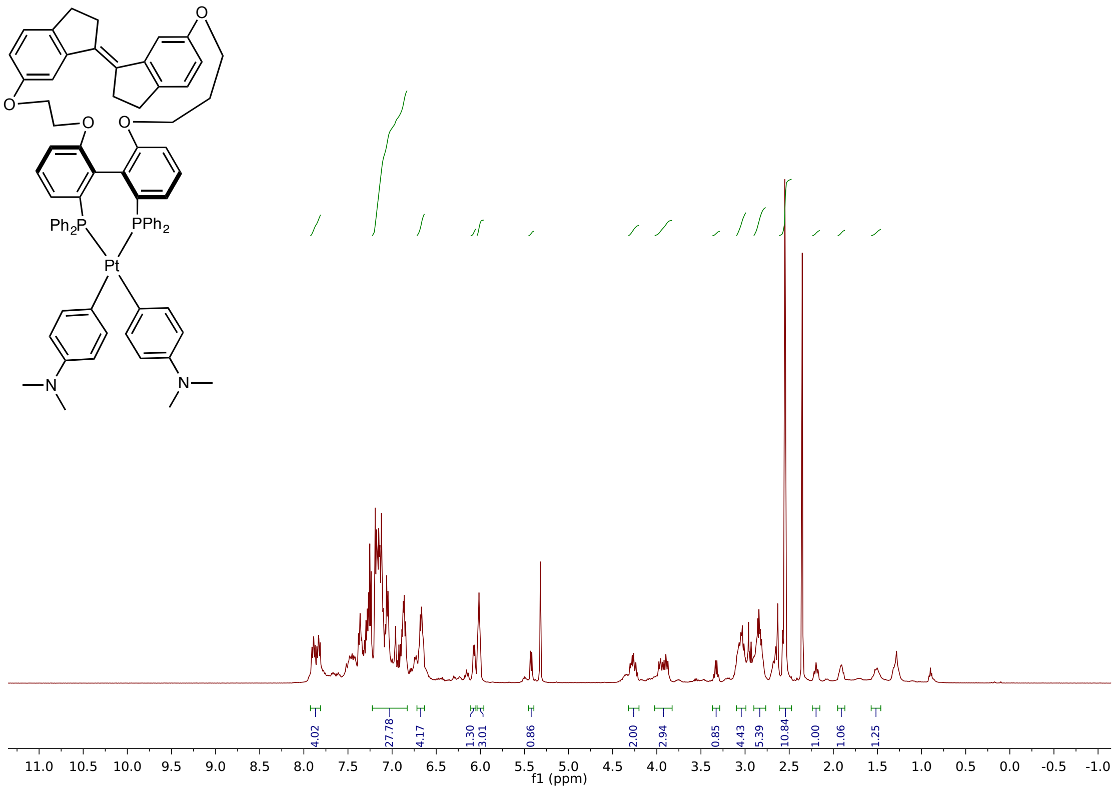

Figure S32. ^{1}H NMR spectrum of **[E(2,3)]PtAr$_2$**.

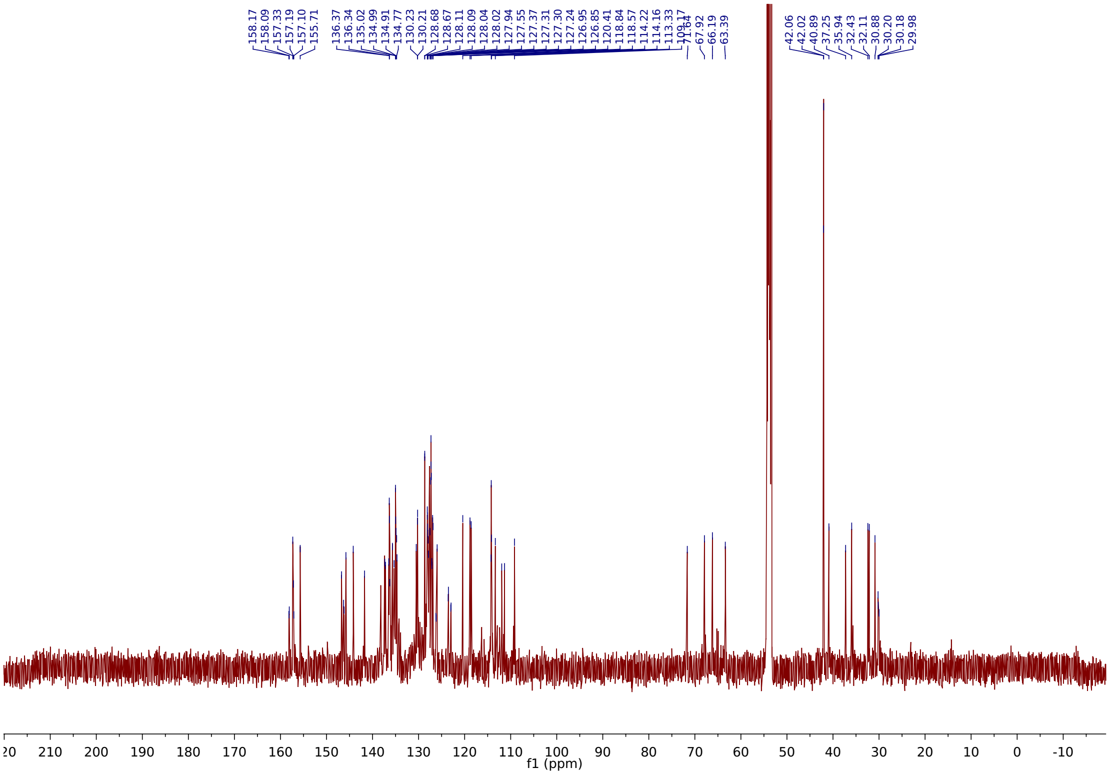

Figure S33. $^{13}C\{^{1}H\}$ NMR spectrum of **[E(2,3)]PtAr$_2$**.

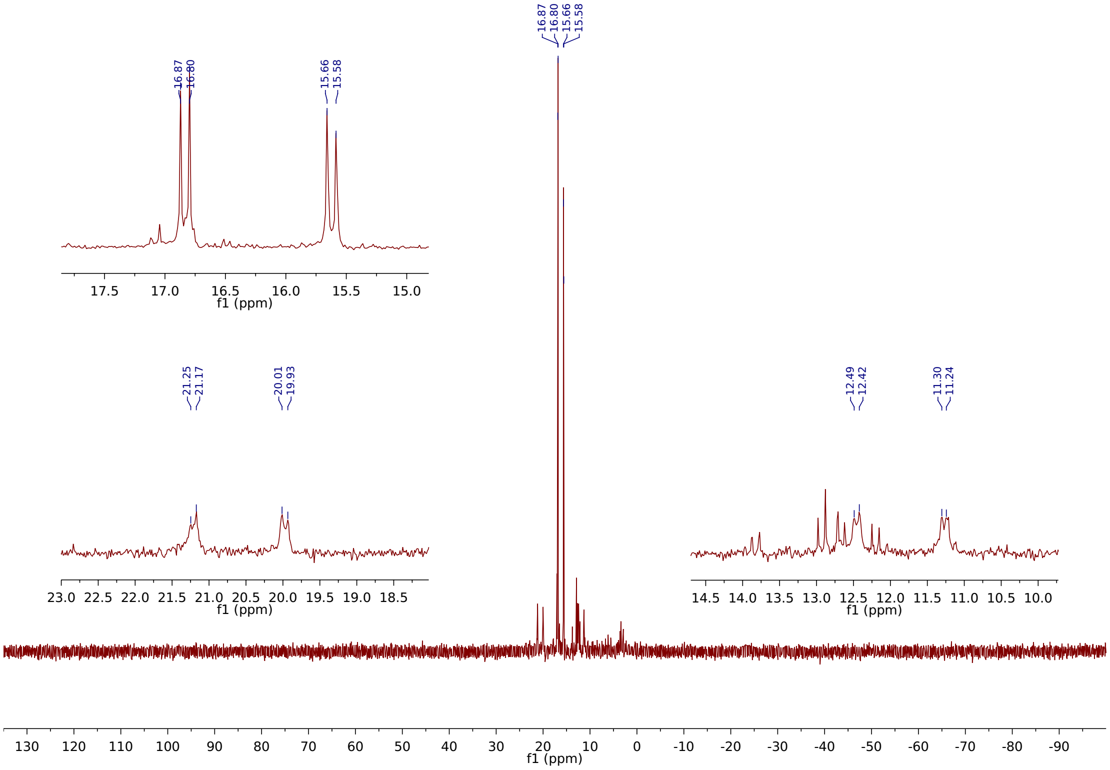

Figure S34. ^{31}P NMR spectrum of **[E(2,3)]PtAr$_2$**.

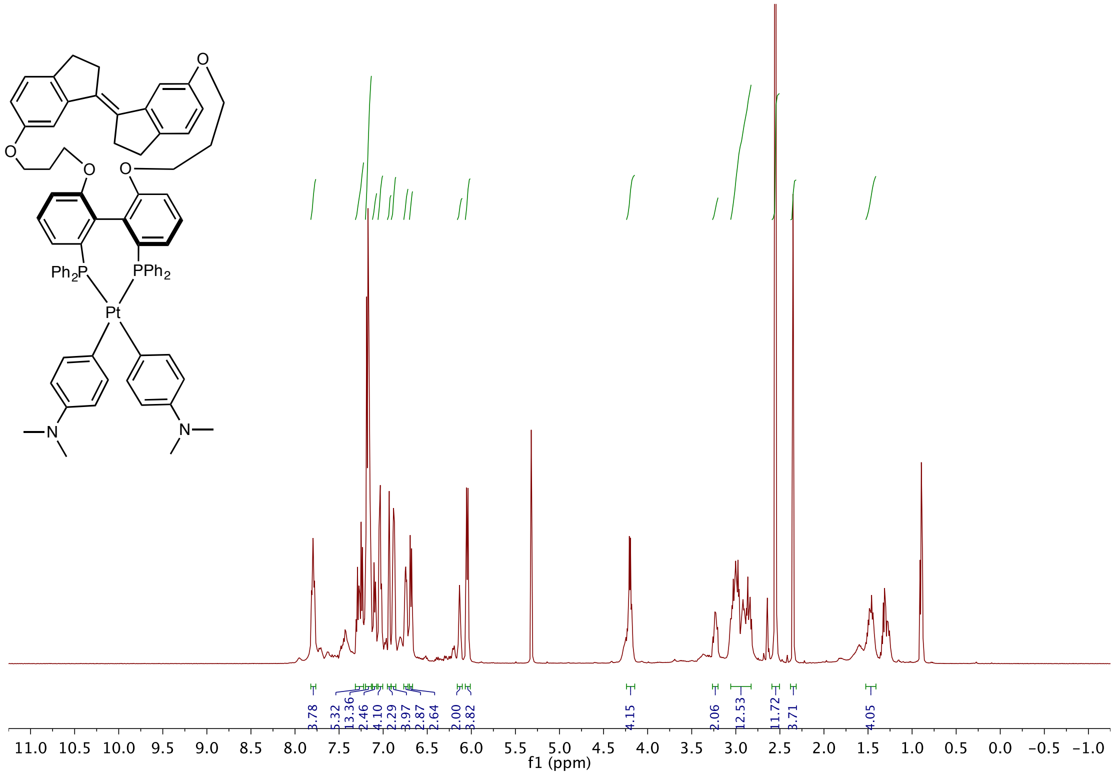

Figure S35. ^{1}H NMR spectrum of **[E(3,3)]PtAr$_2$**.

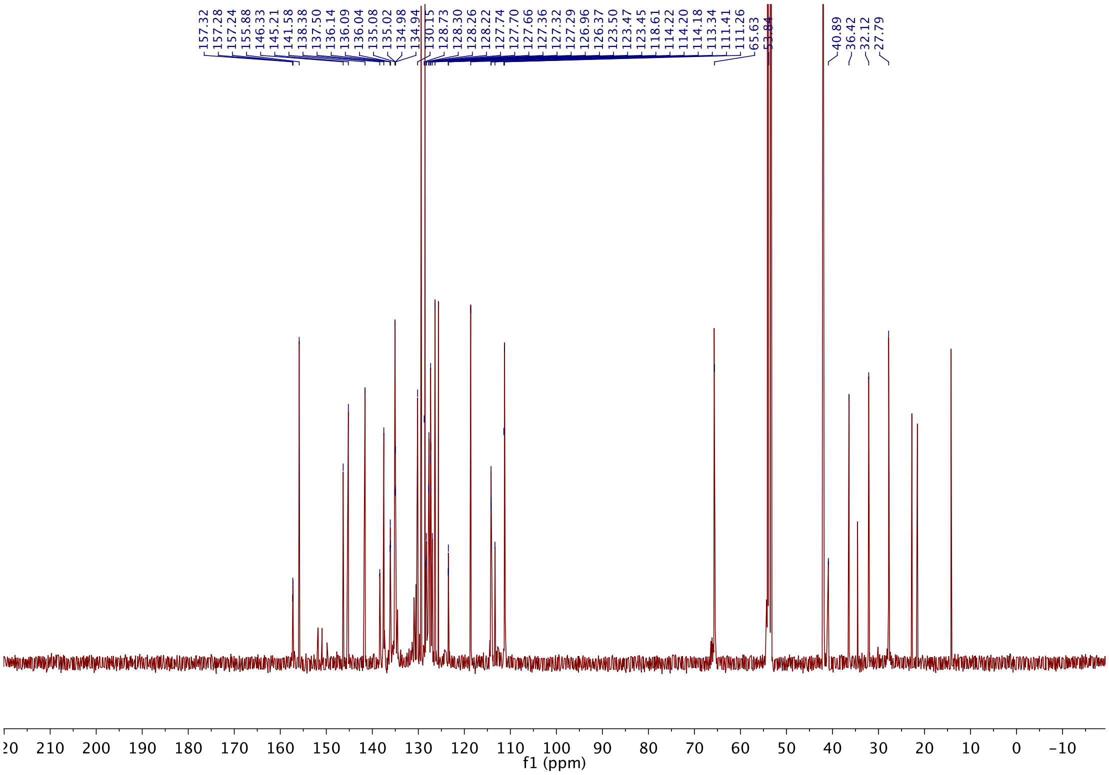

Figure S36. $^{13}C\{^{1}H\}$ NMR spectrum of **[E(3,3)]PtAr₂**.

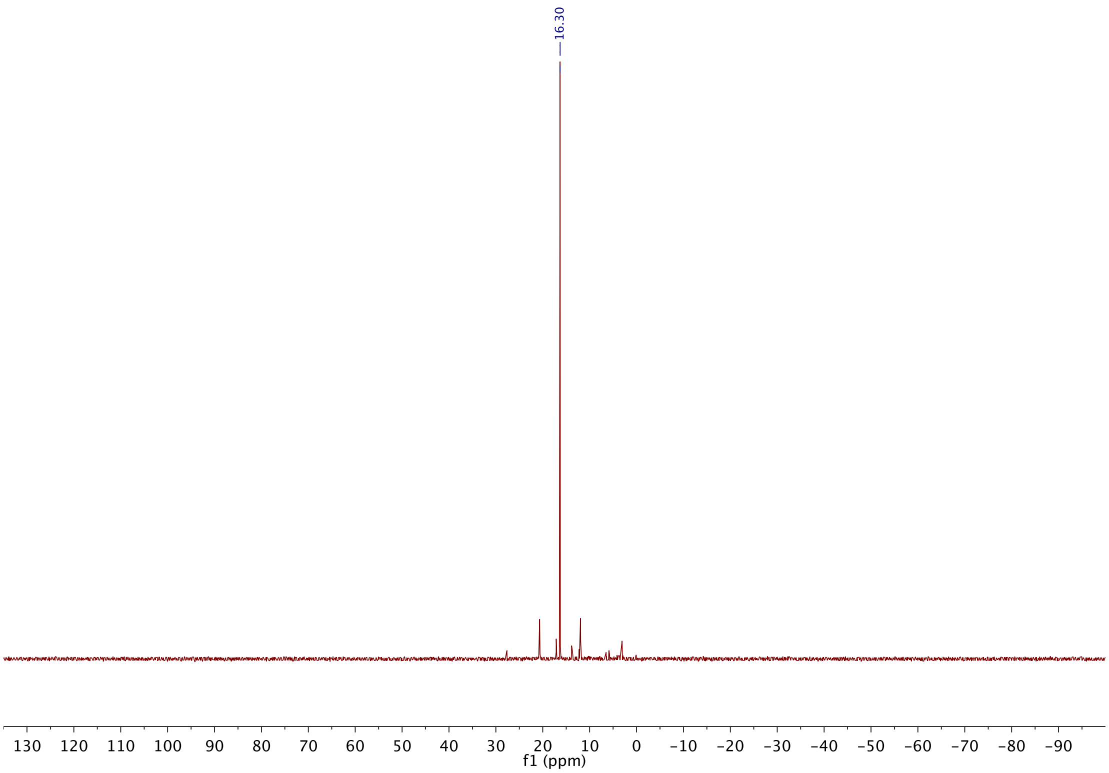

Figure S37. ^{31}P NMR spectrum of **[E(3,3)]PtAr₂**.
